# Dynamic changes of urine proteome in a Walker 256 tumor‐bearing rat model

**DOI:** 10.1002/cam4.1225

**Published:** 2017-10-04

**Authors:** Jianqiang Wu, Zhengguang Guo, Youhe Gao

**Affiliations:** ^1^ Department of Pathophysiology Institute of Basic Medical Sciences Chinese Academy of Medical Sciences School of Basic Medicine Peking Union Medical College Beijing 100005 China; ^2^ Core Facility of Instrument Institute of Basic Medical Sciences Chinese Academy of Medical Sciences School of Basic Medicine Peking Union Medical College Beijing 100005 China; ^3^ Department of Biochemistry and Molecular Biology Gene Engineering and Biotechnology Beijing Key Laboratory Beijing Normal University Beijing 100875 China

**Keywords:** Animal, biomarkers, early detection of cancer, models, neoplasms, proteomics, urine

## Abstract

Despite advances in cancer treatments, early diagnosis of cancer is still the most promising way to improve outcomes. Without homeostatic control, urine reflects systemic changes in the body and can potentially be used for early detection of cancer. In this study, a tumor‐bearing rat model was established by subcutaneous injection of Walker 256 cells. Urine samples from tumor‐bearing rats were collected at five time points during cancer development. Dynamic urine proteomes were profiled using liquid chromatography coupled with tandem mass spectrometry (LC‐MS/MS). Several urine proteins that changed at multiple time points were selected as candidate cancer biomarkers and were further validated by multiple reaction monitoring (MRM) analysis. It was found that the urinary protein patterns changed significantly with cancer development in a tumor‐bearing rat model. A total of 10 urinary proteins (HPT, APOA4, CO4, B2MG, A1AG, CATC, VCAM1, CALB1, CSPG4, and VTDB) changed significantly even before a tumor mass was palpable, and these early changes in urine could also be identified with differential abundance at late stages of cancer. Our results indicate that urine proteins could enable early detection of cancer at an early onset of tumor growth and monitoring of cancer progression.

## Introduction

Cancer is an important public health concern worldwide and is the second leading cause of death in the United States 1. The early detection of in situ or invasive carcinoma may prevent cancerous metastatic processes; thus, early detection can significantly improve survival rates for cancer patients. Despite technical advances in cancer diagnosis in the last decade, many cancer patients still cannot be diagnosed at early disease stages. To reduce mortality from cancer, novel approaches must be considered for early detection of cancer.

Cancer biomarkers are measurable changes associated with the pathophysiological processes of cancers that have the potential to diagnose cancer, monitor cancer progression, predict cancer recurrence, and assess treatment efficacy. Urine is a noninvasive and attractive biofluid for biomarker research. It is easy to collect large amounts of urine from patients for longitudinal studies. Without control of the homeostatic mechanism, urine accumulates systemic changes in the body and has the potential to reflect early and small pathological changes 2, 3. In recent years, urinary proteomics has been applied to discover biomarkers for cancer diagnosis and cancer monitoring 4, 5, 6. However, it is unclear whether time‐course analyses of urine proteins at different disease phases can reveal reliable biomarkers to monitor cancer progression and whether urine proteins assist in the noninvasive early detection of cancer at an early onset of tumor growth.

As the urine proteome can be affected by various factors, identifying the specific changes in urine associated with pathological conditions of cancer remains challenging. To circumvent this issue, animal models can be used to establish a direct relationship between cancer progression and corresponding protein changes in urine. The effects of genetic and environmental factors on the urine proteome are limited to the minimum. More importantly, the exact starting point of the disease is available, which is very helpful in the identification of biomarkers in the early stage of cancer.

**Figure 1:**
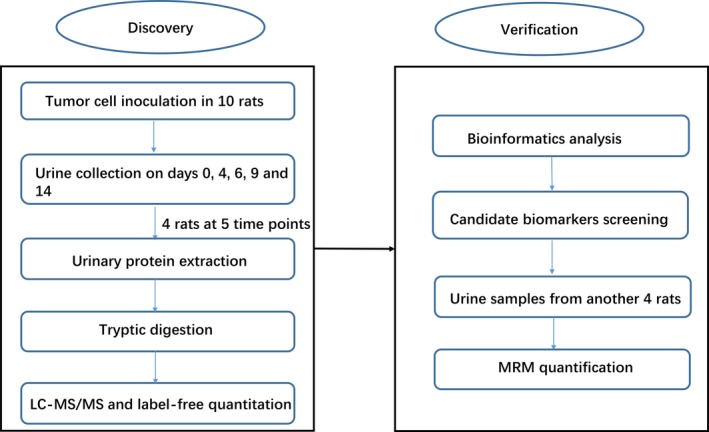
Workflow of urinary proteomics discovery and verification in this study. Urine samples were collected on days 0, 4, 6, 9, and 14 after Walker 256 cell inoculation, and the urinary proteome was analyzed using liquid chromatography coupled with tandem mass spectrometry (LC‐MS/MS) identification. Some candidate tumor biomarkers dynamically changed with tumor progression and were verified by multiple reaction monitoring.

The Walker 256 (W256) tumor‐bearing model is a well‐known cancer model with which to study tumor growth and cancer‐induced cachexia 7. In this study, a tumor‐bearing rat model was established by subcutaneous injection of W256 tumor cells. To identify changes in the urinary proteome during cancer development, urine samples from tumor‐bearing rats were collected at five time points. The workflow of the proteomic analysis in this study is shown in Figure 1. Using label‐free proteomics analysis and multiple reaction monitoring (MRM)‐based validation, cancer‐associated urine biomarkers were identified.

## Materials and Methods

### Experimental animals and model establishment

Male Wistar rats (150 ± 20 g) were supplied by the Institute of Laboratory Animal Science, Chinese Academy of Medical Science. All animals were maintained with free access to a standard laboratory diet and water with a 12‐h light–dark cycle under controlled indoor temperature (22 ± 2°C) and humidity (65–70%) conditions. Animal procedures were approved by the Institute of Basic Medical Sciences Animal Ethics Committee, Peking Union Medical College (ID: ACUC‐A02‐2014‐007), and the study was performed according to guidelines developed by the Institutional Animal Care and Use Committee of Peking Union Medical College.

A subcutaneous tumor‐bearing animal model was established as previously reported 8. Walker 256 (W256) carcinosarcoma cells were obtained from Cell Culture Center of Chinese Academy of Medical Sciences (Beijing, China) and were inoculated intraperitoneally into Wistar rats. Seven days following inoculation, the ascitic tumor cells were harvested from the peritoneal cavity. W256 tumor cells used for establishing the animal model were obtained from the ascitic fluid after two cell passages. Then, W256 cells were collected, centrifuged, and resuspended in phosphate‐buffered saline (PBS). The viability of W256 cells was evaluated by the Trypan blue exclusion test using a Neubauer chamber. It was observed that more than 95% tumor cells were viable. The rats were randomly divided into two groups: tumor‐bearing rats (*n* = 10) and control rats (*n* = 5). Tumor‐bearing rats were subcutaneously inoculated with 2 × 10^6^ viable W256 cells in 200 *μ*L of PBS into the right flank of the animal. An equal volume of PBS was subcutaneously inoculated into the control rats. During inoculation procedures, the animals were anesthetized with sodium pentobarbital solution (4 mg/kg).

### Urine collection and sample preparation

After the rats were acclimated in metabolic cages for 3 days, urine samples were collected from each rat on days 0, 4, 6, 9, 11, and 14 after tumor cell or PBS inoculation. Animals were individually placed in metabolic cages for 8 h to collect urine samples. During urine collection, rats had free access to water but no food to avoid urine contamination.

After urine collection, urine samples were immediately centrifuged at 12,000 g for 30 min at 4°C to remove cell debris. The supernatants were precipitated with three volumes of ethanol at 4°C, followed by centrifugation at 12,000 g for 30 min. The pellet was then resuspended in lysis buffer (8 mol/L urea, 2 mol/L thiourea, 50 mmol/L Tris, and 25 mmol/L DTT). The protein concentration of each sample was measured using the Bradford assay.

### SDS‐PAGE analysis

For each sample, on day 0, 4, 6, 9, 11, and 14 after W256 cell inoculation, 30 *μ*g of protein was added to the sample loading buffer (50 mmol/L Tris‐HCl, pH 6.8, 50 mol/L DTT, 0.5% SDS, and 10% glycerol) and incubated at 97°C for 10 min. The proteins were then resolved by 12% sodium dodecyl sulfate‐polyacrylamide gel electrophoresis (SDS‐PAGE). Urine protein samples from four randomly selected tumor‐bearing rats were used for SDS‐PAGE.

### Tryptic digestion

The urine samples on days 0, 4, 6, 9, and 14 of four tumor‐bearing rats after W256 cell inoculation were randomly selected for proteomic analysis. The urinary proteins were prepared using the FASP method as previously described 9. Each 100 *μ*g of protein was denatured with 20 mmol/L dithiothreitol at 37°C for 1 h and alkylated with 50 mmol/L iodoacetamide in the dark for 30 min. Then, samples were loaded onto 10‐kD filter devices (Pall, Port Washington, NY) and centrifuged at 14,000 g at 18°C. After washing twice with UA (8 mol/L urea in 0.1 mol/L Tris‐HCl, pH 8.5) and four times with 25 mmol/L NH_4_HCO_3_, the samples were digested with trypsin (enzyme to protein ratio of 1:50) at 37°C overnight. The peptide mixtures were desalted using Oasis HLB cartridges (Waters, Milford, MA) and dried by vacuum evaporation.

### LC‐MS/MS analysis

The 20 peptide samples resulting from the above digestion were re‐dissolved in 0.1% formic acid to a concentration of 0.5 *μ*g/*μ*L. For analysis, 1 *μ*g of peptides from an individual sample was loaded onto a trap column and was separated on a reverse‐phase C18 column (75 *μ*m × 100 mm, 2 *μ*m) using the EASY‐nLC 1200 HPLC system (Thermo Fisher Scientific, Waltham, MA). The elution for the analytical column was over 60 min at a flow rate of 300 nL/min. Then, the peptides were analyzed with an Orbitrap Fusion Lumos Tribrid mass spectrometer (Thermo Fisher Scientific, Waltham, MA). MS data were acquired in high‐sensitivity mode using the following parameters: data‐dependent MS/MS scans per full scan with top‐speed mode (3 sec), MS scans at a resolution of 120,000 and MS/MS scans at a resolution of 30,000 in Orbitrap, 30% HCD collision energy, charge‐state screening (+2 to +7), dynamic exclusion (exclusion duration 30 sec), and a maximum injection time of 45 ms.

### Label‐free proteome quantification

The proteomic data were searched against the SwissProt rat database (released in July 2016, containing 7973 sequences) using Mascot software (version 2.5.1, Matrix Science, London, UK). The parent ion tolerance was set to 10 ppm, and the fragment ion mass tolerance was set to 0.05 Da. Carbamidomethyl of cysteine was set as a fixed modification, and the oxidation of methionine was considered a variable modification. The specificity of trypsin digestion was set for cleavage after K or R, and two missed trypsin cleavage sites were allowed. Peptide and protein identification was further validated using Scaffold (version 4.4.0, Proteome Software Inc., Portland, OR). Peptide identifications were accepted at an FDR less than 1.0% by the Scaffold Local FDR algorithm, and protein identifications were accepted at an FDR less than 1.0% with at least two unique peptides. Comparisons across different samples were performed after normalization of total spectra accounts using Scaffold software. Spectral counting was used to compare protein abundance between different time points according to a previously described procedure 10, 11.

### Multiple reaction monitoring analysis

MRM was performed on a QTRAP‐6500 mass spectrometer (AB SCIEX, Framingham, MA) equipped with a nano‐UPLC system (Waters, Milford, MA). The peptides were eluted with 5–30% buffer B (0.1% formic acid, 99.9% ACN) at 300 nL/min for 60 min. The raw files of MS data acquired at the biomarker screening phase were used as the MS/MS spectral library to select peptides and transitions for the MRM assays. Mascot results and the list of targeted proteins were imported into Skyline software (version 3.6) to select the most intense peptide transitions. Then, a total of 120 *μ*g of peptides mixed from each validated sample was analyzed using a QTRAP‐6500 mass spectrometer (MS) to further select peptides and transitions for MRM validation of targeted proteins. Individual peptide samples from another four tumor‐bearing rats on days 0, 4, 6, 9, and 14 were analyzed by MRM assays. Each sample had three technical duplications. Unique peptides for each protein and 4–5 transitions per peptide were used for quantification. The length of a peptide candidate was 6−25 amino acids. MRM results were analyzed using the instructions from the Skyline software 12.

### Statistical analysis

The statistical analysis was performed with GraphPad Prism version 7.0 (GraphPad, San Diego, CA). Comparisons between five time points were conducted using repeated‐measures one‐way ANOVA followed by multiple comparisons analysis with the least significant difference (LSD) test. Group differences resulting in *P* < 0.05 were considered statistically significant.

## Results

### Characterization of tumor‐bearing rats

From 7 days after W256 cell subcutaneous inoculation, the average body weight of the tumor‐bearing rats was lower than that of the control rats (Fig. S1), and reduced food intake was observed in tumor‐bearing rats. On day 9 after W256 cell inoculation, the body weight of tumor‐bearing rats was significantly reduced compared with their body weights at other time points.

The growth of a subcutaneous tumor mass in tumor‐bearing rats was observed every day after W256 cell inoculation. Small tumor masses could be felt in the W256 rats beginning on the sixth day, and the tumor masses grew gradually. When the rats were killed after 15 days, the length (L) and width (W) of the tumors was measured. The volume (V) of the tumor was calculated with the formula *V* = 1/2 × L × W^2^. The average volume of tumor masses in 10 tumor‐bearing rats was 12.11 ± 4.84 cm^3^. The tumor tissues were then stained with hematoxylin and eosin (H&E) for pathological examination. Large numbers of tumor cells were observed in the tumor masses (Fig. S1).

### Urine proteome changed significantly with tumor progression

Urine samples from tumor‐bearing rats collected on different days were separated by 12% SDS‐PAGE. As shown in Figure S2, the protein patterns of urine samples in a representative tumor‐bearing rat changed significantly as the tumor progressed (day 0, day 4, day 6, day 9, day 11, and day 14). Similar patterns were observed in other rats, suggesting relatively good consistency in tumor progression. At the biomarker discovery phase, urine samples from four randomly selected tumor‐bearing rats at five time points (days 0, 4, 6, 9, and 14) were selected, and label‐free LC‐MS/MS quantification was used to characterize the differential expression of urinary proteins at multiple tumor progression stages. A total of 533 urinary proteins with at least two unique peptides were identified with <1% FDR at the protein level. All identification and quantification details are presented in Table S1.

**Figure 2:**
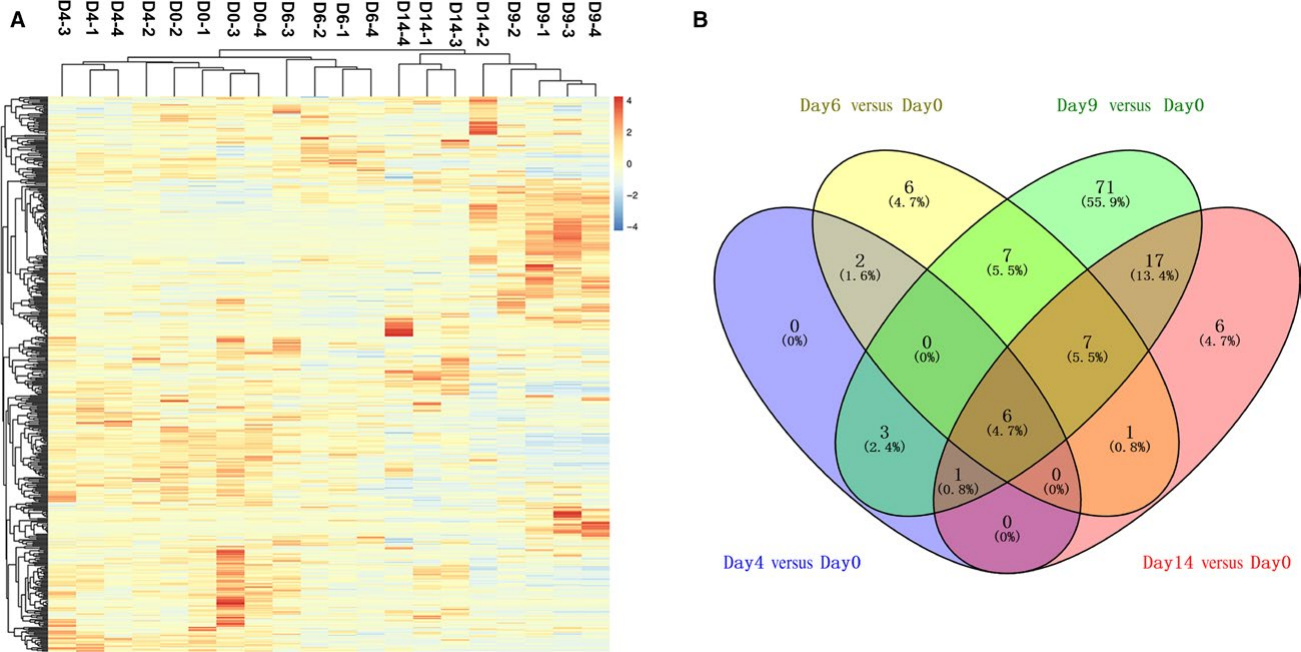
Proteomic analysis of the urine samples of tumor‐bearing rats at different phases. (A) Cluster analysis of the proteins identified by LC‐MS/MS. (B) Overlap evaluation of the differential proteins identified at different tumor phases.

After unsupervised clustering analysis of all urinary proteins identified, it was found that samples at each tumor stage were almost clustered together (Fig. 2A). The differential proteins were screened with the following criteria: fold change ≥1.5 and *P* < 0.05 compared with day 0, protein spectral counts from every rat in the high‐abundance group greater than those in the low‐abundance group, and an average spectral count in the high‐abundance group ≥4. The details of the differential proteins are shown in Table S2. The overlap of differential proteins identified at different tumor stages is shown by a Venn diagram (Fig. 2B). There were 12, 29, 112, and 38 differential proteins on days 4, 6, 9, and 14, respectively, after W256 cell inoculation.

Many proteins were commonly identified at different time points. Twelve differential proteins identified before tumor mass appearance on day 4 were also differentially expressed at later time points, including Galectin‐3‐binding protein, Complement C4, Beta‐2‐microglobulin, Haptoglobin, Macrophage colony‐stimulating factor 1, Coagulation factor XII and Apolipoprotein A‐IV. Importantly, six proteins (Haptoglobin, Apolipoprotein A‐IV, Complement C4, Beta‐2‐microglobulin, Alpha‐1‐acid glycoprotein, and Dipeptidyl peptidase 1) were significantly changed during the entire tumor progression with a fold change >1.5 and *P* < 0.05, suggesting the potential for these urine proteins to be used for the early detection of cancer.

### Functional analysis of differential urine proteins in tumor‐bearing rats

**Figure 3:**
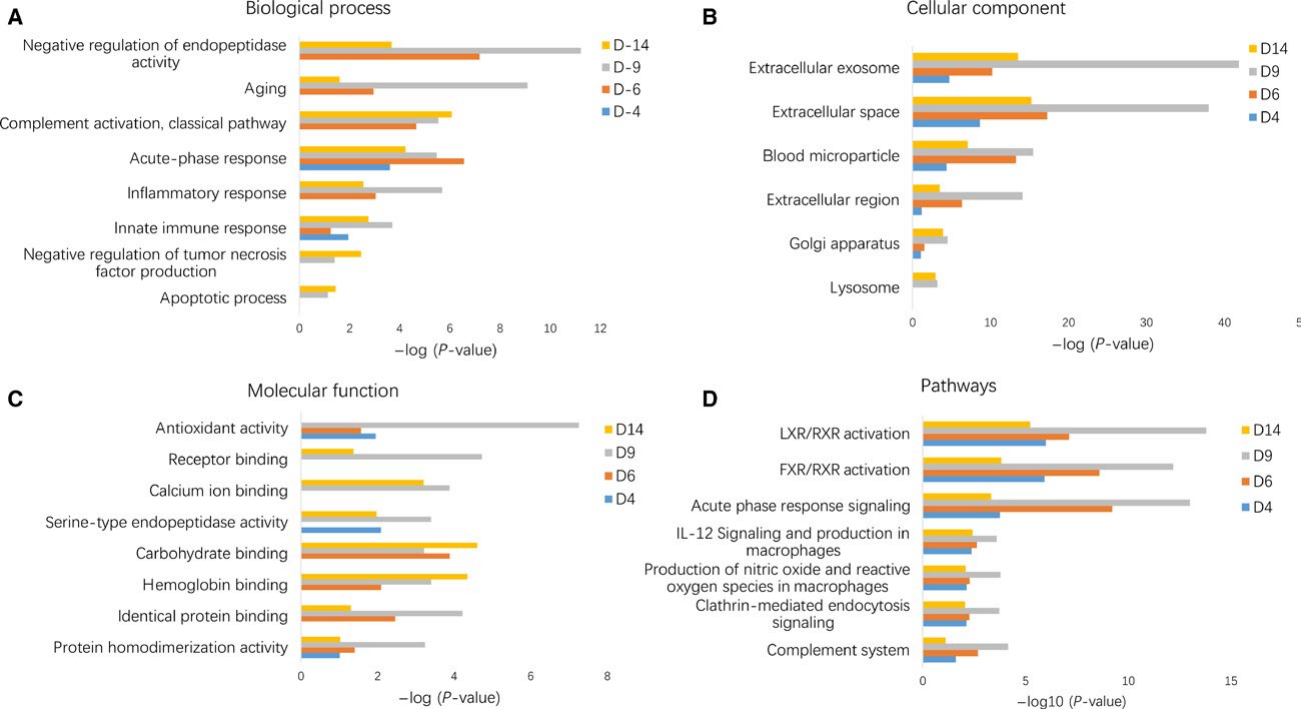
Functional analysis of differential proteins during cancer development. Dynamic changes of biological process (A), cellular component (B), molecular function (C), and pathway (D) at multiple time points were classified.

Functional annotation of differential proteins was performed using DAVID 13. Differential proteins at each time point were classified into biological process, molecular function, and molecular components (Fig. 3). In the biological process category, acute‐phase response and innate immune response were overrepresented at all the time points; complement activation, negative regulation of endopeptidase activity, aging and inflammatory response were overrepresented on days 6, 9, and 14; and negative regulation of tumor necrosis factor production was overrepresented on days 9 and 14 (Fig. 3A). In the cellular component category, the majority of differential proteins were extracellular exosome, extracellular space, blood microparticle, and extracellular region proteins, whereas a small number of differential proteins were also derived from organelles (Fig. 3B). In the molecular function category, antioxidant activity, receptor binding, and calcium ion and carbohydrate binding were overrepresented at multiple time points (Fig. 3C). To identify the major biological pathways involved with the differential urine proteins, IPA was used for canonical pathway enrichment analysis. It was demonstrated that LXR/RXR activation, acute‐phase response signaling, IL‐12 signaling and production in macrophages, production of nitric oxide and reactive oxygen species in macrophages, clathrin‐medicated endocytosis signaling and complement system were significantly enriched during tumor progression (Fig. 3D).

### MRM verification

**Figure 4:**
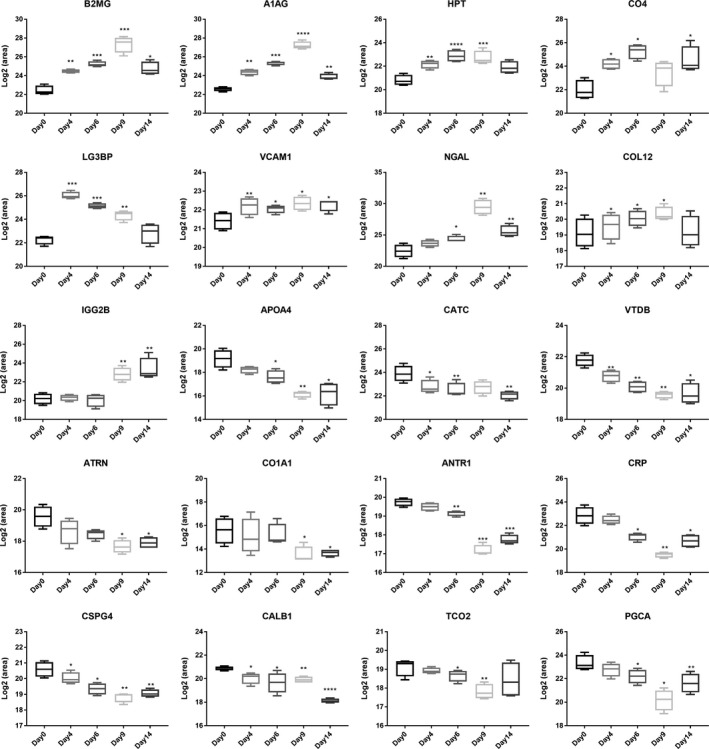
Expression of candidate urine biomarkers from tumor‐bearing rats by MRM quantification. Nine proteins shared an overall increasing trend in relative abundance. Eleven proteins shared an overall decreasing trend. The *x*‐axis represents different stages after tumor cell inoculation, and the *y*‐axis represents the log2 area of intensity based on MRM quantification.

At the biomarker validation phase, 30 differential proteins that changed dynamically were used for MRM verification. The details of these proteins are listed in Table 1. Then, urine samples from another four tumor‐bearing rats were randomly selected for MRM validation. A total of 25 proteins were successfully quantified, with the exception of LEG9, FA12, CSF1, LITH, and GAS6. Finally, 20 differential proteins were changed at multiple time points by MRM‐based quantification (Fig. 4). As a result, nine differential proteins showed an overall upregulated trend, including A1AG, B2MG, CO4, HPT, LG3BP, NGAL, COL12, IGG2B, and VCAM1. And 11 proteins showed an overall downregulated trend during tumor progression, including ANTR1, APOA4, ATRN, CALB1, CATC, CO1A1, CRP, CSPG4, PGCA, TCO2, and VTDB. The expression trends of these corresponding proteins were consistent with the results from label‐free quantification. Combined with label‐free and MRM quantification, a total of 10 urinary proteins (HPT, APOA4, CO4, B2MG, A1AG, CATC, VCAM1, CALB1, CSPG4, and VTDB) changed significantly even before a tumor mass was palpable, and these early changes in urine also showed differential abundance at late stages of cancer. The potential biomarker application of these 10 urine proteins are listed in Table 2.

**Table 1:** Differential urinary proteins selected for MRM validation

Accession	Protein description	Trends	*P*‐values	Average fold change			
				Day4	Day6	Day9	Day14
P07151	Beta‐2‐microglobulin (B2MG)^a^	↑	0.006	3.22	4.37	8.33	4.33
P02764	Alpha‐1‐acid glycoprotein (A1AG)	↑	0.007	1.81	2.49	6.05	1.77
P06866	Haptoglobin (HPT)	↑	0.007	2.88	5.06	3.31	3.56
P08649	Complement C4 (CO4)^a^	↑	0.028	3.40	4.66	6.14	3.57
O70513	Galectin‐3‐binding protein (LG3BP)^a^	↑	<0.001	6.77	4.55	2.32	1.55^b^
P29534	Vascular cell adhesion protein 1 (VCAM1)^a^	↑	<0.001	1.45^b^	1.73	2.58	2.00
Q8JZQ0	Macrophage colony‐stimulating factor 1 (CSF1)^a^	↑	0.063	2.08	2.08	2.31	1.92^b^
P30152	Neutrophil gelatinase‐associated lipocalin (NGAL)^a^	↑	0.005	1.06^b^	2.12	19.06	3.18^b^
Q4V885	Collectin‐12 (COL12)	↑	0.087	2.50	3.25	3.13^b^	1.75^b^
P47967	Galectin‐5 (LEG5)	↑	0.023	1.37^b^	1.59	3.52	3.07
P01048	Cluster of T‐kininogen 1 (KNT1)	↑	0.033	1.36^b^	1.97	3.61	3.03^b^
P97840	Galectin‐9 (LEG9)^a^	↑	0.022	5.5^b^	6^b^	13.50	14.00
P20761	Ig gamma‐2B chain C region (IGG2B)	↑	0.053	3.00^b^	1.00^b^	46.00	62.00
P10758	Lithostathine (LITH)	↑	0.030	∞	∞	∞	∞^b^
P02651	Apolipoprotein A‐IV (APOA4)^a^	↓	0.005	0.36	0.00	0.00	0.14
P80067	Dipeptidyl peptidase 1 (CATC)^a^	↓	<0.001	0.63	0.63	0.53	0.41
P04276	Vitamin D‐binding protein (VTDB)^a^	↓	0.031	0.59	0.41	0.26	0.40^b^
Q99J86	Attractin (ATRN)	↓	0.002	0.87^b^	0.58	0.13	0.53
P02454	Collagen alpha‐1(I) chain (CO1A1)^a^	↓	<0.001	0.75^b^	1.28^b^	0.16	0.09
Q0PMD2	Anthrax toxin receptor 1 (ANTR1)^a^	↓	0.004	0.79^b^	0.89^b^	0.14	0.39
P48199	C‐reactive protein (CRP)^a^	↓	<0.001	1.10^b^	0.43	0.17	0.45
Q00657	Chondroitin sulfate proteoglycan 4 (CSPG4)	↓	0.004	0.90^b^	0.71	0.24	0.36
Q9QZA2	Programmed cell death 6‐interacting protein (PDC6I)^a^	↓	0.005	0.37	0.33	0.04^b^	0.07^b^
Q63772	Growth arrest‐specific protein 6 (GAS6)^a^	↓	0.010	0.83^b^	0.58	0.13	0.33
P07171	Calbindin (CALB1)	↓	0.045	0.51	0.57^b^	0.31	0.00
P08289	Alkaline phosphatase, tissue‐nonspecific isozyme (PPBT)^a^	↓	0.004	0.85^b^	0.45^b^	0.20^b^	0.30
D3ZTE0	Coagulation factor XII (FA12)	↓	0.002	0.42	0.53	0.05	0.00
Q9R0D6	Transcobalamin‐2 (TCO2)	↓	0.044	0.75^b^	0.40	0.30	0.30
P07897	Aggrecan core protein (PGCA)	↓	0.003	0.83^b^	0.97^b^	0.13	0.20
Q9EQV6	Tripeptidyl‐peptidase 1 (TPP1)	↓	0.049	0.94^b^	0.44	0.75^b^	0.38

Comparisons between five time points were conducted using repeated‐measures one‐way ANOVA followed by multiple comparisons analysis. Average fold change is the average value from all four rats compared with day 0.

a Indicates that this protein was a cancer biomarker annotated in the IPA database or a candidate cancer biomarker from previous studies.

b Represents no statistical significance compared with day 0 (*P* > 0.05).

**Table 2:** Candidate urine biomarkers for early detection of cancer

Accession	Protein description	Urine marker^a^	Cancer biomarker use^b^
P06866	Haptoglobin (HPT)	Yes	Bladder, breast, lung, colorectal, ovarian, rectal, and gastric cancers
P07151	Beta‐2‐microglobulin (B2MG)	Yes	Colon, ovarian, and prostate cancers
P08649	Complement C4 (CO4)	Yes	Lung cancer
P02651	Apolipoprotein A‐IV (APOA4)	Yes	Bladder and ovarian cancers
P02764	Alpha‐1‐acid glycoprotein (A1AG)	Yes	Endometrial, bladder, and lung cancers
P80067	Dipeptidyl peptidase 1 (CATC)	‐	‐
P29534	Vascular cell adhesion protein 1 (VCAM1)	Yes	Lung, kidney, head and neck, and colorectal cancers
P07171	Calbindin (CALB1)	Yes	‐
Q00657	Chondroitin sulfate proteoglycan 4 (CSPG4)	‐	Breast cancer and melanoma
P04276	Vitamin D‐binding protein (VTDB)	Yes	Lung, prostate and gastric cancers

a Disease biomarkers detected in urine annotated in the Urinary Protein Biomarker Database 25.

b These proteins were reported as differential proteins in cancer patients.

## Discussion

In this study, a tumor‐bearing rat model was established by subcutaneous injection of W256 tumor cells. This model is a well‐known cancer model that has been extensively studied for tumor growth and cancer‐induced pain and cachexia 7, 8, 14. Urine samples were collected from tumor‐bearing rats at five different time points before and after cell inoculation. At the biomarker screening phase, the urinary proteome at different tumor stages was investigated by LC‐MS/MS and label‐free quantification. The urinary proteome changed significantly with tumor progression, and 127 differential proteins were identified at different cancer stages. At the biomarker validation phase, we selected 30 differential urine proteins that changed at multiple stages as reliable cancer biomarkers for MRM verification. Finally, 20 differential proteins were shown to change dynamically at multiple phases by MRM quantification. Moreover, the changes in these proteins were consistent with their corresponding changes in label‐free quantification.

Cancer is a major public health concern worldwide. There is an urgent need to develop noninvasive biomarkers for diagnosing and monitoring cancer progression, especially at early cancer stages. Urine is a good sample source for biomarker research because this biofluid accumulates changes, a hallmark that is the most fundamental property of biomarkers. In this study, using label‐free quantification, six proteins (B2MG, A1AG, HPT, CO4, APOA4, and CATC) were shown to change significantly during tumor development. Using MRM‐based quantification, it was found that VCAM1, CALB1, CSPG4, and VTDB also dynamically changed with tumor progression. These 10 proteins changed significantly even before a tumor mass was palpable and continued their corresponding trends during the entire tumor development process. At this stage, the body weights of tumor‐bearing rats were not obviously reduced, and the size of the tumor mass was not detected by imaging equipment. The results suggested the potential of these urine proteins in the early detection of tumors.

Proteomic changes in urine were probably mediated both by factors produced by the tumor and the host response to tumor‐bearing. Urine samples on day 9 had the greatest number of differential proteins, suggesting that the systemic response to tumors in the body is most intense at this stage. This result was consistent with the significantly changed protein patterns from the SDS‐PAGE analysis.

Interestingly, the pathways that changed in this experiment were similar to the pathways that were enriched in a previous urinary proteomics study in breast cancer patients 6. The common pathways included acute‐phase response signaling, LXR/RXR activation, production of nitric oxide and ROS in macrophages, IL‐12 signaling and production in macrophages, and clathrin‐medicated endocytosis signaling. Because W256 cells are mammary gland carcinoma cells 15, it is not a coincidence that there were common changed pathways between the W256 tumor‐bearing model and human breast cancer.

The W256 tumor‐bearing rat model has been previously used to study cancer‐induced cachexia. Cachexia was characterized by weight loss after tumor cell inoculation 16, 17. Our results are consistent with previous studies. Cancer cachexia affects approximately 50–80% of cancer patients and accounts for at least 20% of cancer‐associated deaths 18, 19. However, there are limited effective biomarkers of cachexia, especially in blood and urine. Cancer cachexia is a multifactorial and multi‐organ syndrome, and late‐stage cachexia is often irreversible. Urine accumulates systemic changes in the body and has the potential to reflect small and early pathological changes. Thus, urine would be a promising sample source of cachexia in future biomarker studies for the early detection of cachexia and elucidation of its pathophysiological process. Further clinical studies are needed in this field.

The biomarker filter function in IPA software was used to filter the candidate cancer biomarkers. Twenty‐four differential proteins in this experiment were identified as cancer biomarkers (Table S3). Several differential proteins in this study have also been reported in urine samples from cancer patients. For example, B2MG was found to be a urine marker of several cancers 20; PDC6I was a potential urinary biomarker of upper gastrointestinal cancer 21; and CO4 in human urine was reported to be helpful in the diagnosis of bladder cancer 22. A variety of malignant tumors consistently overexpressed NGAL with increased concentrations in urine, and NGAL is a potential biomarker for malignancy 23. Furthermore, KNT1 was validated to be a urine marker of breast cancer 24.

Overall, this study was a preliminary study with a small number of cancer‐bearing rats. Our results revealed that the urinary proteome changed significantly with tumor progression, and urine proteins could noninvasively indicate the presence of tumors at an early stage of tumor growth. In future studies, the urinary protein biomarkers identified require further evaluation in urine samples of cancer patients to test their sensitivity and specificity for early diagnosis of cancer, and they may also have potential applications in monitoring in cancer treatment and prevention studies.

## Conflict of Interest

The authors have declared that no competing interests exist.

## Supporting information


**Table S1**. Identification and quantitation details for the urine proteome.

**Table S2**. Differential proteins at different tumor stages.

**Table S3**. Cancer biomarkers annotated in the IPA database.

**Figure S1**. The body weights of tumor‐bearing rats and HE staining of tumor tissues.
**Figure S2**. Dynamic changes in protein patterns in the urine of tumor‐bearing rats.
