# Supplementary material for: Dynamic changes of urine proteome in a Walker 256 tumor‐bearing rat model
Source: Cancer Med. 2017 Oct 4;6(11):2713–22. doi: 10.1002/cam4.1225 (PMC5673914; doi:10.1002/cam4.1225)
Supplement: Supplementary file 1 — Table S1. Identification and quantitation details for the urine proteome. [file CAM4-6-2713-s002.docx]

**Table S1. Identification and quantitation details for the urine proteome.**

| **Bio View:533 Proteins in 498 Clusters With 5 Hidden** | **Accession Number** | **Molecular Weight** | **Rat1-0** | **Rat2-0** | **Rat3-0** | **Rat4-0** | **Rat1-4** | **Rat2-4** | **Rat3-4** | **Rat4-4** | **Rat1-6** | **Rat2-6** | **Rat3-6** | **Rat4-6** | **Rat1-9** | **Rat2-9** | **Rat3-9** | **Rat4-9** | **Rat1-14** | **Rat2-14** | **Rat3-14** | **Rat4-14** |
| --- | --- | --- | --- | --- | --- | --- | --- | --- | --- | --- | --- | --- | --- | --- | --- | --- | --- | --- | --- | --- | --- | --- |
| Cluster of Ig kappa chain C region, B allele OS=Rattus norvegicus PE=1 SV=1 | KACB_RAT | 12 kDa | 136 | 177 | 153 | 167 | 182 | 208 | 240 | 214 | 209 | 260 | 249 | 281 | 187 | 314 | 193 | 229 | 379 | 302 | 357 | 300 |
| Serum albumin OS=Rattus norvegicus GN=Alb PE=1 SV=2 | ALBU_RAT | 69 kDa | 217 | 214 | 219 | 202 | 178 | 163 | 207 | 173 | 144 | 135 | 139 | 149 | 147 | 130 | 182 | 185 | 144 | 151 | 159 | 248 |
| Cluster of Major urinary protein OS=Rattus norvegicus PE=1 SV=1 | MUP_RAT [2] | 21 kDa | 150 | 98 | 60 | 91 | 168 | 166 | 44 | 171 | 345 | 359 | 117 | 363 | 211 | 227 | 129 | 202 | 102 | 139 | 37 | 77 |
| Pro-epidermal growth factor OS=Rattus norvegicus GN=Egf PE=1 SV=2 | EGF_RAT | 124 kDa | 161 | 187 | 153 | 161 | 139 | 141 | 142 | 140 | 124 | 113 | 127 | 144 | 67 | 101 | 66 | 67 | 125 | 121 | 130 | 117 |
| Low-density lipoprotein receptor-related protein 2 OS=Rattus norvegicus GN=Lrp2 PE=1 SV=1 | LRP2_RAT | 519 kDa | 140 | 153 | 141 | 145 | 161 | 122 | 128 | 142 | 119 | 115 | 117 | 122 | 72 | 89 | 63 | 60 | 111 | 68 | 109 | 74 |
| Alpha-1-macroglobulin OS=Rattus norvegicus GN=A1m PE=1 SV=1 | A1M_RAT | 167 kDa | 141 | 116 | 121 | 129 | 132 | 101 | 119 | 116 | 116 | 87 | 100 | 105 | 80 | 78 | 65 | 57 | 112 | 59 | 98 | 93 |
| Urinary protein 2 OS=Rattus norvegicus PE=1 SV=1 | UP2_RAT | 11 kDa | 101 | 93 | 41 | 96 | 123 | 115 | 82 | 125 | 124 | 110 | 87 | 125 | 60 | 89 | 53 | 68 | 159 | 141 | 108 | 105 |
| Hemopexin OS=Rattus norvegicus GN=Hpx PE=1 SV=3 | HEMO_RAT | 51 kDa | 62 | 57 | 50 | 66 | 73 | 77 | 73 | 81 | 87 | 95 | 76 | 88 | 102 | 103 | 103 | 111 | 70 | 80 | 59 | 78 |
| Serine protease inhibitor A3K OS=Rattus norvegicus GN=Serpina3k PE=1 SV=3 | SPA3K_RAT | 47 kDa | 125 | 158 | 136 | 159 | 130 | 152 | 137 | 120 | 86 | 104 | 88 | 103 | 36 | 59 | 42 | 32 | 91 | 73 | 121 | 82 |
| Serine protease inhibitor A3L OS=Rattus norvegicus GN=Serpina3l PE=1 SV=3 | SPA3L_RAT | 46 kDa | 125 | 152 | 125 | 161 | 121 | 145 | 114 | 124 | 85 | 83 | 82 | 93 | 32 | 51 | 39 | 34 | 90 | 73 | 103 | 81 |
| Uromodulin OS=Rattus norvegicus GN=Umod PE=2 SV=1 | UROM_RAT | 71 kDa | 62 | 58 | 115 | 72 | 78 | 59 | 94 | 66 | 77 | 62 | 71 | 68 | 67 | 139 | 88 | 84 | 75 | 46 | 81 | 43 |
| Protein AMBP OS=Rattus norvegicus GN=Ambp PE=1 SV=1 | AMBP_RAT | 39 kDa | 66 | 71 | 56 | 65 | 61 | 70 | 48 | 52 | 69 | 79 | 70 | 65 | 84 | 60 | 100 | 70 | 69 | 100 | 52 | 59 |
| Cadherin-1 OS=Rattus norvegicus GN=Cdh1 PE=1 SV=1 | CADH1_RAT | 99 kDa | 66 | 50 | 67 | 71 | 76 | 69 | 78 | 76 | 73 | 68 | 73 | 69 | 43 | 63 | 50 | 44 | 81 | 52 | 80 | 60 |
| Serotransferrin OS=Rattus norvegicus GN=Tf PE=1 SV=3 | TRFE_RAT | 76 kDa | 77 | 72 | 67 | 71 | 59 | 53 | 63 | 49 | 63 | 54 | 52 | 64 | 63 | 39 | 71 | 70 | 49 | 54 | 55 | 93 |
| Cluster of Glandular kallikrein-7, submandibular/renal OS=Rattus norvegicus GN=Klk7 PE=1 SV=1 (KLK7_RAT) | KLK7_RAT [5] | 29 kDa | 80 | 75 | 67 | 76 | 46 | 80 | 50 | 55 | 65 | 76 | 91 | 54 | 47 | 75 | 28 | 48 | 57 | 84 | 64 | 70 |
| Urinary protein 1 OS=Rattus norvegicus PE=1 SV=1 | UP1_RAT | 11 kDa | 63 | 46 | 30 | 53 | 74 | 73 | 51 | 80 | 88 | 67 | 68 | 78 | 29 | 37 | 23 | 25 | 74 | 67 | 60 | 51 |
| Fibronectin OS=Rattus norvegicus GN=Fn1 PE=1 SV=2 | FINC_RAT | 273 kDa | 63 | 62 | 58 | 60 | 62 | 53 | 52 | 45 | 65 | 63 | 61 | 53 | 43 | 41 | 39 | 34 | 42 | 42 | 42 | 40 |
| Neutral and basic amino acid transport protein rBAT OS=Rattus norvegicus GN=Slc3a1 PE=1 SV=1 | SLC31_RAT | 79 kDa | 65 | 52 | 66 | 63 | 58 | 53 | 60 | 41 | 50 | 39 | 50 | 42 | 43 | 48 | 31 | 21 | 73 | 19 | 72 | 41 |
| Ig lambda-2 chain C region OS=Rattus norvegicus PE=4 SV=1 | LAC2_RAT | 11 kDa | 39 | 39 | 33 | 39 | 42 | 38 | 47 | 43 | 46 | 37 | 47 | 44 | 46 | 70 | 48 | 57 | 89 | 62 | 93 | 74 |
| Plasminogen OS=Rattus norvegicus GN=Plg PE=2 SV=2 | PLMN_RAT | 91 kDa | 48 | 43 | 41 | 38 | 34 | 46 | 44 | 42 | 53 | 56 | 53 | 50 | 46 | 40 | 63 | 50 | 44 | 68 | 49 | 45 |
| Urinary protein 3 OS=Rattus norvegicus PE=3 SV=1 | UP3_RAT | 11 kDa | 65 | 63 | 29 | 45 | 65 | 68 | 51 | 59 | 70 | 69 | 46 | 66 | 13 | 27 | 11 | 12 | 62 | 69 | 58 | 58 |
| Meprin A subunit alpha OS=Rattus norvegicus GN=Mep1a PE=1 SV=2 | MEP1A_RAT | 85 kDa | 51 | 49 | 51 | 47 | 62 | 46 | 49 | 52 | 47 | 39 | 50 | 46 | 27 | 46 | 26 | 24 | 48 | 38 | 58 | 46 |
| Polymeric immunoglobulin receptor OS=Rattus norvegicus GN=Pigr PE=1 SV=1 | PIGR_RAT | 85 kDa | 30 | 31 | 22 | 29 | 33 | 38 | 35 | 40 | 33 | 36 | 38 | 29 | 43 | 61 | 58 | 60 | 32 | 44 | 51 | 38 |
| Alpha-1-antiproteinase OS=Rattus norvegicus GN=Serpina1 PE=1 SV=2 | A1AT_RAT | 46 kDa | 37 | 37 | 28 | 35 | 30 | 33 | 33 | 28 | 24 | 27 | 20 | 26 | 46 | 46 | 68 | 61 | 16 | 56 | 20 | 42 |
| Alpha-1-acid glycoprotein OS=Rattus norvegicus GN=Orm1 PE=2 SV=1 | A1AG_RAT | 24 kDa | 14 | 14 | 13 | 16 | 24 | 27 | 22 | 30 | 35 | 41 | 35 | 31 | 71 | 71 | 93 | 110 | 25 | 30 | 21 | 25 |
| Cluster of T-kininogen 1 OS=Rattus norvegicus GN=Map1 PE=1 SV=2 (KNT1_RAT) | KNT1_RAT [3] | 48 kDa | 18 | 20 | 16 | 18 | 20 | 26 | 31 | 21 | 36 | 38 | 34 | 34 | 54 | 63 | 68 | 75 | 38 | 87 | 28 | 65 |
| Kallikrein-1 OS=Rattus norvegicus GN=Ngfg PE=2 SV=2 | KLK1_RAT | 29 kDa | 38 | 45 | 37 | 41 | 36 | 33 | 33 | 32 | 31 | 30 | 26 | 37 | 22 | 26 | 26 | 21 | 37 | 29 | 33 | 27 |
| Regenerating islet-derived protein 3-gamma OS=Rattus norvegicus GN=Reg3g PE=1 SV=1 | REG3G_RAT | 19 kDa | 30 | 23 | 13 | 21 | 42 | 41 | 34 | 47 | 50 | 58 | 39 | 54 | 28 | 52 | 44 | 32 | 22 | 58 | 20 | 24 |
| Complement C4 OS=Rattus norvegicus GN=C4 PE=1 SV=3 | CO4_RAT | 192 kDa | 8 | 12 | 7 | 8 | 23 | 41 | 29 | 26 | 41 | 52 | 35 | 35 | 59 | 34 | 62 | 60 | 25 | 42 | 25 | 33 |
| Urokinase-type plasminogen activator OS=Rattus norvegicus GN=Plau PE=1 SV=1 | UROK_RAT | 48 kDa | 34 | 38 | 40 | 34 | 37 | 33 | 37 | 35 | 28 | 25 | 37 | 30 | 29 | 28 | 32 | 36 | 38 | 30 | 43 | 31 |
| Alpha-2-HS-glycoprotein OS=Rattus norvegicus GN=Ahsg PE=1 SV=2 | FETUA_RAT | 38 kDa | 31 | 35 | 29 | 32 | 29 | 32 | 26 | 18 | 26 | 34 | 24 | 26 | 22 | 22 | 24 | 20 | 19 | 32 | 22 | 23 |
| Matrix-remodeling-associated protein 8 OS=Rattus norvegicus GN=Mxra8 PE=2 SV=1 | MXRA8_RAT | 43 kDa | 45 | 44 | 37 | 37 | 37 | 37 | 39 | 34 | 35 | 36 | 43 | 30 | 20 | 28 | 22 | 17 | 43 | 37 | 29 | 25 |
| Afamin OS=Rattus norvegicus GN=Afm PE=3 SV=1 | AFAM_RAT | 69 kDa | 30 | 32 | 31 | 42 | 31 | 31 | 32 | 38 | 28 | 28 | 28 | 33 | 28 | 33 | 31 | 27 | 25 | 28 | 24 | 35 |
| Gelsolin OS=Rattus norvegicus GN=Gsn PE=1 SV=1 | GELS_RAT | 86 kDa | 24 | 28 | 12 | 30 | 28 | 31 | 21 | 30 | 43 | 41 | 26 | 36 | 44 | 36 | 47 | 54 | 26 | 36 | 20 | 22 |
| Cluster of Secretoglobin family 2A member 2 OS=Rattus norvegicus GN=Scgb2a2 PE=1 SV=1 (SG2A2_RAT) | SG2A2_RAT [2] | 11 kDa | 32 | 29 | 29 | 28 | 9 | 30 | 18 | 20 | 20 | 34 | 75 | 28 | 28 | 35 | 9 | 22 | 19 | 48 | 30 | 41 |
| Dipeptidyl peptidase 4 OS=Rattus norvegicus GN=Dpp4 PE=1 SV=2 | DPP4_RAT | 88 kDa | 37 | 29 | 32 | 45 | 42 | 29 | 26 | 42 | 37 | 31 | 28 | 44 | 25 | 22 | 12 | 16 | 38 | 22 | 23 | 31 |
| Deoxyribonuclease-1 OS=Rattus norvegicus GN=Dnase1 PE=2 SV=1 | DNAS1_RAT | 32 kDa | 36 | 38 | 32 | 37 | 35 | 28 | 27 | 27 | 38 | 31 | 22 | 37 | 24 | 30 | 27 | 33 | 31 | 37 | 25 | 29 |
| Beta-2-microglobulin OS=Rattus norvegicus GN=B2m PE=1 SV=1 | B2MG_RAT | 14 kDa | 7 | 7 | 6 | 7 | 23 | 20 | 24 | 20 | 30 | 29 | 28 | 31 | 62 | 45 | 59 | 59 | 32 | 42 | 25 | 18 |
| Neutrophil gelatinase-associated lipocalin OS=Rattus norvegicus GN=Lcn2 PE=1 SV=2 | NGAL_RAT | 22 kDa | 6 | 6 | 2 | 3 | 4 | 3 | 6 | 5 | 10 | 12 | 5 | 9 | 84 | 61 | 98 | 81 | 14 | 27 | 5 | 8 |
| Glutamyl aminopeptidase OS=Rattus norvegicus GN=Enpep PE=1 SV=2 | AMPE_RAT | 108 kDa | 22 | 22 | 25 | 30 | 30 | 32 | 26 | 35 | 28 | 25 | 27 | 32 | 29 | 27 | 11 | 29 | 35 | 13 | 37 | 36 |
| Complement factor D OS=Rattus norvegicus GN=Cfd PE=1 SV=2 | CFAD_RAT | 28 kDa | 22 | 39 | 22 | 28 | 24 | 30 | 25 | 25 | 25 | 24 | 20 | 23 | 36 | 32 | 42 | 37 | 35 | 43 | 26 | 33 |
| Aminopeptidase N OS=Rattus norvegicus GN=Anpep PE=1 SV=2 | AMPN_RAT | 109 kDa | 29 | 24 | 32 | 33 | 36 | 32 | 45 | 31 | 33 | 25 | 40 | 31 | 21 | 26 | 16 | 16 | 32 | 12 | 42 | 25 |
| Cluster of Alpha-1-inhibitor 3 OS=Rattus norvegicus GN=A1i3 PE=1 SV=1 (A1I3_RAT) | A1I3_RAT [2] | 164 kDa | 48 | 52 | 71 | 55 | 31 | 33 | 51 | 30 | 11 | 8 | 16 | 9 | 1 | 0 | 1 | 3 | 5 | 1 | 21 | 53 |
| Cystatin-related protein 1 OS=Rattus norvegicus GN=Andpro PE=1 SV=1 | 22P1_RAT | 21 kDa | 41 | 40 | 32 | 28 | 18 | 39 | 15 | 17 | 31 | 36 | 46 | 29 | 21 | 26 | 10 | 16 | 20 | 43 | 20 | 29 |
| Galectin-3-binding protein OS=Rattus norvegicus GN=Lgals3bp PE=1 SV=2 | LG3BP_RAT | 64 kDa | 11 | 6 | 8 | 6 | 49 | 53 | 52 | 56 | 40 | 31 | 35 | 35 | 20 | 9 | 23 | 20 | 20 | 4 | 16 | 8 |
| Cluster of Actin, cytoplasmic 1 OS=Rattus norvegicus GN=Actb PE=1 SV=1 (ACTB_RAT) | ACTB_RAT [3] | 42 kDa | 30 | 29 | 28 | 26 | 33 | 19 | 27 | 28 | 30 | 24 | 30 | 23 | 18 | 23 | 20 | 16 | 30 | 19 | 41 | 28 |
| Pancreatic alpha-amylase OS=Rattus norvegicus GN=Amy2 PE=2 SV=2 | AMYP_RAT | 57 kDa | 26 | 30 | 19 | 19 | 17 | 15 | 15 | 14 | 17 | 13 | 20 | 23 | 14 | 16 | 12 | 11 | 43 | 18 | 59 | 72 |
| Serum amyloid P-component OS=Rattus norvegicus GN=Apcs PE=2 SV=2 | SAMP_RAT | 26 kDa | 20 | 25 | 24 | 27 | 29 | 27 | 26 | 23 | 22 | 26 | 18 | 28 | 22 | 23 | 20 | 21 | 27 | 21 | 23 | 18 |
| Prosaposin OS=Rattus norvegicus GN=Psap PE=1 SV=1 | SAP_RAT | 61 kDa | 22 | 27 | 30 | 26 | 24 | 21 | 23 | 28 | 28 | 21 | 30 | 26 | 25 | 15 | 19 | 24 | 27 | 18 | 30 | 23 |
| Complement C3 OS=Rattus norvegicus GN=C3 PE=1 SV=3 | CO3_RAT | 186 kDa | 23 | 31 | 23 | 22 | 15 | 15 | 30 | 16 | 5 | 13 | 12 | 8 | 37 | 10 | 42 | 27 | 6 | 19 | 21 | 74 |
| Prostatic steroid-binding protein C1 OS=Rattus norvegicus GN=Psbpc1 PE=1 SV=1 | PSC1_RAT | 13 kDa | 30 | 30 | 29 | 23 | 10 | 27 | 18 | 17 | 23 | 31 | 43 | 24 | 21 | 20 | 6 | 17 | 9 | 35 | 21 | 30 |
| Meprin A subunit beta OS=Rattus norvegicus GN=Mep1b PE=1 SV=3 | MEP1B_RAT | 79 kDa | 32 | 22 | 38 | 24 | 25 | 25 | 31 | 19 | 19 | 20 | 29 | 13 | 6 | 22 | 13 | 11 | 33 | 12 | 39 | 23 |
| Dipeptidyl peptidase 2 OS=Rattus norvegicus GN=Dpp7 PE=1 SV=1 | DPP2_RAT | 55 kDa | 34 | 28 | 30 | 31 | 24 | 19 | 23 | 22 | 22 | 19 | 26 | 25 | 9 | 19 | 14 | 16 | 12 | 10 | 21 | 15 |
| Superoxide dismutase [Cu-Zn] OS=Rattus norvegicus GN=Sod1 PE=1 SV=2 | SODC_RAT | 16 kDa | 10 | 9 | 6 | 10 | 11 | 12 | 10 | 10 | 14 | 14 | 12 | 15 | 52 | 38 | 52 | 60 | 21 | 38 | 14 | 15 |
| Cystatin-related protein 2 OS=Rattus norvegicus GN=Crp2 PE=2 SV=1 | 22P2_RAT | 21 kDa | 28 | 23 | 28 | 29 | 10 | 29 | 10 | 16 | 23 | 28 | 43 | 19 | 25 | 20 | 6 | 19 | 16 | 33 | 20 | 29 |
| EGF-containing fibulin-like extracellular matrix protein 1 OS=Rattus norvegicus GN=Efemp1 PE=1 SV=1 | FBLN3_RAT | 55 kDa | 22 | 21 | 20 | 21 | 25 | 20 | 19 | 18 | 23 | 31 | 25 | 23 | 17 | 17 | 22 | 18 | 15 | 18 | 13 | 14 |
| 6-phosphogluconolactonase OS=Rattus norvegicus GN=Pgls PE=1 SV=1 | 6PGL_RAT | 27 kDa | 19 | 14 | 13 | 14 | 18 | 15 | 14 | 15 | 16 | 14 | 16 | 13 | 36 | 21 | 26 | 28 | 28 | 43 | 20 | 26 |
| Vascular cell adhesion protein 1 OS=Rattus norvegicus GN=Vcam1 PE=2 SV=1 | VCAM1_RAT | 81 kDa | 12 | 13 | 9 | 6 | 19 | 13 | 16 | 10 | 19 | 19 | 18 | 13 | 28 | 28 | 23 | 24 | 26 | 23 | 18 | 13 |
| Carboxypeptidase Q OS=Rattus norvegicus GN=Cpq PE=1 SV=1 | CBPQ_RAT | 52 kDa | 15 | 15 | 10 | 17 | 20 | 17 | 16 | 20 | 16 | 20 | 22 | 33 | 21 | 11 | 15 | 21 | 12 | 10 | 12 | 11 |
| Gamma-glutamyl hydrolase OS=Rattus norvegicus GN=Ggh PE=1 SV=1 | GGH_RAT | 36 kDa | 20 | 20 | 15 | 19 | 22 | 16 | 16 | 16 | 17 | 18 | 15 | 15 | 13 | 21 | 8 | 11 | 15 | 22 | 15 | 9 |
| Gamma-glutamyltranspeptidase 1 OS=Rattus norvegicus GN=Ggt1 PE=1 SV=4 | GGT1_RAT | 62 kDa | 19 | 17 | 25 | 19 | 20 | 13 | 18 | 18 | 15 | 15 | 20 | 17 | 13 | 10 | 6 | 5 | 23 | 6 | 23 | 14 |
| Cathepsin B OS=Rattus norvegicus GN=Ctsb PE=1 SV=2 | CATB_RAT | 37 kDa | 9 | 13 | 9 | 11 | 13 | 14 | 10 | 16 | 17 | 25 | 8 | 15 | 24 | 32 | 33 | 33 | 9 | 26 | 8 | 11 |
| Prothrombin OS=Rattus norvegicus GN=F2 PE=1 SV=1 | THRB_RAT | 70 kDa | 15 | 12 | 19 | 16 | 16 | 14 | 15 | 12 | 19 | 17 | 16 | 18 | 15 | 13 | 16 | 15 | 16 | 22 | 16 | 20 |
| Cystatin-C OS=Rattus norvegicus GN=Cst3 PE=1 SV=2 | CYTC_RAT | 15 kDa | 14 | 18 | 6 | 13 | 17 | 21 | 13 | 15 | 21 | 19 | 11 | 15 | 23 | 17 | 24 | 24 | 15 | 26 | 15 | 10 |
| Class I histocompatibility antigen, Non-RT1.A alpha-1 chain OS=Rattus norvegicus GN=RT1-Aw2 PE=1 SV=1 | HA11_RAT | 37 kDa | 9 | 1 | 9 | 7 | 31 | 4 | 36 | 33 | 45 | 3 | 42 | 47 | 52 | 4 | 44 | 53 | 59 | 2 | 52 | 41 |
| Cathepsin D OS=Rattus norvegicus GN=Ctsd PE=1 SV=1 | CATD_RAT | 45 kDa | 19 | 18 | 19 | 17 | 13 | 15 | 17 | 17 | 13 | 7 | 14 | 17 | 13 | 15 | 11 | 12 | 19 | 22 | 16 | 18 |
| Prostatic spermine-binding protein OS=Rattus norvegicus GN=Sbp PE=1 SV=1 | SPBP_RAT | 31 kDa | 19 | 11 | 23 | 16 | 6 | 18 | 7 | 10 | 18 | 13 | 39 | 12 | 17 | 14 | 4 | 12 | 7 | 23 | 21 | 26 |
| Junctional adhesion molecule A OS=Rattus norvegicus GN=F11r PE=1 SV=1 | JAM1_RAT | 32 kDa | 20 | 14 | 18 | 19 | 14 | 12 | 15 | 12 | 18 | 13 | 13 | 13 | 23 | 24 | 21 | 18 | 14 | 12 | 14 | 13 |
| Neprilysin OS=Rattus norvegicus GN=Mme PE=1 SV=2 | NEP_RAT | 86 kDa | 16 | 16 | 23 | 20 | 19 | 18 | 15 | 15 | 15 | 19 | 27 | 12 | 6 | 10 | 3 | 2 | 11 | 14 | 21 | 19 |
| Vitamin D-binding protein OS=Rattus norvegicus GN=Gc PE=1 SV=3 | VTDB_RAT | 54 kDa | 29 | 26 | 35 | 22 | 19 | 13 | 22 | 12 | 13 | 11 | 12 | 10 | 6 | 4 | 10 | 9 | 9 | 5 | 9 | 22 |
| Lysosomal acid phosphatase OS=Rattus norvegicus GN=Acp2 PE=1 SV=1 | PPAL_RAT | 48 kDa | 13 | 14 | 19 | 17 | 13 | 10 | 16 | 16 | 15 | 12 | 20 | 15 | 18 | 14 | 15 | 12 | 15 | 11 | 16 | 14 |
| Ig gamma-2A chain C region OS=Rattus norvegicus GN=Igg-2a PE=1 SV=1 | IGG2A_RAT | 35 kDa | 14 | 11 | 13 | 19 | 20 | 10 | 13 | 18 | 16 | 13 | 12 | 18 | 17 | 9 | 17 | 16 | 46 | 25 | 39 | 55 |
| Carboxylesterase 1C OS=Rattus norvegicus GN=Ces1c PE=1 SV=3 | EST1C_RAT | 60 kDa | 17 | 27 | 24 | 19 | 15 | 22 | 18 | 17 | 7 | 12 | 12 | 9 | 5 | 8 | 9 | 9 | 12 | 4 | 17 | 17 |
| CD48 antigen OS=Rattus norvegicus GN=Cd48 PE=1 SV=1 | CD48_RAT | 28 kDa | 14 | 14 | 13 | 12 | 14 | 12 | 15 | 18 | 12 | 13 | 16 | 15 | 8 | 19 | 10 | 10 | 15 | 13 | 14 | 13 |
| Corticosteroid-binding globulin OS=Rattus norvegicus GN=Serpina6 PE=1 SV=2 | CBG_RAT | 45 kDa | 16 | 17 | 19 | 19 | 15 | 17 | 26 | 18 | 15 | 12 | 19 | 13 | 2 | 4 | 2 | 2 | 10 | 1 | 13 | 16 |
| Beta-glucuronidase OS=Rattus norvegicus GN=Gusb PE=2 SV=1 | BGLR_RAT | 75 kDa | 33 | 9 | 10 | 16 | 18 | 13 | 8 | 79 | 4 | 2 | 5 | 8 | 5 | 3 | 12 | 4 | 1 | 0 | 0 | 3 |
| Granulins OS=Rattus norvegicus GN=Grn PE=1 SV=3 | GRN_RAT | 63 kDa | 13 | 15 | 11 | 12 | 18 | 14 | 13 | 13 | 19 | 19 | 12 | 14 | 9 | 11 | 13 | 11 | 12 | 13 | 16 | 14 |
| Gamma-interferon-inducible lysosomal thiol reductase OS=Rattus norvegicus GN=Ifi30 PE=2 SV=1 | GILT_RAT | 28 kDa | 12 | 18 | 13 | 18 | 11 | 13 | 15 | 14 | 14 | 15 | 16 | 11 | 9 | 12 | 10 | 11 | 11 | 8 | 14 | 10 |
| Protein ABHD14B OS=Rattus norvegicus GN=Abhd14b PE=2 SV=1 | ABHEB_RAT | 23 kDa | 9 | 9 | 6 | 6 | 15 | 14 | 11 | 10 | 6 | 12 | 13 | 10 | 25 | 27 | 22 | 31 | 15 | 13 | 13 | 8 |
| Endothelial cell-selective adhesion molecule OS=Rattus norvegicus GN=Esam PE=1 SV=1 | ESAM_RAT | 42 kDa | 14 | 17 | 15 | 15 | 12 | 16 | 13 | 14 | 13 | 13 | 16 | 17 | 11 | 14 | 11 | 11 | 15 | 15 | 14 | 6 |
| Cubilin OS=Rattus norvegicus GN=Cubn PE=1 SV=2 | CUBN_RAT | 399 kDa | 14 | 20 | 19 | 23 | 16 | 12 | 18 | 11 | 4 | 3 | 11 | 4 | 5 | 9 | 8 | 6 | 10 | 1 | 11 | 4 |
| Monocyte differentiation antigen CD14 OS=Rattus norvegicus GN=Cd14 PE=2 SV=2 | CD14_RAT | 40 kDa | 8 | 12 | 11 | 16 | 15 | 17 | 15 | 19 | 14 | 13 | 16 | 12 | 11 | 10 | 11 | 11 | 12 | 9 | 13 | 15 |
| Di-N-acetylchitobiase OS=Rattus norvegicus GN=Ctbs PE=1 SV=1 | DIAC_RAT | 42 kDa | 10 | 17 | 15 | 15 | 15 | 16 | 13 | 13 | 13 | 12 | 15 | 12 | 10 | 11 | 9 | 9 | 10 | 14 | 14 | 16 |
| Clusterin OS=Rattus norvegicus GN=Clu PE=1 SV=2 | CLUS_RAT | 51 kDa | 9 | 11 | 11 | 12 | 11 | 11 | 14 | 13 | 3 | 7 | 11 | 9 | 21 | 14 | 14 | 20 | 9 | 22 | 10 | 16 |
| Fetuin-B OS=Rattus norvegicus GN=Fetub PE=2 SV=2 | FETUB_RAT | 42 kDa | 17 | 18 | 16 | 18 | 16 | 14 | 15 | 14 | 13 | 12 | 9 | 9 | 6 | 7 | 6 | 7 | 11 | 11 | 7 | 18 |
| WAP four-disulfide core domain protein 2 OS=Rattus norvegicus GN=Wfdc2 PE=2 SV=1 | WFDC2_RAT | 17 kDa | 11 | 12 | 11 | 13 | 14 | 11 | 12 | 9 | 15 | 14 | 13 | 9 | 15 | 12 | 11 | 9 | 15 | 17 | 12 | 13 |
| Retinoid-inducible serine carboxypeptidase OS=Rattus norvegicus GN=Scpep1 PE=2 SV=1 | RISC_RAT | 51 kDa | 12 | 7 | 8 | 11 | 10 | 11 | 14 | 11 | 14 | 14 | 15 | 17 | 17 | 11 | 12 | 17 | 9 | 9 | 9 | 5 |
| Haptoglobin OS=Rattus norvegicus GN=Hp PE=1 SV=3 | HPT_RAT | 39 kDa | 5 | 2 | 4 | 5 | 14 | 13 | 9 | 10 | 20 | 24 | 16 | 21 | 20 | 14 | 10 | 9 | 14 | 18 | 7 | 18 |
| Lysosomal alpha-glucosidase OS=Rattus norvegicus GN=Gaa PE=2 SV=1 | LYAG_RAT | 106 kDa | 13 | 10 | 12 | 10 | 14 | 11 | 15 | 10 | 22 | 12 | 13 | 15 | 6 | 7 | 5 | 3 | 4 | 5 | 2 | 5 |
| Cluster of Heat shock cognate 71 kDa protein OS=Rattus norvegicus GN=Hspa8 PE=1 SV=1 (HSP7C_RAT) | HSP7C_RAT [3] | 71 kDa | 14 | 7 | 15 | 22 | 5 | 11 | 9 | 6 | 6 | 7 | 19 | 3 | 11 | 9 | 12 | 6 | 10 | 9 | 12 | 13 |
| Sulfhydryl oxidase 1 OS=Rattus norvegicus GN=Qsox1 PE=1 SV=1 | QSOX1_RAT | 82 kDa | 14 | 11 | 9 | 6 | 13 | 9 | 13 | 11 | 4 | 4 | 6 | 10 | 48 | 24 | 14 | 9 | 5 | 5 | 5 | 8 |
| Ceruloplasmin OS=Rattus norvegicus GN=Cp PE=1 SV=3 | CERU_RAT | 121 kDa | 10 | 17 | 12 | 13 | 12 | 12 | 9 | 10 | 3 | 2 | 7 | 6 | 11 | 11 | 17 | 19 | 7 | 10 | 13 | 29 |
| Prostatic steroid-binding protein C2 OS=Rattus norvegicus GN=Psbpc2 PE=1 SV=1 | PSC2_RAT | 13 kDa | 8 | 9 | 9 | 7 | 1 | 12 | 6 | 7 | 8 | 13 | 17 | 11 | 13 | 10 | 5 | 7 | 5 | 17 | 9 | 16 |
| Serine protease inhibitor A3N OS=Rattus norvegicus GN=Serpina3n PE=1 SV=3 | SPA3N_RAT | 47 kDa | 7 | 9 | 8 | 7 | 7 | 7 | 14 | 8 | 4 | 4 | 7 | 3 | 25 | 7 | 45 | 32 | 4 | 16 | 4 | 10 |
| Nidogen-1 (Fragment) OS=Rattus norvegicus GN=Nid1 PE=1 SV=2 | NID1_RAT | 36 kDa | 13 | 16 | 13 | 12 | 7 | 13 | 10 | 9 | 15 | 13 | 11 | 10 | 13 | 14 | 9 | 9 | 16 | 12 | 12 | 6 |
| Neuroplastin OS=Rattus norvegicus GN=Nptn PE=1 SV=2 | NPTN_RAT | 44 kDa | 10 | 11 | 10 | 11 | 11 | 9 | 13 | 13 | 12 | 8 | 9 | 12 | 5 | 10 | 4 | 5 | 15 | 8 | 10 | 6 |
| Galectin-5 OS=Rattus norvegicus GN=Lgals5 PE=1 SV=2 | LEG5_RAT | 16 kDa | 8 | 6 | 7 | 6 | 8 | 8 | 11 | 10 | 14 | 10 | 10 | 9 | 22 | 25 | 25 | 23 | 22 | 32 | 14 | 15 |
| Protein-glutamine gamma-glutamyltransferase 4 OS=Rattus norvegicus GN=Tgm4 PE=1 SV=2 | TGM4_RAT | 76 kDa | 14 | 34 | 9 | 0 | 3 | 8 | 11 | 10 | 5 | 11 | 0 | 14 | 35 | 13 | 2 | 12 | 5 | 0 | 2 | 6 |
| Probasin OS=Rattus norvegicus GN=Pbsn PE=1 SV=1 | PBAS_RAT | 21 kDa | 13 | 6 | 7 | 5 | 9 | 21 | 15 | 11 | 8 | 3 | 9 | 8 | 9 | 7 | 3 | 22 | 7 | 9 | 22 | 17 |
| Extracellular superoxide dismutase [Cu-Zn] OS=Rattus norvegicus GN=Sod3 PE=1 SV=2 | SODE_RAT | 27 kDa | 9 | 9 | 8 | 8 | 9 | 9 | 8 | 8 | 10 | 7 | 9 | 6 | 20 | 13 | 22 | 19 | 15 | 12 | 11 | 10 |
| Tissue alpha-L-fucosidase OS=Rattus norvegicus GN=Fuca1 PE=1 SV=1 | FUCO_RAT | 53 kDa | 11 | 12 | 11 | 10 | 9 | 7 | 13 | 12 | 13 | 12 | 16 | 15 | 9 | 10 | 9 | 9 | 14 | 10 | 14 | 4 |
| Apolipoprotein E OS=Rattus norvegicus GN=Apoe PE=1 SV=2 | APOE_RAT | 36 kDa | 13 | 12 | 14 | 12 | 10 | 11 | 10 | 14 | 7 | 10 | 11 | 11 | 0 | 1 | 1 | 0 | 14 | 4 | 18 | 19 |
| Nucleobindin-1 OS=Rattus norvegicus GN=Nucb1 PE=1 SV=1 | NUCB1_RAT | 54 kDa | 14 | 9 | 11 | 13 | 13 | 12 | 13 | 12 | 6 | 7 | 10 | 10 | 7 | 7 | 6 | 7 | 6 | 5 | 8 | 7 |
| Chondroitin sulfate proteoglycan 4 OS=Rattus norvegicus GN=Cspg4 PE=1 SV=2 | CSPG4_RAT | 252 kDa | 14 | 14 | 15 | 16 | 14 | 13 | 14 | 12 | 10 | 7 | 13 | 12 | 2 | 7 | 2 | 3 | 4 | 6 | 7 | 4 |
| Cluster of Alpha-enolase OS=Rattus norvegicus GN=Eno1 PE=1 SV=4 (ENOA_RAT) | ENOA_RAT | 47 kDa | 12 | 5 | 15 | 7 | 8 | 7 | 12 | 8 | 6 | 10 | 7 | 3 | 8 | 13 | 9 | 9 | 10 | 8 | 10 | 11 |
| Thioredoxin OS=Rattus norvegicus GN=Txn PE=1 SV=2 | THIO_RAT | 12 kDa | 4 | 3 | 5 | 6 | 4 | 7 | 5 | 7 | 10 | 15 | 10 | 9 | 21 | 13 | 25 | 21 | 4 | 9 | 1 | 4 |
| Na(+)/H(+) exchange regulatory cofactor NHE-RF1 OS=Rattus norvegicus GN=Slc9a3r1 PE=1 SV=3 | NHRF1_RAT | 39 kDa | 5 | 3 | 19 | 10 | 11 | 6 | 12 | 9 | 6 | 8 | 12 | 3 | 6 | 4 | 4 | 2 | 17 | 5 | 17 | 7 |
| Osteopontin OS=Rattus norvegicus GN=Spp1 PE=1 SV=2 | OSTP_RAT | 35 kDa | 9 | 6 | 19 | 6 | 18 | 15 | 7 | 15 | 6 | 6 | 12 | 3 | 15 | 5 | 11 | 7 | 5 | 8 | 2 | 3 |
| Sialate O-acetylesterase OS=Rattus norvegicus GN=Siae PE=1 SV=2 | SIAE_RAT | 60 kDa | 14 | 11 | 6 | 9 | 8 | 11 | 8 | 11 | 8 | 12 | 11 | 13 | 8 | 11 | 5 | 8 | 7 | 10 | 9 | 3 |
| Cadherin-2 OS=Rattus norvegicus GN=Cdh2 PE=1 SV=1 | CADH2_RAT | 100 kDa | 7 | 8 | 14 | 9 | 8 | 9 | 9 | 6 | 11 | 11 | 8 | 7 | 4 | 10 | 5 | 5 | 7 | 5 | 13 | 6 |
| Prostaglandin-H2 D-isomerase OS=Rattus norvegicus GN=Ptgds PE=1 SV=2 | PTGDS_RAT | 21 kDa | 5 | 6 | 6 | 7 | 8 | 7 | 6 | 8 | 7 | 6 | 8 | 8 | 6 | 10 | 8 | 13 | 5 | 4 | 3 | 4 |
| RT1 class I histocompatibility antigen, AA alpha chain OS=Rattus norvegicus PE=1 SV=2 | HA12_RAT | 42 kDa | 11 | 3 | 11 | 7 | 20 | 7 | 18 | 24 | 28 | 7 | 23 | 28 | 38 | 9 | 35 | 37 | 32 | 6 | 29 | 29 |
| Na(+)/H(+) exchange regulatory cofactor NHE-RF3 OS=Rattus norvegicus GN=Pdzk1 PE=1 SV=2 | NHRF3_RAT | 57 kDa | 13 | 8 | 27 | 13 | 16 | 11 | 11 | 12 | 3 | 1 | 11 | 2 | 1 | 1 | 1 | 0 | 20 | 0 | 11 | 3 |
| Dipeptidyl peptidase 1 OS=Rattus norvegicus GN=Ctsc PE=1 SV=3 | CATC_RAT | 52 kDa | 11 | 13 | 13 | 12 | 7 | 8 | 7 | 9 | 6 | 9 | 9 | 7 | 5 | 10 | 5 | 6 | 6 | 6 | 4 | 4 |
| C-reactive protein OS=Rattus norvegicus GN=Crp PE=1 SV=1 | CRP_RAT | 25 kDa | 11 | 11 | 11 | 14 | 16 | 13 | 9 | 13 | 6 | 3 | 7 | 4 | 2 | 2 | 2 | 2 | 6 | 3 | 5 | 7 |
| Copper transport protein ATOX1 OS=Rattus norvegicus GN=Atox1 PE=1 SV=1 | ATOX1_RAT | 7 kDa | 6 | 7 | 7 | 6 | 9 | 6 | 5 | 8 | 8 | 7 | 10 | 9 | 12 | 10 | 12 | 7 | 10 | 12 | 9 | 5 |
| Cluster of 14-3-3 protein zeta/delta OS=Rattus norvegicus GN=Ywhaz PE=1 SV=1 (1433Z_RAT) | 1433Z_RAT [5] | 28 kDa | 10 | 7 | 10 | 8 | 8 | 7 | 9 | 4 | 4 | 7 | 9 | 1 | 5 | 9 | 10 | 7 | 1 | 3 | 10 | 9 |
| Ezrin OS=Rattus norvegicus GN=Ezr PE=1 SV=3 | EZRI_RAT | 69 kDa | 17 | 9 | 20 | 18 | 12 | 13 | 13 | 12 | 10 | 12 | 26 | 13 | 13 | 7 | 3 | 3 | 19 | 16 | 17 | 22 |
| Biotinidase OS=Rattus norvegicus GN=Btd PE=2 SV=1 | BTD_RAT | 58 kDa | 6 | 10 | 11 | 10 | 9 | 12 | 10 | 7 | 5 | 9 | 8 | 8 | 4 | 8 | 4 | 5 | 2 | 5 | 8 | 3 |
| Apolipoprotein M OS=Rattus norvegicus GN=Apom PE=1 SV=2 | APOM_RAT | 22 kDa | 4 | 7 | 5 | 5 | 7 | 9 | 6 | 7 | 8 | 7 | 3 | 9 | 13 | 8 | 8 | 9 | 10 | 14 | 8 | 6 |
| Complement component C9 OS=Rattus norvegicus GN=C9 PE=2 SV=1 | CO9_RAT | 62 kDa | 0 | 1 | 2 | 1 | 2 | 4 | 5 | 4 | 2 | 7 | 0 | 4 | 20 | 13 | 35 | 24 | 2 | 13 | 1 | 8 |
| Basal cell adhesion molecule OS=Rattus norvegicus GN=Bcam PE=2 SV=1 | BCAM_RAT | 68 kDa | 7 | 8 | 6 | 8 | 5 | 6 | 8 | 7 | 6 | 6 | 6 | 7 | 10 | 12 | 11 | 10 | 6 | 3 | 4 | 6 |
| 1,2-dihydroxy-3-keto-5-methylthiopentene dioxygenase OS=Rattus norvegicus GN=Adi1 PE=2 SV=1 | MTND_RAT | 21 kDa | 4 | 3 | 4 | 4 | 5 | 5 | 3 | 3 | 3 | 6 | 6 | 4 | 18 | 14 | 13 | 15 | 6 | 22 | 3 | 4 |
| Nidogen-2 OS=Rattus norvegicus GN=Nid2 PE=2 SV=1 | NID2_RAT | 153 kDa | 10 | 12 | 11 | 6 | 7 | 7 | 9 | 5 | 10 | 8 | 9 | 2 | 8 | 7 | 7 | 7 | 7 | 4 | 5 | 4 |
| Nuclear transport factor 2 OS=Rattus norvegicus GN=Nutf2 PE=1 SV=1 | NTF2_RAT | 14 kDa | 2 | 2 | 4 | 2 | 3 | 3 | 4 | 1 | 6 | 2 | 5 | 2 | 13 | 10 | 15 | 10 | 10 | 29 | 7 | 6 |
| Odorant-binding protein OS=Rattus norvegicus GN=Obp1f PE=1 SV=1 | OBP_RAT | 20 kDa | 12 | 13 | 2 | 4 | 2 | 2 | 4 | 2 | 4 | 5 | 7 | 7 | 3 | 3 | 7 | 3 | 2 | 13 | 1 | 2 |
| Interleukin-4 receptor subunit alpha OS=Rattus norvegicus GN=Il4r PE=2 SV=2 | IL4RA_RAT | 87 kDa | 9 | 8 | 8 | 8 | 9 | 5 | 12 | 7 | 8 | 6 | 6 | 4 | 8 | 7 | 4 | 5 | 10 | 5 | 7 | 6 |
| CD166 antigen OS=Rattus norvegicus GN=Alcam PE=1 SV=1 | CD166_RAT | 65 kDa | 5 | 9 | 7 | 9 | 6 | 2 | 7 | 9 | 3 | 3 | 6 | 6 | 8 | 13 | 9 | 7 | 10 | 4 | 8 | 4 |
| CD59 glycoprotein OS=Rattus norvegicus GN=Cd59 PE=1 SV=2 | CD59_RAT | 14 kDa | 6 | 7 | 4 | 6 | 6 | 7 | 7 | 6 | 8 | 9 | 7 | 9 | 7 | 5 | 8 | 7 | 7 | 8 | 9 | 8 |
| Multiple inositol polyphosphate phosphatase 1 OS=Rattus norvegicus GN=Minpp1 PE=1 SV=3 | MINP1_RAT | 55 kDa | 7 | 9 | 10 | 6 | 6 | 8 | 6 | 7 | 6 | 9 | 7 | 4 | 5 | 8 | 7 | 5 | 4 | 4 | 7 | 3 |
| Glutamate--cysteine ligase catalytic subunit OS=Rattus norvegicus GN=Gclc PE=1 SV=2 | GSH1_RAT | 73 kDa | 7 | 8 | 16 | 10 | 11 | 7 | 8 | 7 | 6 | 7 | 9 | 7 | 3 | 2 | 2 | 0 | 10 | 0 | 10 | 3 |
| Attractin OS=Rattus norvegicus GN=Atrn PE=2 SV=1 | ATRN_RAT | 159 kDa | 11 | 9 | 8 | 10 | 11 | 7 | 9 | 6 | 6 | 5 | 5 | 6 | 1 | 4 | 0 | 0 | 6 | 5 | 5 | 4 |
| Macrophage colony-stimulating factor 1 OS=Rattus norvegicus GN=Csf1 PE=2 SV=1 | CSF1_RAT | 62 kDa | 1 | 5 | 4 | 3 | 7 | 7 | 7 | 6 | 6 | 8 | 9 | 4 | 8 | 10 | 6 | 6 | 5 | 10 | 4 | 6 |
| Neutrophil collagenase OS=Rattus norvegicus GN=Mmp8 PE=2 SV=1 | MMP8_RAT | 53 kDa | 1 | 3 | 1 | 3 | 2 | 3 | 1 | 3 | 2 | 3 | 2 | 3 | 11 | 11 | 10 | 13 | 9 | 22 | 3 | 8 |
| Isopentenyl-diphosphate Delta-isomerase 1 OS=Rattus norvegicus GN=Idi1 PE=2 SV=2 | IDI1_RAT | 26 kDa | 0 | 0 | 0 | 2 | 2 | 3 | 1 | 4 | 1 | 4 | 2 | 3 | 14 | 12 | 17 | 20 | 5 | 19 | 2 | 7 |
| Protein RoBo-1 OS=Rattus norvegicus PE=1 SV=1 | ROB1_RAT | 26 kDa | 6 | 4 | 3 | 6 | 9 | 4 | 5 | 7 | 5 | 3 | 4 | 9 | 10 | 5 | 5 | 9 | 11 | 4 | 7 | 9 |
| CD44 antigen OS=Rattus norvegicus GN=Cd44 PE=1 SV=2 | CD44_RAT | 56 kDa | 6 | 4 | 5 | 7 | 6 | 6 | 5 | 5 | 5 | 5 | 5 | 4 | 2 | 5 | 3 | 5 | 6 | 3 | 8 | 7 |
| Intercellular adhesion molecule 1 OS=Rattus norvegicus GN=Icam1 PE=2 SV=1 | ICAM1_RAT | 60 kDa | 4 | 4 | 3 | 5 | 5 | 5 | 4 | 4 | 6 | 5 | 5 | 2 | 12 | 15 | 13 | 12 | 6 | 4 | 9 | 6 |
| Protein deglycase DJ-1 OS=Rattus norvegicus GN=Park7 PE=1 SV=1 | PARK7_RAT | 20 kDa | 0 | 0 | 2 | 1 | 1 | 0 | 1 | 0 | 0 | 1 | 1 | 0 | 22 | 13 | 40 | 27 | 0 | 18 | 0 | 0 |
| Carbonic anhydrase 1 OS=Rattus norvegicus GN=Ca1 PE=1 SV=1 | CAH1_RAT | 28 kDa | 0 | 0 | 0 | 0 | 0 | 0 | 0 | 0 | 0 | 1 | 0 | 0 | 33 | 15 | 35 | 29 | 0 | 15 | 0 | 0 |
| Cadherin-related family member 5 OS=Rattus norvegicus GN=Cdhr5 PE=1 SV=1 | CDHR5_RAT | 91 kDa | 4 | 9 | 8 | 7 | 7 | 7 | 10 | 6 | 7 | 5 | 10 | 7 | 5 | 4 | 3 | 4 | 4 | 4 | 7 | 4 |
| Angiotensinogen OS=Rattus norvegicus GN=Agt PE=1 SV=1 | ANGT_RAT | 52 kDa | 3 | 4 | 3 | 2 | 5 | 4 | 3 | 4 | 5 | 11 | 2 | 6 | 11 | 5 | 13 | 15 | 5 | 5 | 3 | 5 |
| Ig gamma-2C chain C region OS=Rattus norvegicus PE=2 SV=1 | IGG2C_RAT | 37 kDa | 3 | 6 | 3 | 5 | 2 | 5 | 3 | 4 | 2 | 4 | 4 | 4 | 5 | 8 | 10 | 6 | 6 | 5 | 11 | 16 |
| Beta-hexosaminidase subunit beta OS=Rattus norvegicus GN=Hexb PE=2 SV=1 | HEXB_RAT | 62 kDa | 5 | 7 | 10 | 9 | 7 | 9 | 8 | 7 | 5 | 4 | 4 | 3 | 6 | 7 | 1 | 2 | 6 | 4 | 5 | 7 |
| Carbonic anhydrase 3 OS=Rattus norvegicus GN=Ca3 PE=1 SV=3 | CAH3_RAT | 29 kDa | 0 | 0 | 0 | 0 | 0 | 0 | 0 | 0 | 0 | 0 | 0 | 0 | 30 | 4 | 36 | 44 | 0 | 0 | 0 | 0 |
| Anionic trypsin-1 OS=Rattus norvegicus GN=Prss1 PE=1 SV=1 | TRY1_RAT | 26 kDa | 6 | 10 | 6 | 6 | 5 | 7 | 6 | 6 | 5 | 7 | 7 | 8 | 4 | 4 | 5 | 5 | 6 | 5 | 5 | 5 |
| Cluster of Keratin, type I cytoskeletal 10 OS=Rattus norvegicus GN=Krt10 PE=3 SV=1 (K1C10_RAT) | K1C10_RAT [5] | 57 kDa | 6 | 5 | 6 | 2 | 2 | 2 | 3 | 9 | 3 | 11 | 6 | 3 | 1 | 8 | 5 | 6 | 1 | 1 | 12 | 4 |
| Collagen alpha-1(I) chain OS=Rattus norvegicus GN=Col1a1 PE=1 SV=5 | CO1A1_RAT | 138 kDa | 8 | 11 | 7 | 6 | 6 | 9 | 3 | 6 | 11 | 11 | 10 | 9 | 1 | 3 | 1 | 0 | 1 | 1 | 1 | 0 |
| Protein FAM151A OS=Rattus norvegicus GN=Fam151a PE=2 SV=1 | F151A_RAT | 67 kDa | 3 | 5 | 7 | 5 | 6 | 5 | 6 | 4 | 5 | 4 | 9 | 6 | 2 | 4 | 2 | 2 | 4 | 3 | 4 | 2 |
| Collectin-12 OS=Rattus norvegicus GN=Colec12 PE=2 SV=1 | COL12_RAT | 82 kDa | 2 | 4 | 0 | 2 | 3 | 8 | 3 | 6 | 6 | 7 | 6 | 7 | 8 | 4 | 7 | 6 | 1 | 10 | 2 | 1 |
| Ig gamma-2B chain C region OS=Rattus norvegicus GN=Igh-1a PE=1 SV=1 | IGG2B_RAT | 36 kDa | 0 | 1 | 0 | 0 | 1 | 0 | 0 | 2 | 0 | 1 | 0 | 0 | 11 | 8 | 11 | 16 | 9 | 9 | 12 | 32 |
| Phosphatidylethanolamine-binding protein 1 OS=Rattus norvegicus GN=Pebp1 PE=1 SV=3 | PEBP1_RAT | 21 kDa | 2 | 3 | 3 | 1 | 3 | 2 | 3 | 4 | 1 | 4 | 3 | 1 | 11 | 9 | 22 | 19 | 1 | 5 | 1 | 2 |
| Transthyretin OS=Rattus norvegicus GN=Ttr PE=1 SV=1 | TTHY_RAT | 16 kDa | 7 | 5 | 6 | 6 | 7 | 5 | 6 | 8 | 6 | 4 | 6 | 4 | 1 | 1 | 1 | 1 | 6 | 4 | 8 | 6 |
| Annexin A1 OS=Rattus norvegicus GN=Anxa1 PE=1 SV=2 | ANXA1_RAT | 39 kDa | 5 | 8 | 7 | 7 | 5 | 5 | 6 | 9 | 1 | 0 | 5 | 1 | 1 | 1 | 2 | 0 | 9 | 0 | 7 | 5 |
| Cathepsin L1 OS=Rattus norvegicus GN=Ctsl PE=1 SV=2 | CATL1_RAT | 38 kDa | 9 | 5 | 8 | 6 | 4 | 5 | 8 | 3 | 4 | 1 | 6 | 3 | 3 | 4 | 8 | 7 | 2 | 8 | 2 | 2 |
| Glycosylation-dependent cell adhesion molecule 1 OS=Rattus norvegicus GN=Glycam1 PE=2 SV=1 | GLCM1_RAT | 15 kDa | 5 | 6 | 5 | 6 | 5 | 6 | 4 | 5 | 4 | 4 | 5 | 6 | 6 | 4 | 4 | 5 | 7 | 4 | 4 | 5 |
| Beta-mannosidase OS=Rattus norvegicus GN=Manba PE=2 SV=1 | MANBA_RAT | 101 kDa | 3 | 5 | 6 | 5 | 3 | 7 | 5 | 5 | 3 | 5 | 6 | 6 | 7 | 3 | 7 | 11 | 2 | 3 | 3 | 3 |
| Peroxiredoxin-5, mitochondrial OS=Rattus norvegicus GN=Prdx5 PE=1 SV=1 | PRDX5_RAT | 22 kDa | 2 | 1 | 5 | 2 | 1 | 0 | 2 | 1 | 1 | 0 | 1 | 1 | 16 | 7 | 23 | 12 | 1 | 19 | 1 | 3 |
| Anthrax toxin receptor 1 OS=Rattus norvegicus GN=Antxr1 PE=1 SV=2 | ANTR1_RAT | 62 kDa | 7 | 8 | 6 | 7 | 4 | 5 | 7 | 6 | 5 | 6 | 8 | 6 | 1 | 2 | 0 | 1 | 4 | 2 | 3 | 2 |
| Aggrecan core protein OS=Rattus norvegicus GN=Acan PE=1 SV=2 | PGCA_RAT | 221 kDa | 7 | 9 | 5 | 9 | 6 | 8 | 7 | 4 | 10 | 7 | 5 | 7 | 2 | 2 | 0 | 0 | 1 | 3 | 1 | 1 |
| Putative phospholipase B-like 2 OS=Rattus norvegicus GN=Plbd2 PE=1 SV=2 | PLBL2_RAT | 65 kDa | 4 | 2 | 3 | 6 | 4 | 7 | 3 | 4 | 2 | 4 | 6 | 3 | 2 | 1 | 1 | 3 | 2 | 1 | 2 | 1 |
| Phospholysine phosphohistidine inorganic pyrophosphate phosphatase OS=Rattus norvegicus GN=Lhpp PE=2 SV=1 | LHPP_RAT | 29 kDa | 5 | 4 | 3 | 5 | 3 | 5 | 5 | 4 | 2 | 2 | 6 | 4 | 9 | 9 | 7 | 6 | 5 | 6 | 3 | 5 |
| Cluster of Ribonuclease pancreatic gamma-type OS=Rattus norvegicus PE=3 SV=1 (RNS1G_RAT) | RNS1G_RAT [3] | 17 kDa | 0 | 8 | 2 | 0 | 0 | 3 | 10 | 0 | 0 | 8 | 12 | 0 | 3 | 19 | 14 | 0 | 0 | 9 | 13 | 0 |
| Arylsulfatase B OS=Rattus norvegicus GN=Arsb PE=2 SV=2 | ARSB_RAT | 59 kDa | 3 | 2 | 4 | 5 | 4 | 5 | 6 | 4 | 4 | 3 | 5 | 3 | 8 | 7 | 2 | 8 | 4 | 4 | 3 | 2 |
| Prostasin OS=Rattus norvegicus GN=Prss8 PE=2 SV=3 | PRSS8_RAT | 37 kDa | 4 | 4 | 4 | 5 | 5 | 5 | 4 | 3 | 5 | 7 | 5 | 6 | 3 | 9 | 4 | 5 | 6 | 3 | 8 | 5 |
| Beta-2-glycoprotein 1 OS=Rattus norvegicus GN=Apoh PE=2 SV=2 | APOH_RAT | 33 kDa | 7 | 2 | 4 | 7 | 5 | 5 | 3 | 5 | 5 | 3 | 3 | 3 | 6 | 3 | 5 | 3 | 2 | 4 | 3 | 5 |
| Cluster of Aminoacylase-1A OS=Rattus norvegicus GN=Acy1a PE=1 SV=1 (ACY1A_RAT) | ACY1A_RAT | 46 kDa | 4 | 2 | 4 | 3 | 4 | 1 | 3 | 1 | 2 | 7 | 3 | 0 | 4 | 5 | 2 | 3 | 11 | 1 | 7 | 4 |
| Calbindin OS=Rattus norvegicus GN=Calb1 PE=1 SV=2 | CALB1_RAT | 30 kDa | 6 | 11 | 11 | 7 | 2 | 7 | 5 | 4 | 10 | 6 | 2 | 2 | 2 | 2 | 3 | 4 | 0 | 0 | 0 | 0 |
| Biglycan OS=Rattus norvegicus GN=Bgn PE=2 SV=1 | PGS1_RAT | 42 kDa | 3 | 2 | 8 | 7 | 4 | 2 | 6 | 8 | 2 | 3 | 5 | 7 | 3 | 2 | 3 | 2 | 6 | 1 | 10 | 7 |
| Fructose-bisphosphate aldolase B OS=Rattus norvegicus GN=Aldob PE=1 SV=2 | ALDOB_RAT | 40 kDa | 5 | 2 | 8 | 3 | 5 | 5 | 6 | 6 | 2 | 3 | 5 | 3 | 2 | 5 | 3 | 2 | 11 | 5 | 7 | 8 |
| Prolactin-inducible protein homolog OS=Rattus norvegicus GN=Pip PE=2 SV=1 | PIP_RAT | 16 kDa | 4 | 6 | 2 | 3 | 5 | 3 | 5 | 6 | 5 | 4 | 4 | 6 | 2 | 5 | 4 | 2 | 6 | 4 | 3 | 2 |
| Frizzled-4 OS=Rattus norvegicus GN=Fzd4 PE=2 SV=1 | FZD4_RAT | 60 kDa | 6 | 5 | 4 | 6 | 5 | 4 | 3 | 4 | 5 | 4 | 5 | 3 | 3 | 4 | 3 | 3 | 5 | 4 | 4 | 1 |
| Follistatin-related protein 1 OS=Rattus norvegicus GN=Fstl1 PE=1 SV=1 | FSTL1_RAT | 35 kDa | 4 | 6 | 7 | 6 | 4 | 5 | 6 | 3 | 2 | 3 | 5 | 4 | 0 | 3 | 0 | 1 | 4 | 2 | 3 | 1 |
| Lithostathine OS=Rattus norvegicus GN=Reg1 PE=1 SV=1 | LITH_RAT | 19 kDa | 0 | 0 | 0 | 0 | 3 | 5 | 4 | 2 | 4 | 7 | 4 | 2 | 11 | 9 | 14 | 6 | 2 | 8 | 1 | 4 |
| SMR1 protein OS=Rattus norvegicus GN=Vcsa1 PE=1 SV=1 | SMR1_RAT | 16 kDa | 5 | 6 | 1 | 2 | 5 | 6 | 4 | 5 | 4 | 5 | 4 | 8 | 0 | 5 | 3 | 3 | 2 | 3 | 4 | 6 |
| Triosephosphate isomerase OS=Rattus norvegicus GN=Tpi1 PE=1 SV=2 | TPIS_RAT | 27 kDa | 2 | 4 | 5 | 3 | 3 | 4 | 3 | 4 | 1 | 2 | 4 | 1 | 4 | 7 | 7 | 8 | 4 | 1 | 4 | 3 |
| Cluster of Glyceraldehyde-3-phosphate dehydrogenase OS=Rattus norvegicus GN=Gapdh PE=1 SV=3 (G3P_RAT) | G3P_RAT | 36 kDa | 5 | 4 | 9 | 3 | 7 | 3 | 6 | 3 | 4 | 4 | 5 | 2 | 2 | 1 | 3 | 2 | 5 | 1 | 4 | 3 |
| Calmodulin OS=Rattus norvegicus GN=Calm1 PE=1 SV=2 | CALM_RAT | 17 kDa | 5 | 4 | 6 | 4 | 5 | 3 | 3 | 3 | 3 | 5 | 2 | 1 | 3 | 4 | 4 | 1 | 5 | 3 | 7 | 2 |
| Cartilage oligomeric matrix protein OS=Rattus norvegicus GN=Comp PE=1 SV=1 | COMP_RAT | 83 kDa | 4 | 4 | 2 | 5 | 4 | 6 | 3 | 4 | 4 | 3 | 1 | 3 | 6 | 0 | 7 | 6 | 1 | 2 | 1 | 0 |
| Cathepsin S OS=Rattus norvegicus GN=Ctss PE=2 SV=1 | CATS_RAT | 37 kDa | 1 | 1 | 1 | 2 | 2 | 5 | 3 | 2 | 2 | 3 | 1 | 3 | 5 | 4 | 5 | 6 | 2 | 4 | 2 | 2 |
| Trefoil factor 1 OS=Rattus norvegicus GN=Tff1 PE=3 SV=1 | TFF1_RAT | 9 kDa | 5 | 4 | 3 | 3 | 5 | 3 | 7 | 5 | 4 | 4 | 7 | 6 | 1 | 3 | 3 | 2 | 2 | 2 | 7 | 4 |
| Activin receptor type-1B OS=Rattus norvegicus GN=Acvr1b PE=2 SV=1 | ACV1B_RAT | 57 kDa | 6 | 5 | 5 | 6 | 4 | 6 | 6 | 3 | 5 | 4 | 6 | 7 | 0 | 0 | 1 | 0 | 2 | 5 | 2 | 4 |
| Sortilin OS=Rattus norvegicus GN=Sort1 PE=1 SV=3 | SORT_RAT | 91 kDa | 5 | 5 | 5 | 6 | 6 | 3 | 3 | 5 | 3 | 3 | 7 | 2 | 1 | 2 | 1 | 1 | 5 | 3 | 3 | 1 |
| Carbonic anhydrase 2 OS=Rattus norvegicus GN=Ca2 PE=1 SV=2 | CAH2_RAT | 29 kDa | 4 | 4 | 4 | 2 | 4 | 3 | 4 | 3 | 2 | 2 | 2 | 1 | 9 | 4 | 4 | 7 | 2 | 1 | 3 | 1 |
| Neogenin (Fragment) OS=Rattus norvegicus GN=Neo1 PE=1 SV=1 | NEO1_RAT | 151 kDa | 4 | 7 | 11 | 3 | 4 | 6 | 4 | 5 | 2 | 2 | 7 | 4 | 1 | 2 | 1 | 0 | 4 | 0 | 1 | 2 |
| Growth arrest-specific protein 6 OS=Rattus norvegicus GN=Gas6 PE=1 SV=1 | GAS6_RAT | 75 kDa | 5 | 5 | 9 | 5 | 6 | 4 | 7 | 3 | 2 | 3 | 7 | 2 | 1 | 0 | 1 | 1 | 2 | 1 | 3 | 2 |
| Ubiquitin-60S ribosomal protein L40 OS=Rattus norvegicus GN=Uba52 PE=1 SV=2 | RL40_RAT (+3) | 15 kDa | 3 | 3 | 5 | 3 | 4 | 3 | 2 | 2 | 2 | 3 | 3 | 3 | 2 | 3 | 2 | 3 | 2 | 6 | 2 | 2 |
| Prostatic glandular kallikrein-6 OS=Rattus norvegicus GN=Klk6 PE=3 SV=1 | KLK6_RAT | 29 kDa | 14 | 14 | 8 | 9 | 7 | 13 | 7 | 9 | 7 | 12 | 16 | 8 | 7 | 13 | 5 | 7 | 9 | 28 | 8 | 13 |
| Aquaporin-1 OS=Rattus norvegicus GN=Aqp1 PE=1 SV=4 | AQP1_RAT | 29 kDa | 3 | 4 | 4 | 4 | 6 | 4 | 3 | 4 | 5 | 3 | 4 | 4 | 1 | 3 | 2 | 1 | 6 | 1 | 4 | 6 |
| Alkaline phosphatase, tissue-nonspecific isozyme OS=Rattus norvegicus GN=Alpl PE=1 SV=2 | PPBT_RAT | 58 kDa | 4 | 3 | 8 | 5 | 6 | 4 | 4 | 3 | 3 | 2 | 3 | 1 | 2 | 2 | 0 | 0 | 2 | 0 | 2 | 2 |
| Peptidyl-prolyl cis-trans isomerase A OS=Rattus norvegicus GN=Ppia PE=1 SV=2 | PPIA_RAT | 18 kDa | 3 | 3 | 6 | 4 | 3 | 3 | 4 | 1 | 2 | 3 | 5 | 0 | 4 | 5 | 6 | 2 | 1 | 2 | 5 | 1 |
| Peroxiredoxin-6 OS=Rattus norvegicus GN=Prdx6 PE=1 SV=3 | PRDX6_RAT | 25 kDa | 4 | 2 | 5 | 3 | 4 | 5 | 3 | 2 | 1 | 2 | 5 | 1 | 2 | 7 | 2 | 1 | 2 | 1 | 4 | 2 |
| Inhibin beta C chain OS=Rattus norvegicus GN=Inhbc PE=2 SV=1 | INHBC_RAT | 39 kDa | 4 | 4 | 4 | 3 | 4 | 4 | 4 | 5 | 6 | 2 | 4 | 3 | 1 | 1 | 1 | 1 | 2 | 3 | 4 | 3 |
| Amyloid beta A4 protein OS=Rattus norvegicus GN=App PE=1 SV=2 | A4_RAT | 87 kDa | 5 | 4 | 4 | 3 | 5 | 3 | 3 | 4 | 3 | 4 | 2 | 3 | 2 | 1 | 2 | 2 | 6 | 2 | 2 | 2 |
| Pancreatic secretory granule membrane major glycoprotein GP2 OS=Rattus norvegicus GN=Gp2 PE=1 SV=1 | GP2_RAT | 59 kDa | 7 | 3 | 2 | 3 | 6 | 2 | 1 | 3 | 6 | 1 | 0 | 2 | 4 | 2 | 1 | 3 | 12 | 0 | 1 | 5 |
| Interleukin-1 receptor type 2 OS=Rattus norvegicus GN=Il1r2 PE=2 SV=1 | IL1R2_RAT | 46 kDa | 2 | 1 | 3 | 2 | 2 | 4 | 3 | 3 | 3 | 3 | 2 | 1 | 5 | 4 | 4 | 5 | 2 | 2 | 2 | 0 |
| Glutamate--cysteine ligase regulatory subunit OS=Rattus norvegicus GN=Gclm PE=1 SV=1 | GSH0_RAT | 31 kDa | 5 | 3 | 3 | 5 | 4 | 2 | 5 | 8 | 3 | 3 | 3 | 4 | 0 | 2 | 0 | 0 | 5 | 0 | 8 | 2 |
| Kynurenine/alpha-aminoadipate aminotransferase, mitochondrial OS=Rattus norvegicus GN=Aadat PE=1 SV=1 | AADAT_RAT | 48 kDa | 5 | 1 | 4 | 6 | 5 | 3 | 3 | 4 | 3 | 4 | 3 | 2 | 1 | 2 | 0 | 0 | 2 | 0 | 7 | 0 |
| Pro-cathepsin H OS=Rattus norvegicus GN=Ctsh PE=1 SV=1 | CATH_RAT | 37 kDa | 4 | 3 | 2 | 4 | 4 | 5 | 3 | 3 | 3 | 2 | 1 | 0 | 5 | 4 | 9 | 5 | 0 | 0 | 1 | 1 |
| Interleukin-1 receptor accessory protein OS=Rattus norvegicus GN=Il1rap PE=2 SV=1 | IL1AP_RAT | 66 kDa | 2 | 3 | 2 | 3 | 3 | 6 | 2 | 4 | 3 | 2 | 2 | 2 | 3 | 2 | 2 | 2 | 1 | 3 | 2 | 0 |
| Alpha-N-acetylgalactosaminidase OS=Rattus norvegicus GN=Naga PE=2 SV=1 | NAGAB_RAT | 47 kDa | 3 | 2 | 2 | 4 | 2 | 1 | 4 | 3 | 4 | 4 | 6 | 3 | 4 | 2 | 2 | 3 | 0 | 0 | 3 | 1 |
| Transcobalamin-2 OS=Rattus norvegicus GN=Tcn2 PE=2 SV=1 | TCO2_RAT | 47 kDa | 5 | 6 | 6 | 3 | 3 | 4 | 6 | 2 | 3 | 2 | 1 | 2 | 2 | 1 | 1 | 2 | 1 | 2 | 2 | 1 |
| Glutathione peroxidase 3 OS=Rattus norvegicus GN=Gpx3 PE=2 SV=2 | GPX3_RAT | 25 kDa | 2 | 4 | 2 | 3 | 4 | 3 | 3 | 3 | 2 | 3 | 3 | 1 | 2 | 0 | 2 | 1 | 2 | 1 | 1 | 1 |
| Apolipoprotein A-I OS=Rattus norvegicus GN=Apoa1 PE=1 SV=2 | APOA1_RAT | 30 kDa | 1 | 5 | 2 | 1 | 1 | 1 | 3 | 0 | 0 | 1 | 4 | 0 | 0 | 0 | 0 | 0 | 1 | 0 | 9 | 26 |
| Glutathione S-transferase omega-1 OS=Rattus norvegicus GN=Gsto1 PE=1 SV=2 | GSTO1_RAT | 28 kDa | 0 | 0 | 2 | 0 | 0 | 0 | 2 | 0 | 1 | 2 | 0 | 1 | 13 | 8 | 14 | 11 | 1 | 3 | 0 | 0 |
| Serine protease inhibitor A3M (Fragment) OS=Rattus norvegicus GN=Serpina3m PE=2 SV=1 | SPA3M_RAT | 46 kDa | 3 | 9 | 6 | 6 | 6 | 4 | 9 | 5 | 4 | 4 | 4 | 4 | 2 | 7 | 3 | 2 | 0 | 3 | 5 | 4 |
| Tripeptidyl-peptidase 1 OS=Rattus norvegicus GN=Tpp1 PE=1 SV=1 | TPP1_RAT | 61 kDa | 3 | 3 | 6 | 4 | 2 | 2 | 6 | 5 | 1 | 3 | 2 | 1 | 2 | 2 | 6 | 2 | 1 | 2 | 1 | 2 |
| Angiotensin-converting enzyme OS=Rattus norvegicus GN=Ace PE=1 SV=1 | ACE_RAT | 151 kDa | 1 | 2 | 7 | 1 | 2 | 2 | 8 | 3 | 1 | 2 | 6 | 1 | 1 | 1 | 1 | 1 | 0 | 0 | 5 | 0 |
| G-protein coupled receptor family C group 5 member C OS=Rattus norvegicus GN=Gprc5c PE=1 SV=2 | GPC5C_RAT | 48 kDa | 4 | 3 | 4 | 4 | 6 | 3 | 3 | 6 | 3 | 4 | 5 | 6 | 1 | 0 | 2 | 1 | 1 | 0 | 2 | 1 |
| Gastricsin OS=Rattus norvegicus GN=Pgc PE=1 SV=1 | PEPC_RAT | 43 kDa | 6 | 4 | 3 | 4 | 3 | 2 | 3 | 4 | 5 | 3 | 4 | 4 | 0 | 1 | 2 | 2 | 4 | 1 | 3 | 3 |
| Neural cell adhesion molecule 1 OS=Rattus norvegicus GN=Ncam1 PE=1 SV=1 | NCAM1_RAT | 95 kDa | 2 | 4 | 5 | 3 | 3 | 8 | 5 | 4 | 2 | 5 | 3 | 3 | 1 | 2 | 1 | 0 | 2 | 1 | 1 | 0 |
| Procollagen C-endopeptidase enhancer 1 OS=Rattus norvegicus GN=Pcolce PE=1 SV=1 | PCOC1_RAT | 50 kDa | 4 | 5 | 3 | 2 | 1 | 2 | 3 | 0 | 1 | 3 | 2 | 0 | 2 | 1 | 6 | 1 | 0 | 4 | 0 | 0 |
| Programmed cell death 6-interacting protein OS=Rattus norvegicus GN=Pdcd6ip PE=1 SV=2 | PDC6I_RAT | 97 kDa | 5 | 4 | 13 | 5 | 2 | 1 | 7 | 0 | 1 | 2 | 5 | 1 | 0 | 1 | 0 | 0 | 1 | 0 | 0 | 1 |
| Solute carrier family 23 member 1 OS=Rattus norvegicus GN=Slc23a1 PE=2 SV=1 | S23A1_RAT | 65 kDa | 3 | 0 | 5 | 5 | 3 | 3 | 4 | 1 | 2 | 0 | 5 | 2 | 0 | 2 | 0 | 0 | 4 | 0 | 5 | 2 |
| Cystatin-B OS=Rattus norvegicus GN=Cstb PE=1 SV=1 | CYTB_RAT | 11 kDa | 0 | 4 | 0 | 0 | 0 | 2 | 1 | 0 | 4 | 4 | 3 | 3 | 6 | 3 | 8 | 3 | 4 | 8 | 1 | 2 |
| Dihydrolipoyllysine-residue succinyltransferase component of 2-oxoglutarate dehydrogenase complex, mitochondrial OS=Rattus norvegicus GN=Dlst PE=1 SV=2 | ODO2_RAT | 49 kDa | 3 | 2 | 2 | 3 | 3 | 4 | 3 | 2 | 3 | 3 | 2 | 2 | 4 | 2 | 4 | 2 | 5 | 5 | 3 | 3 |
| D-dopachrome decarboxylase OS=Rattus norvegicus GN=Ddt PE=1 SV=3 | DOPD_RAT | 13 kDa | 0 | 0 | 0 | 0 | 0 | 1 | 0 | 0 | 0 | 1 | 1 | 1 | 15 | 7 | 15 | 15 | 1 | 1 | 1 | 0 |
| Protein disulfide-isomerase OS=Rattus norvegicus GN=P4hb PE=1 SV=2 | PDIA1_RAT | 57 kDa | 2 | 4 | 3 | 2 | 3 | 3 | 3 | 2 | 2 | 1 | 3 | 1 | 1 | 1 | 4 | 2 | 1 | 1 | 1 | 1 |
| Prostaglandin F2 receptor negative regulator OS=Rattus norvegicus GN=Ptgfrn PE=1 SV=1 | FPRP_RAT | 99 kDa | 5 | 3 | 5 | 5 | 4 | 3 | 4 | 4 | 4 | 2 | 3 | 3 | 2 | 0 | 1 | 0 | 0 | 1 | 2 | 0 |
| Solute carrier family 7 member 13 OS=Rattus norvegicus GN=Slc7a13 PE=2 SV=1 | S7A13_RAT | 54 kDa | 3 | 3 | 5 | 3 | 2 | 3 | 3 | 2 | 1 | 1 | 2 | 2 | 1 | 2 | 1 | 0 | 4 | 0 | 3 | 1 |
| Ribonuclease 4 OS=Rattus norvegicus GN=Rnase4 PE=1 SV=1 | RNAS4_RAT | 17 kDa | 1 | 0 | 0 | 0 | 0 | 4 | 2 | 3 | 3 | 7 | 1 | 1 | 4 | 3 | 7 | 4 | 2 | 10 | 0 | 2 |
| Moesin OS=Rattus norvegicus GN=Msn PE=1 SV=3 | MOES_RAT | 68 kDa | 8 | 7 | 14 | 11 | 13 | 8 | 9 | 7 | 8 | 5 | 16 | 6 | 4 | 5 | 1 | 0 | 14 | 6 | 13 | 10 |
| Transforming protein RhoA OS=Rattus norvegicus GN=Rhoa PE=1 SV=1 | RHOA_RAT | 22 kDa | 4 | 2 | 7 | 3 | 3 | 2 | 3 | 1 | 0 | 4 | 5 | 1 | 3 | 1 | 1 | 1 | 4 | 3 | 3 | 3 |
| Secreted phosphoprotein 24 OS=Rattus norvegicus GN=Spp2 PE=1 SV=2 | SPP24_RAT | 23 kDa | 3 | 4 | 2 | 3 | 1 | 2 | 3 | 3 | 2 | 5 | 2 | 2 | 3 | 3 | 3 | 3 | 2 | 4 | 2 | 2 |
| Ribonuclease UK114 OS=Rattus norvegicus GN=Hrsp12 PE=1 SV=3 | UK114_RAT | 14 kDa | 1 | 4 | 2 | 2 | 3 | 2 | 3 | 1 | 2 | 3 | 3 | 1 | 4 | 4 | 4 | 4 | 5 | 3 | 3 | 2 |
| Oncomodulin OS=Rattus norvegicus GN=Ocm PE=1 SV=2 | ONCO_RAT | 12 kDa | 1 | 1 | 0 | 2 | 2 | 3 | 3 | 1 | 4 | 5 | 4 | 0 | 1 | 4 | 3 | 2 | 4 | 12 | 1 | 0 |
| Plasma protease C1 inhibitor OS=Rattus norvegicus GN=Serping1 PE=2 SV=1 | IC1_RAT | 56 kDa | 0 | 0 | 0 | 0 | 1 | 1 | 3 | 1 | 0 | 1 | 1 | 1 | 8 | 3 | 8 | 9 | 2 | 1 | 3 | 8 |
| IST1 homolog OS=Rattus norvegicus GN=Ist1 PE=2 SV=1 | IST1_RAT | 40 kDa | 3 | 3 | 5 | 4 | 4 | 3 | 5 | 2 | 1 | 2 | 4 | 2 | 0 | 2 | 1 | 0 | 4 | 1 | 3 | 3 |
| Protein NOV homolog OS=Rattus norvegicus GN=Nov PE=1 SV=1 | NOV_RAT | 39 kDa | 3 | 6 | 2 | 4 | 2 | 2 | 2 | 3 | 2 | 5 | 3 | 1 | 1 | 1 | 5 | 4 | 0 | 5 | 0 | 1 |
| Cluster of EH domain-containing protein 1 OS=Rattus norvegicus GN=Ehd1 PE=1 SV=1 (EHD1_RAT) | EHD1_RAT [2] | 61 kDa | 5 | 1 | 13 | 7 | 1 | 0 | 3 | 1 | 0 | 0 | 1 | 0 | 0 | 1 | 1 | 0 | 5 | 0 | 1 | 0 |
| Cell adhesion molecule 4 OS=Rattus norvegicus GN=Cadm4 PE=1 SV=1 | CADM4_RAT | 43 kDa | 4 | 2 | 3 | 4 | 3 | 2 | 2 | 3 | 1 | 2 | 3 | 3 | 2 | 2 | 2 | 3 | 2 | 1 | 3 | 0 |
| Cathepsin Z OS=Rattus norvegicus GN=Ctsz PE=1 SV=2 | CATZ_RAT | 34 kDa | 1 | 0 | 0 | 0 | 0 | 2 | 3 | 4 | 1 | 2 | 2 | 2 | 4 | 4 | 4 | 4 | 2 | 6 | 1 | 2 |
| Chloride intracellular channel protein 1 OS=Rattus norvegicus GN=Clic1 PE=1 SV=1 | CLIC1_RAT | 27 kDa | 3 | 1 | 2 | 2 | 2 | 3 | 3 | 3 | 2 | 2 | 1 | 2 | 4 | 2 | 5 | 2 | 4 | 1 | 4 | 1 |
| Histone H2B type 1 OS=Rattus norvegicus PE=1 SV=2 | H2B1_RAT | 14 kDa | 3 | 3 | 2 | 2 | 3 | 2 | 3 | 4 | 1 | 1 | 1 | 2 | 4 | 2 | 1 | 5 | 1 | 0 | 3 | 1 |
| Histidine-rich glycoprotein OS=Rattus norvegicus GN=Hrg PE=1 SV=1 | HRG_RAT | 59 kDa | 4 | 4 | 6 | 2 | 3 | 2 | 3 | 2 | 1 | 1 | 2 | 1 | 1 | 2 | 1 | 1 | 0 | 1 | 2 | 5 |
| Inter-alpha-trypsin inhibitor heavy chain H3 OS=Rattus norvegicus GN=Itih3 PE=2 SV=1 | ITIH3_RAT | 99 kDa | 4 | 2 | 2 | 1 | 3 | 3 | 2 | 3 | 3 | 5 | 3 | 3 | 2 | 1 | 1 | 2 | 1 | 2 | 1 | 4 |
| Tumor necrosis factor receptor superfamily member 1B OS=Rattus norvegicus GN=Tnfrsf1b PE=2 SV=1 | TNR1B_RAT | 50 kDa | 0 | 0 | 0 | 0 | 1 | 2 | 1 | 2 | 2 | 5 | 1 | 0 | 2 | 9 | 5 | 5 | 2 | 5 | 2 | 3 |
| Cluster of Glutathione S-transferase alpha-1 OS=Rattus norvegicus GN=Gsta1 PE=1 SV=3 (GSTA1_RAT) | GSTA1_RAT [2] | 26 kDa | 4 | 1 | 2 | 2 | 2 | 1 | 2 | 1 | 1 | 2 | 2 | 3 | 1 | 0 | 1 | 0 | 9 | 1 | 7 | 2 |
| Acid ceramidase OS=Rattus norvegicus GN=Asah1 PE=2 SV=1 | ASAH1_RAT | 44 kDa | 2 | 2 | 2 | 3 | 2 | 3 | 3 | 1 | 2 | 2 | 4 | 3 | 2 | 3 | 2 | 1 | 2 | 1 | 2 | 2 |
| 3-mercaptopyruvate sulfurtransferase OS=Rattus norvegicus GN=Mpst PE=1 SV=3 | THTM_RAT | 33 kDa | 0 | 0 | 0 | 0 | 0 | 0 | 0 | 0 | 0 | 0 | 0 | 0 | 11 | 5 | 11 | 11 | 0 | 8 | 0 | 0 |
| Syndecan-4 OS=Rattus norvegicus GN=Sdc4 PE=1 SV=1 | SDC4_RAT | 22 kDa | 3 | 3 | 2 | 3 | 2 | 2 | 3 | 2 | 3 | 4 | 4 | 2 | 3 | 1 | 1 | 1 | 2 | 2 | 2 | 1 |
| Acidic mammalian chitinase OS=Rattus norvegicus GN=Chia PE=2 SV=1 | CHIA_RAT | 52 kDa | 1 | 1 | 0 | 0 | 1 | 1 | 0 | 1 | 1 | 1 | 0 | 1 | 2 | 2 | 3 | 3 | 9 | 5 | 7 | 7 |
| Elongation factor 1-alpha 1 OS=Rattus norvegicus GN=Eef1a1 PE=2 SV=1 | EF1A1_RAT | 50 kDa | 3 | 1 | 5 | 3 | 3 | 3 | 2 | 1 | 1 | 1 | 5 | 1 | 2 | 1 | 1 | 1 | 2 | 1 | 2 | 2 |
| Beta-microseminoprotein OS=Rattus norvegicus GN=Msmb PE=1 SV=1 | MSMB_RAT | 13 kDa | 6 | 2 | 1 | 0 | 2 | 5 | 4 | 1 | 0 | 0 | 0 | 0 | 0 | 1 | 0 | 4 | 1 | 5 | 7 | 4 |
| Rab GDP dissociation inhibitor beta OS=Rattus norvegicus GN=Gdi2 PE=1 SV=2 | GDIB_RAT | 51 kDa | 2 | 2 | 4 | 5 | 2 | 3 | 2 | 2 | 1 | 1 | 4 | 1 | 3 | 2 | 1 | 0 | 2 | 1 | 3 | 3 |
| Coatomer subunit delta OS=Rattus norvegicus GN=Arcn1 PE=2 SV=1 | COPD_RAT | 57 kDa | 1 | 2 | 2 | 1 | 3 | 2 | 0 | 2 | 1 | 1 | 0 | 1 | 1 | 2 | 1 | 3 | 1 | 0 | 1 | 1 |
| Phosphoglycerate mutase 1 OS=Rattus norvegicus GN=Pgam1 PE=1 SV=4 | PGAM1_RAT | 29 kDa | 1 | 1 | 2 | 3 | 2 | 1 | 2 | 1 | 0 | 1 | 2 | 1 | 3 | 2 | 8 | 4 | 1 | 1 | 2 | 2 |
| Glucose-6-phosphate isomerase OS=Rattus norvegicus GN=Gpi PE=1 SV=1 | G6PI_RAT | 63 kDa | 2 | 2 | 3 | 2 | 1 | 1 | 1 | 2 | 1 | 1 | 2 | 1 | 1 | 1 | 1 | 0 | 1 | 0 | 1 | 1 |
| Pituitary tumor-transforming gene 1 protein-interacting protein OS=Rattus norvegicus GN=Pttg1ip PE=2 SV=1 | PTTG_RAT | 20 kDa | 3 | 3 | 3 | 3 | 2 | 1 | 3 | 2 | 3 | 3 | 5 | 3 | 0 | 0 | 1 | 0 | 0 | 0 | 1 | 1 |
| Neuroblastoma suppressor of tumorigenicity 1 OS=Rattus norvegicus GN=Nbl1 PE=2 SV=1 | NBL1_RAT | 19 kDa | 1 | 1 | 2 | 3 | 2 | 2 | 3 | 3 | 2 | 2 | 2 | 2 | 3 | 3 | 4 | 2 | 0 | 3 | 2 | 1 |
| Parvalbumin alpha OS=Rattus norvegicus GN=Pvalb PE=1 SV=2 | PRVA_RAT | 12 kDa | 0 | 1 | 0 | 1 | 2 | 2 | 3 | 4 | 3 | 2 | 1 | 2 | 3 | 2 | 2 | 2 | 4 | 3 | 3 | 3 |
| CD320 antigen OS=Rattus norvegicus GN=Cd320 PE=2 SV=1 | CD320_RAT | 28 kDa | 1 | 4 | 3 | 0 | 2 | 0 | 4 | 3 | 2 | 3 | 2 | 1 | 4 | 0 | 1 | 2 | 1 | 1 | 1 | 0 |
| Reticulon-4 receptor-like 2 OS=Rattus norvegicus GN=Rtn4rl2 PE=1 SV=1 | R4RL2_RAT | 46 kDa | 2 | 4 | 2 | 2 | 1 | 2 | 1 | 1 | 1 | 3 | 1 | 1 | 3 | 3 | 4 | 5 | 1 | 1 | 3 | 1 |
| Platelet-derived growth factor receptor alpha OS=Rattus norvegicus GN=Pdgfra PE=1 SV=2 | PGFRA_RAT | 123 kDa | 2 | 2 | 2 | 4 | 3 | 3 | 3 | 3 | 2 | 2 | 4 | 1 | 2 | 0 | 2 | 2 | 1 | 1 | 1 | 1 |
| Apolipoprotein A-IV OS=Rattus norvegicus GN=Apoa4 PE=1 SV=2 | APOA4_RAT | 44 kDa | 4 | 7 | 6 | 5 | 0 | 3 | 3 | 2 | 0 | 0 | 0 | 0 | 0 | 0 | 0 | 0 | 0 | 0 | 0 | 3 |
| Fatty acid-binding protein, liver OS=Rattus norvegicus GN=Fabp1 PE=1 SV=1 | FABPL_RAT | 14 kDa | 0 | 0 | 0 | 0 | 0 | 0 | 0 | 0 | 0 | 0 | 0 | 0 | 9 | 1 | 14 | 11 | 0 | 0 | 0 | 0 |
| Protein S100-A9 OS=Rattus norvegicus GN=S100a9 PE=1 SV=3 | S10A9_RAT | 13 kDa | 2 | 6 | 1 | 3 | 1 | 2 | 3 | 5 | 1 | 0 | 1 | 2 | 0 | 0 | 0 | 0 | 2 | 0 | 7 | 1 |
| Histone H4 OS=Rattus norvegicus GN=Hist1h4b PE=1 SV=2 | H4_RAT | 11 kDa | 3 | 4 | 1 | 3 | 1 | 1 | 4 | 5 | 0 | 1 | 1 | 0 | 1 | 5 | 2 | 2 | 0 | 0 | 5 | 1 |
| Eosinophil cationic protein OS=Rattus norvegicus GN=Rnase3 PE=2 SV=1 | ECP_RAT | 18 kDa | 0 | 1 | 0 | 1 | 0 | 0 | 1 | 2 | 0 | 0 | 0 | 1 | 1 | 1 | 3 | 3 | 0 | 0 | 1 | 3 |
| T-cell immunoglobulin and mucin domain-containing protein 2 OS=Rattus norvegicus GN=Timd2 PE=2 SV=1 | TIMD2_RAT | 39 kDa | 2 | 2 | 2 | 2 | 3 | 2 | 2 | 2 | 5 | 3 | 3 | 3 | 1 | 2 | 1 | 1 | 1 | 2 | 1 | 1 |
| Fatty acid-binding protein, brain OS=Rattus norvegicus GN=Fabp7 PE=1 SV=2 | FABP7_RAT | 15 kDa | 0 | 0 | 0 | 0 | 0 | 0 | 0 | 0 | 0 | 1 | 0 | 0 | 10 | 7 | 12 | 10 | 0 | 0 | 0 | 0 |
| Ester hydrolase C11orf54 homolog OS=Rattus norvegicus PE=1 SV=1 | CK054_RAT | 35 kDa | 2 | 1 | 4 | 2 | 1 | 1 | 3 | 1 | 1 | 2 | 3 | 0 | 0 | 1 | 0 | 0 | 2 | 0 | 3 | 1 |
| Coagulation factor XII OS=Rattus norvegicus GN=F12 PE=2 SV=1 | FA12_RAT | 66 kDa | 5 | 4 | 5 | 5 | 2 | 2 | 2 | 2 | 4 | 2 | 3 | 1 | 0 | 0 | 1 | 0 | 0 | 0 | 0 | 0 |
| TGF-beta receptor type-2 OS=Rattus norvegicus GN=Tgfbr2 PE=1 SV=1 | TGFR2_RAT | 64 kDa | 1 | 1 | 1 | 1 | 1 | 0 | 1 | 1 | 0 | 0 | 1 | 1 | 1 | 0 | 1 | 0 | 2 | 4 | 2 | 1 |
| UPF0587 protein C1orf123 homolog OS=Rattus norvegicus PE=2 SV=1 | CA123_RAT | 18 kDa | 0 | 0 | 0 | 0 | 0 | 0 | 0 | 0 | 0 | 0 | 0 | 0 | 5 | 4 | 6 | 8 | 0 | 8 | 0 | 2 |
| Fibrinogen gamma chain OS=Rattus norvegicus GN=Fgg PE=1 SV=3 | FIBG_RAT | 51 kDa | 0 | 1 | 0 | 0 | 0 | 0 | 0 | 0 | 0 | 0 | 0 | 0 | 0 | 0 | 0 | 0 | 0 | 0 | 8 | 25 |
| Prostaglandin reductase 2 OS=Rattus norvegicus GN=Ptgr2 PE=2 SV=2 | PTGR2_RAT | 38 kDa | 0 | 0 | 1 | 0 | 0 | 0 | 1 | 0 | 0 | 0 | 0 | 0 | 10 | 3 | 13 | 8 | 0 | 1 | 0 | 0 |
| TGF-beta receptor type-1 OS=Rattus norvegicus GN=Tgfbr1 PE=1 SV=1 | TGFR1_RAT | 56 kDa | 2 | 4 | 2 | 2 | 1 | 3 | 2 | 4 | 3 | 1 | 2 | 0 | 2 | 3 | 1 | 2 | 0 | 1 | 2 | 1 |
| Choline transporter-like protein 4 OS=Rattus norvegicus GN=Slc44a4 PE=2 SV=1 | CTL4_RAT | 79 kDa | 3 | 2 | 4 | 3 | 0 | 2 | 3 | 1 | 0 | 2 | 3 | 1 | 1 | 1 | 0 | 0 | 1 | 0 | 1 | 1 |
| Acyl-protein thioesterase 1 OS=Rattus norvegicus GN=Lypla1 PE=1 SV=1 | LYPA1_RAT | 25 kDa | 0 | 0 | 0 | 0 | 0 | 0 | 0 | 0 | 0 | 0 | 0 | 0 | 9 | 5 | 7 | 6 | 0 | 9 | 0 | 0 |
| Malate dehydrogenase, cytoplasmic OS=Rattus norvegicus GN=Mdh1 PE=1 SV=3 | MDHC_RAT | 36 kDa | 1 | 1 | 3 | 4 | 1 | 4 | 2 | 3 | 0 | 2 | 2 | 1 | 3 | 2 | 2 | 1 | 0 | 0 | 1 | 1 |
| Coatomer subunit beta' OS=Rattus norvegicus GN=Copb2 PE=1 SV=3 | COPB2_RAT | 103 kDa | 0 | 0 | 0 | 1 | 1 | 0 | 1 | 0 | 1 | 2 | 0 | 0 | 6 | 1 | 6 | 2 | 0 | 1 | 0 | 0 |
| Leukocyte elastase inhibitor A OS=Rattus norvegicus GN=Serpinb1a PE=1 SV=1 | ILEUA_RAT | 43 kDa | 0 | 0 | 0 | 0 | 0 | 0 | 1 | 0 | 0 | 0 | 0 | 0 | 7 | 0 | 19 | 5 | 0 | 0 | 0 | 0 |
| Phosphoglycerate kinase 1 OS=Rattus norvegicus GN=Pgk1 PE=1 SV=2 | PGK1_RAT | 45 kDa | 2 | 2 | 4 | 1 | 0 | 0 | 1 | 0 | 1 | 1 | 1 | 0 | 2 | 2 | 4 | 1 | 1 | 0 | 1 | 0 |
| Solute carrier family 15 member 2 OS=Rattus norvegicus GN=Slc15a2 PE=1 SV=1 | S15A2_RAT | 81 kDa | 2 | 0 | 6 | 2 | 2 | 2 | 3 | 0 | 1 | 0 | 2 | 0 | 0 | 0 | 0 | 0 | 4 | 0 | 3 | 0 |
| Transmembrane protein 132A OS=Rattus norvegicus GN=Tmem132a PE=1 SV=1 | T132A_RAT | 111 kDa | 0 | 2 | 2 | 1 | 1 | 0 | 3 | 2 | 0 | 0 | 1 | 2 | 2 | 1 | 3 | 2 | 2 | 0 | 1 | 2 |
| Major prion protein OS=Rattus norvegicus GN=Prnp PE=1 SV=2 | PRIO_RAT | 28 kDa | 2 | 3 | 2 | 2 | 2 | 2 | 2 | 3 | 2 | 2 | 2 | 1 | 2 | 1 | 3 | 1 | 1 | 2 | 1 | 0 |
| Podocalyxin OS=Rattus norvegicus GN=Podxl PE=1 SV=2 | PODXL_RAT | 52 kDa | 4 | 2 | 2 | 2 | 4 | 3 | 3 | 1 | 2 | 2 | 1 | 0 | 0 | 0 | 0 | 0 | 1 | 0 | 0 | 1 |
| Ig gamma-1 chain C region OS=Rattus norvegicus PE=1 SV=1 | IGHG1_RAT | 36 kDa | 5 | 5 | 4 | 8 | 6 | 0 | 5 | 5 | 3 | 4 | 3 | 7 | 7 | 0 | 6 | 7 | 17 | 6 | 14 | 20 |
| L-lactate dehydrogenase B chain OS=Rattus norvegicus GN=Ldhb PE=1 SV=2 | LDHB_RAT | 37 kDa | 2 | 2 | 2 | 2 | 1 | 2 | 2 | 2 | 1 | 3 | 1 | 2 | 1 | 1 | 3 | 5 | 1 | 1 | 0 | 2 |
| Lysosomal thioesterase PPT2 OS=Rattus norvegicus GN=Ppt2 PE=2 SV=1 | PPT2_RAT | 34 kDa | 2 | 3 | 2 | 2 | 1 | 1 | 3 | 2 | 2 | 1 | 3 | 6 | 0 | 1 | 0 | 1 | 1 | 1 | 2 | 1 |
| Brain acid soluble protein 1 OS=Rattus norvegicus GN=Basp1 PE=1 SV=2 | BASP1_RAT | 22 kDa | 2 | 1 | 3 | 2 | 2 | 1 | 1 | 1 | 2 | 2 | 1 | 1 | 2 | 1 | 1 | 1 | 4 | 1 | 2 | 1 |
| Regenerating islet-derived protein 3-beta OS=Rattus norvegicus GN=Reg3b PE=1 SV=1 | REG3B_RAT | 20 kDa | 2 | 2 | 0 | 2 | 2 | 1 | 3 | 0 | 2 | 3 | 3 | 1 | 1 | 0 | 1 | 1 | 2 | 6 | 1 | 1 |
| Coxsackievirus and adenovirus receptor homolog OS=Rattus norvegicus GN=Cxadr PE=1 SV=2 | CXAR_RAT | 40 kDa | 0 | 2 | 2 | 1 | 2 | 1 | 2 | 2 | 2 | 1 | 1 | 1 | 2 | 0 | 2 | 2 | 0 | 2 | 0 | 0 |
| Filamin-C OS=Rattus norvegicus GN=Flnc PE=1 SV=1 | FLNC_RAT | 291 kDa | 1 | 1 | 0 | 1 | 1 | 1 | 1 | 0 | 0 | 1 | 1 | 0 | 2 | 0 | 2 | 0 | 0 | 0 | 1 | 0 |
| Enolase-phosphatase E1 OS=Rattus norvegicus GN=Enoph1 PE=2 SV=1 | ENOPH_RAT | 29 kDa | 0 | 0 | 0 | 0 | 0 | 0 | 0 | 0 | 0 | 2 | 1 | 1 | 7 | 3 | 7 | 4 | 0 | 4 | 0 | 0 |
| N-acetylgalactosamine-6-sulfatase OS=Rattus norvegicus GN=Galns PE=1 SV=1 | GALNS_RAT | 58 kDa | 3 | 2 | 6 | 4 | 0 | 1 | 2 | 0 | 1 | 2 | 2 | 0 | 1 | 0 | 1 | 0 | 0 | 0 | 1 | 0 |
| Galectin-1 OS=Rattus norvegicus GN=Lgals1 PE=1 SV=2 | LEG1_RAT | 15 kDa | 0 | 0 | 0 | 0 | 0 | 0 | 0 | 0 | 0 | 0 | 0 | 0 | 11 | 2 | 10 | 5 | 0 | 4 | 0 | 0 |
| Nucleoside diphosphate kinase B OS=Rattus norvegicus GN=Nme2 PE=1 SV=1 | NDKB_RAT | 17 kDa | 2 | 2 | 4 | 3 | 2 | 2 | 1 | 2 | 1 | 2 | 1 | 1 | 0 | 0 | 0 | 0 | 0 | 1 | 1 | 2 |
| Protein S100-A8 OS=Rattus norvegicus GN=S100a8 PE=1 SV=3 | S10A8_RAT | 10 kDa | 4 | 3 | 2 | 3 | 2 | 3 | 3 | 5 | 1 | 0 | 1 | 1 | 0 | 0 | 0 | 0 | 0 | 0 | 5 | 0 |
| Leucine-rich repeat-containing protein 15 OS=Rattus norvegicus GN=Lrrc15 PE=2 SV=1 | LRC15_RAT | 64 kDa | 2 | 5 | 3 | 1 | 2 | 2 | 3 | 3 | 1 | 1 | 1 | 0 | 0 | 0 | 0 | 0 | 0 | 0 | 2 | 3 |
| Profilin-1 OS=Rattus norvegicus GN=Pfn1 PE=1 SV=2 | PROF1_RAT | 15 kDa | 1 | 0 | 2 | 1 | 1 | 0 | 2 | 1 | 0 | 0 | 2 | 0 | 3 | 2 | 2 | 2 | 2 | 0 | 4 | 3 |
| Thy-1 membrane glycoprotein OS=Rattus norvegicus GN=Thy1 PE=1 SV=1 | THY1_RAT | 18 kDa | 3 | 0 | 1 | 1 | 1 | 3 | 2 | 3 | 4 | 1 | 2 | 3 | 2 | 1 | 3 | 2 | 0 | 1 | 0 | 0 |
| Inactive 2'-5'-oligoadenylate synthase 1B OS=Rattus norvegicus GN=Oas1b PE=2 SV=1 | OAS1B_RAT | 44 kDa | 3 | 3 | 1 | 2 | 3 | 2 | 3 | 1 | 0 | 1 | 2 | 1 | 1 | 1 | 1 | 1 | 1 | 2 | 1 | 1 |
| Glutathione S-transferase Mu 2 OS=Rattus norvegicus GN=Gstm2 PE=1 SV=2 | GSTM2_RAT | 26 kDa | 2 | 2 | 4 | 2 | 0 | 1 | 0 | 2 | 1 | 1 | 3 | 0 | 1 | 1 | 1 | 1 | 0 | 0 | 1 | 2 |
| Zinc-alpha-2-glycoprotein OS=Rattus norvegicus GN=Azgp1 PE=2 SV=1 | ZA2G_RAT | 34 kDa | 0 | 0 | 1 | 1 | 0 | 0 | 0 | 0 | 0 | 1 | 2 | 1 | 5 | 1 | 6 | 5 | 0 | 2 | 0 | 1 |
| NKG2-D type II integral membrane protein OS=Rattus norvegicus GN=Klrk1 PE=2 SV=1 | NKG2D_RAT | 24 kDa | 0 | 0 | 0 | 0 | 1 | 3 | 2 | 1 | 2 | 2 | 0 | 4 | 4 | 2 | 2 | 0 | 4 | 0 | 1 | 0 |
| Ectonucleotide pyrophosphatase/phosphodiesterase family member 5 OS=Rattus norvegicus GN=Enpp5 PE=1 SV=2 | ENPP5_RAT | 54 kDa | 0 | 2 | 1 | 2 | 1 | 2 | 3 | 1 | 0 | 1 | 0 | 0 | 4 | 2 | 3 | 5 | 0 | 2 | 1 | 0 |
| Fibrinogen alpha chain OS=Rattus norvegicus GN=Fga PE=1 SV=3 | FIBA_RAT | 87 kDa | 0 | 3 | 0 | 0 | 0 | 1 | 1 | 0 | 0 | 0 | 0 | 0 | 1 | 1 | 2 | 2 | 0 | 2 | 4 | 7 |
| Alcohol dehydrogenase [NADP(+)] OS=Rattus norvegicus GN=Akr1a1 PE=1 SV=2 | AK1A1_RAT | 37 kDa | 2 | 2 | 3 | 2 | 1 | 2 | 2 | 1 | 2 | 1 | 2 | 1 | 0 | 1 | 1 | 0 | 1 | 0 | 1 | 3 |
| Rho GDP-dissociation inhibitor 1 OS=Rattus norvegicus GN=Arhgdia PE=1 SV=1 | GDIR1_RAT | 23 kDa | 2 | 2 | 2 | 2 | 2 | 2 | 3 | 1 | 1 | 1 | 3 | 0 | 2 | 1 | 0 | 1 | 2 | 1 | 1 | 1 |
| Furin OS=Rattus norvegicus GN=Furin PE=1 SV=1 | FURIN_RAT | 87 kDa | 2 | 2 | 2 | 4 | 1 | 2 | 1 | 1 | 1 | 0 | 1 | 0 | 0 | 1 | 0 | 0 | 0 | 4 | 2 | 1 |
| Eukaryotic translation initiation factor 6 OS=Rattus norvegicus GN=Eif6 PE=1 SV=1 | IF6_RAT | 27 kDa | 0 | 1 | 1 | 0 | 0 | 1 | 0 | 0 | 0 | 0 | 0 | 1 | 2 | 2 | 9 | 5 | 0 | 6 | 1 | 1 |
| Sialidase-1 OS=Rattus norvegicus GN=Neu1 PE=1 SV=1 | NEUR1_RAT | 45 kDa | 2 | 4 | 3 | 3 | 1 | 2 | 0 | 1 | 1 | 2 | 1 | 2 | 1 | 1 | 0 | 1 | 0 | 0 | 0 | 0 |
| Uteroglobin OS=Rattus norvegicus GN=Scgb1a1 PE=1 SV=2 | UTER_RAT | 10 kDa | 2 | 5 | 2 | 3 | 1 | 2 | 0 | 0 | 3 | 5 | 1 | 1 | 1 | 0 | 0 | 0 | 1 | 3 | 0 | 0 |
| Acid sphingomyelinase-like phosphodiesterase 3a OS=Rattus norvegicus GN=Smpdl3a PE=2 SV=1 | ASM3A_RAT | 50 kDa | 4 | 3 | 2 | 2 | 3 | 3 | 3 | 3 | 0 | 1 | 0 | 0 | 0 | 1 | 0 | 0 | 0 | 0 | 2 | 0 |
| L-lactate dehydrogenase A chain OS=Rattus norvegicus GN=Ldha PE=1 SV=1 | LDHA_RAT | 36 kDa | 2 | 1 | 1 | 1 | 2 | 1 | 1 | 2 | 0 | 0 | 0 | 1 | 1 | 4 | 3 | 4 | 1 | 0 | 1 | 1 |
| OX-2 membrane glycoprotein OS=Rattus norvegicus GN=Cd200 PE=2 SV=1 | OX2G_RAT | 31 kDa | 1 | 1 | 2 | 2 | 1 | 1 | 0 | 1 | 2 | 1 | 1 | 1 | 2 | 1 | 2 | 1 | 0 | 0 | 0 | 1 |
| Beta-defensin 1 OS=Rattus norvegicus GN=Defb1 PE=2 SV=1 | DEFB1_RAT | 8 kDa | 2 | 2 | 2 | 2 | 1 | 1 | 2 | 1 | 2 | 2 | 2 | 1 | 0 | 3 | 0 | 0 | 1 | 3 | 1 | 1 |
| Secreted frizzled-related protein 4 OS=Rattus norvegicus GN=Sfrp4 PE=2 SV=2 | SFRP4_RAT | 40 kDa | 2 | 3 | 2 | 3 | 4 | 2 | 2 | 1 | 1 | 0 | 2 | 1 | 1 | 0 | 0 | 0 | 0 | 1 | 1 | 0 |
| Adhesion G protein-coupled receptor L1 OS=Rattus norvegicus GN=Adgrl1 PE=1 SV=1 | AGRL1_RAT | 167 kDa | 2 | 2 | 2 | 4 | 1 | 2 | 2 | 2 | 2 | 2 | 1 | 0 | 0 | 1 | 0 | 0 | 0 | 2 | 0 | 0 |
| Cell surface glycoprotein MUC18 OS=Rattus norvegicus GN=Mcam PE=1 SV=2 | MUC18_RAT | 71 kDa | 0 | 3 | 3 | 1 | 1 | 2 | 3 | 1 | 1 | 0 | 2 | 0 | 0 | 1 | 1 | 0 | 1 | 0 | 1 | 0 |
| Metalloproteinase inhibitor 2 OS=Rattus norvegicus GN=Timp2 PE=1 SV=3 | TIMP2_RAT | 24 kDa | 2 | 3 | 2 | 3 | 1 | 0 | 1 | 2 | 0 | 1 | 1 | 0 | 0 | 0 | 0 | 0 | 0 | 0 | 0 | 0 |
| Junctional adhesion molecule C OS=Rattus norvegicus GN=Jam3 PE=1 SV=1 | JAM3_RAT | 35 kDa | 2 | 2 | 2 | 1 | 1 | 1 | 3 | 2 | 1 | 1 | 1 | 1 | 0 | 2 | 1 | 1 | 0 | 1 | 1 | 0 |
| Palmitoyl-protein thioesterase 1 OS=Rattus norvegicus GN=Ppt1 PE=1 SV=1 | PPT1_RAT | 34 kDa | 1 | 1 | 2 | 2 | 0 | 1 | 2 | 1 | 0 | 0 | 3 | 0 | 1 | 2 | 2 | 1 | 1 | 1 | 1 | 1 |
| Vomeromodulin (Fragment) OS=Rattus norvegicus PE=2 SV=1 | VOME_RAT | 11 kDa | 4 | 1 | 1 | 1 | 1 | 0 | 1 | 1 | 0 | 1 | 1 | 1 | 1 | 2 | 1 | 1 | 0 | 2 | 0 | 0 |
| 7,8-dihydro-8-oxoguanine triphosphatase OS=Rattus norvegicus GN=Nudt1 PE=1 SV=1 | 8ODP_RAT | 18 kDa | 0 | 0 | 1 | 0 | 0 | 1 | 1 | 0 | 0 | 1 | 1 | 0 | 4 | 2 | 5 | 2 | 2 | 3 | 2 | 2 |
| Quinone oxidoreductase OS=Rattus norvegicus GN=Cryz PE=2 SV=1 | QOR_RAT | 35 kDa | 2 | 0 | 3 | 1 | 5 | 1 | 3 | 0 | 1 | 3 | 1 | 0 | 0 | 0 | 0 | 0 | 4 | 0 | 1 | 1 |
| CD302 antigen OS=Rattus norvegicus GN=Cd302 PE=2 SV=1 | CD302_RAT | 25 kDa | 2 | 1 | 2 | 2 | 0 | 3 | 0 | 1 | 1 | 2 | 0 | 0 | 2 | 2 | 3 | 0 | 0 | 0 | 0 | 0 |
| Aflatoxin B1 aldehyde reductase member 3 OS=Rattus norvegicus GN=Akr7a3 PE=1 SV=2 | ARK73_RAT | 37 kDa | 1 | 1 | 1 | 2 | 1 | 1 | 2 | 0 | 2 | 2 | 0 | 1 | 0 | 0 | 0 | 0 | 1 | 0 | 4 | 1 |
| Regucalcin OS=Rattus norvegicus GN=Rgn PE=1 SV=3 | RGN_RAT | 33 kDa | 2 | 2 | 3 | 1 | 1 | 1 | 0 | 1 | 0 | 2 | 4 | 1 | 1 | 1 | 0 | 0 | 0 | 0 | 1 | 1 |
| Cell adhesion molecule 3 OS=Rattus norvegicus GN=Cadm3 PE=1 SV=1 | CADM3_RAT | 43 kDa | 0 | 0 | 0 | 0 | 0 | 0 | 1 | 0 | 0 | 0 | 0 | 0 | 5 | 2 | 8 | 5 | 0 | 1 | 0 | 0 |
| Fibrinogen beta chain OS=Rattus norvegicus GN=Fgb PE=1 SV=4 | FIBB_RAT | 54 kDa | 0 | 0 | 0 | 0 | 0 | 0 | 0 | 0 | 0 | 0 | 0 | 0 | 0 | 0 | 0 | 0 | 0 | 0 | 7 | 21 |
| BMP and activin membrane-bound inhibitor homolog OS=Rattus norvegicus GN=Bambi PE=2 SV=1 | BAMBI_RAT | 29 kDa | 1 | 3 | 2 | 2 | 2 | 2 | 3 | 1 | 2 | 1 | 1 | 0 | 1 | 0 | 0 | 0 | 1 | 1 | 1 | 0 |
| Vitamin K-dependent protein S OS=Rattus norvegicus GN=Pros1 PE=2 SV=1 | PROS_RAT | 75 kDa | 1 | 2 | 2 | 2 | 1 | 1 | 1 | 1 | 0 | 1 | 2 | 0 | 1 | 1 | 1 | 0 | 1 | 1 | 1 | 1 |
| Cluster of Hemoglobin subunit beta-1 OS=Rattus norvegicus GN=Hbb PE=1 SV=3 (HBB1_RAT) | HBB1_RAT [2] | 16 kDa | 0 | 0 | 4 | 1 | 0 | 9 | 0 | 0 | 0 | 0 | 0 | 0 | 0 | 0 | 1 | 0 | 0 | 0 | 8 | 2 |
| Fructose-bisphosphate aldolase A OS=Rattus norvegicus GN=Aldoa PE=1 SV=2 | ALDOA_RAT | 39 kDa | 1 | 0 | 3 | 1 | 0 | 1 | 0 | 1 | 0 | 1 | 0 | 0 | 0 | 4 | 3 | 5 | 1 | 1 | 1 | 3 |
| Complement C1s subcomponent OS=Rattus norvegicus GN=C1s PE=2 SV=2 | C1S_RAT | 77 kDa | 0 | 0 | 0 | 0 | 0 | 0 | 0 | 0 | 0 | 0 | 0 | 0 | 7 | 2 | 8 | 4 | 0 | 1 | 0 | 0 |
| Beta-hexosaminidase subunit alpha OS=Rattus norvegicus GN=Hexa PE=2 SV=1 | HEXA_RAT | 61 kDa | 1 | 1 | 6 | 3 | 0 | 1 | 2 | 2 | 1 | 0 | 1 | 1 | 2 | 0 | 0 | 1 | 0 | 0 | 1 | 0 |
| Frizzled-8 OS=Rattus norvegicus GN=Fzd8 PE=2 SV=1 | FZD8_RAT | 73 kDa | 1 | 2 | 2 | 1 | 0 | 2 | 0 | 1 | 0 | 2 | 1 | 0 | 0 | 4 | 1 | 0 | 2 | 4 | 0 | 0 |
| Tyrosine-protein phosphatase non-receptor type substrate 1 OS=Rattus norvegicus GN=Sirpa PE=1 SV=1 | SHPS1_RAT | 56 kDa | 1 | 1 | 1 | 2 | 2 | 1 | 3 | 1 | 2 | 0 | 1 | 0 | 1 | 1 | 2 | 1 | 2 | 0 | 1 | 0 |
| Peptidyl-glycine alpha-amidating monooxygenase OS=Rattus norvegicus GN=Pam PE=1 SV=1 | AMD_RAT | 109 kDa | 1 | 2 | 1 | 2 | 1 | 1 | 3 | 1 | 1 | 1 | 0 | 1 | 1 | 1 | 1 | 1 | 0 | 1 | 1 | 0 |
| Insulin-like growth factor-binding protein complex acid labile subunit OS=Rattus norvegicus GN=Igfals PE=1 SV=1 | ALS_RAT | 67 kDa | 2 | 1 | 0 | 2 | 4 | 3 | 5 | 2 | 1 | 1 | 0 | 0 | 0 | 0 | 0 | 0 | 0 | 0 | 0 | 2 |
| Cystathionine gamma-lyase OS=Rattus norvegicus GN=Cth PE=1 SV=2 | CGL_RAT | 44 kDa | 1 | 1 | 5 | 1 | 2 | 1 | 1 | 1 | 1 | 0 | 1 | 0 | 0 | 0 | 0 | 0 | 4 | 0 | 2 | 0 |
| Insulin-like growth factor-binding protein 1 OS=Rattus norvegicus GN=Igfbp1 PE=1 SV=2 | IBP1_RAT | 30 kDa | 0 | 0 | 0 | 0 | 0 | 0 | 0 | 0 | 0 | 0 | 0 | 0 | 2 | 1 | 6 | 1 | 0 | 10 | 0 | 0 |
| 4F2 cell-surface antigen heavy chain OS=Rattus norvegicus GN=Slc3a2 PE=1 SV=1 | 4F2_RAT | 58 kDa | 2 | 1 | 2 | 3 | 0 | 1 | 3 | 0 | 0 | 0 | 2 | 0 | 0 | 0 | 0 | 0 | 0 | 0 | 1 | 0 |
| Lysozyme C-1 OS=Rattus norvegicus GN=Lyz1 PE=1 SV=2 | LYSC1_RAT | 17 kDa | 0 | 0 | 0 | 0 | 0 | 0 | 0 | 0 | 0 | 0 | 0 | 0 | 1 | 0 | 0 | 0 | 6 | 8 | 4 | 4 |
| Acyl-CoA-binding protein OS=Rattus norvegicus GN=Dbi PE=1 SV=3 | ACBP_RAT | 10 kDa | 1 | 0 | 3 | 0 | 1 | 1 | 2 | 1 | 0 | 0 | 1 | 0 | 1 | 2 | 4 | 3 | 0 | 0 | 0 | 0 |
| Ketohexokinase OS=Rattus norvegicus GN=Khk PE=1 SV=1 | KHK_RAT | 33 kDa | 0 | 0 | 0 | 0 | 0 | 0 | 0 | 0 | 0 | 0 | 0 | 0 | 4 | 0 | 9 | 7 | 0 | 0 | 0 | 0 |
| Insulin-like growth factor-binding protein 3 OS=Rattus norvegicus GN=Igfbp3 PE=1 SV=2 | IBP3_RAT | 32 kDa | 3 | 1 | 2 | 2 | 3 | 2 | 2 | 0 | 1 | 0 | 0 | 0 | 2 | 1 | 0 | 1 | 1 | 2 | 0 | 0 |
| Synaptic vesicle membrane protein VAT-1 homolog OS=Rattus norvegicus GN=Vat1 PE=1 SV=1 | VAT1_RAT | 43 kDa | 1 | 0 | 2 | 2 | 1 | 1 | 0 | 0 | 1 | 0 | 2 | 1 | 0 | 0 | 0 | 0 | 1 | 0 | 2 | 2 |
| Histone H2A type 1-C OS=Rattus norvegicus PE=1 SV=2 | H2A1C_RAT (+5) | 14 kDa | 1 | 2 | 0 | 1 | 1 | 0 | 2 | 2 | 0 | 0 | 0 | 0 | 2 | 2 | 1 | 1 | 1 | 0 | 3 | 1 |
| Regulating synaptic membrane exocytosis protein 2 OS=Rattus norvegicus GN=Rims2 PE=1 SV=1 | RIMS2_RAT | 176 kDa | 0 | 0 | 0 | 2 | 1 | 1 | 0 | 1 | 1 | 0 | 1 | 1 | 0 | 1 | 0 | 0 | 1 | 1 | 1 | 2 |
| Thioredoxin, mitochondrial OS=Rattus norvegicus GN=Txn2 PE=2 SV=1 | THIOM_RAT | 18 kDa | 0 | 0 | 0 | 0 | 0 | 0 | 0 | 0 | 0 | 0 | 0 | 0 | 5 | 1 | 4 | 5 | 0 | 5 | 0 | 1 |
| C4b-binding protein alpha chain OS=Rattus norvegicus GN=C4bpa PE=2 SV=1 | C4BPA_RAT | 62 kDa | 0 | 0 | 0 | 1 | 0 | 1 | 1 | 0 | 0 | 1 | 1 | 0 | 4 | 2 | 5 | 3 | 1 | 1 | 0 | 1 |
| Complement factor I OS=Rattus norvegicus GN=Cfi PE=2 SV=1 | CFAI_RAT | 67 kDa | 2 | 1 | 3 | 1 | 2 | 1 | 1 | 0 | 1 | 1 | 0 | 0 | 0 | 0 | 0 | 0 | 0 | 1 | 0 | 3 |
| Bone marrow stromal antigen 2 OS=Rattus norvegicus GN=Bst2 PE=1 SV=1 | BST2_RAT | 20 kDa | 0 | 0 | 0 | 0 | 1 | 1 | 1 | 1 | 1 | 0 | 1 | 1 | 3 | 3 | 2 | 2 | 1 | 1 | 1 | 1 |
| Metallothionein-1 OS=Rattus norvegicus GN=Mt1 PE=1 SV=1 | MT1_RAT | 6 kDa | 0 | 0 | 0 | 1 | 1 | 0 | 3 | 1 | 0 | 1 | 1 | 0 | 2 | 3 | 3 | 2 | 0 | 0 | 1 | 1 |
| Latent-transforming growth factor beta-binding protein 1 OS=Rattus norvegicus GN=Ltbp1 PE=1 SV=1 | LTBP1_RAT | 187 kDa | 1 | 1 | 1 | 0 | 1 | 1 | 0 | 1 | 1 | 1 | 2 | 1 | 1 | 0 | 1 | 1 | 0 | 0 | 1 | 0 |
| Ribonuclease inhibitor OS=Rattus norvegicus GN=Rnh1 PE=1 SV=2 | RINI_RAT | 50 kDa | 0 | 0 | 0 | 0 | 0 | 0 | 0 | 0 | 0 | 0 | 0 | 0 | 5 | 1 | 5 | 3 | 0 | 2 | 0 | 0 |
| Ubiquitin-fold modifier 1 OS=Rattus norvegicus GN=Ufm1 PE=3 SV=1 | UFM1_RAT | 9 kDa | 0 | 0 | 0 | 0 | 0 | 0 | 0 | 0 | 0 | 0 | 0 | 0 | 4 | 1 | 7 | 5 | 0 | 4 | 0 | 0 |
| Sodium/glucose cotransporter 1 OS=Rattus norvegicus GN=Slc5a1 PE=2 SV=1 | SC5A1_RAT | 73 kDa | 0 | 1 | 0 | 1 | 1 | 0 | 0 | 1 | 1 | 2 | 2 | 2 | 1 | 0 | 0 | 0 | 1 | 0 | 2 | 0 |
| Peptidyl-prolyl cis-trans isomerase B OS=Rattus norvegicus GN=Ppib PE=1 SV=3 | PPIB_RAT | 24 kDa | 2 | 0 | 2 | 2 | 1 | 1 | 2 | 1 | 0 | 1 | 1 | 1 | 0 | 0 | 0 | 0 | 0 | 0 | 1 | 2 |
| Cluster of Sulfotransferase 1C2A OS=Rattus norvegicus GN=Sult1c2a PE=2 SV=2 (S1C2A_RAT) | S1C2A_RAT | 35 kDa | 1 | 0 | 2 | 3 | 1 | 0 | 0 | 0 | 0 | 0 | 1 | 0 | 0 | 0 | 0 | 0 | 1 | 0 | 1 | 0 |
| Keratin, type II cytoskeletal 1 OS=Rattus norvegicus GN=Krt1 PE=2 SV=1 | K2C1_RAT | 65 kDa | 1 | 2 | 4 | 1 | 0 | 0 | 0 | 2 | 0 | 2 | 0 | 0 | 0 | 0 | 0 | 1 | 0 | 0 | 1 | 0 |
| Retinol-binding protein 1 OS=Rattus norvegicus GN=Rbp1 PE=1 SV=2 | RET1_RAT | 16 kDa | 0 | 0 | 0 | 0 | 0 | 0 | 0 | 0 | 0 | 0 | 0 | 0 | 5 | 4 | 7 | 3 | 0 | 0 | 0 | 0 |
| Glypican-3 OS=Rattus norvegicus GN=Gpc3 PE=1 SV=1 | GPC3_RAT | 67 kDa | 1 | 1 | 2 | 0 | 3 | 2 | 3 | 2 | 0 | 0 | 1 | 0 | 0 | 0 | 0 | 0 | 0 | 0 | 1 | 0 |
| Lipoprotein lipase OS=Rattus norvegicus GN=Lpl PE=1 SV=1 | LIPL_RAT | 53 kDa | 3 | 2 | 2 | 1 | 2 | 2 | 3 | 1 | 0 | 0 | 2 | 0 | 0 | 0 | 0 | 0 | 0 | 0 | 1 | 0 |
| Fractalkine OS=Rattus norvegicus GN=Cx3cl1 PE=2 SV=3 | X3CL1_RAT | 42 kDa | 1 | 2 | 1 | 1 | 0 | 0 | 1 | 1 | 0 | 0 | 1 | 0 | 3 | 2 | 1 | 2 | 2 | 0 | 0 | 0 |
| Seminal vesicle secretory protein 4 OS=Rattus norvegicus GN=Svs4 PE=1 SV=1 | SVS4_RAT | 12 kDa | 2 | 1 | 5 | 0 | 0 | 0 | 1 | 0 | 2 | 0 | 0 | 1 | 4 | 1 | 1 | 0 | 0 | 0 | 0 | 1 |
| CD63 antigen OS=Rattus norvegicus GN=Cd63 PE=1 SV=2 | CD63_RAT | 26 kDa | 2 | 2 | 1 | 2 | 1 | 1 | 3 | 1 | 1 | 1 | 1 | 1 | 0 | 1 | 1 | 0 | 0 | 0 | 0 | 1 |
| Cytosolic non-specific dipeptidase OS=Rattus norvegicus GN=Cndp2 PE=1 SV=1 | CNDP2_RAT | 53 kDa | 1 | 0 | 4 | 3 | 0 | 1 | 3 | 1 | 0 | 1 | 2 | 0 | 0 | 0 | 0 | 0 | 0 | 0 | 2 | 0 |
| Cytochrome b5 type B OS=Rattus norvegicus GN=Cyb5b PE=1 SV=2 | CYB5B_RAT | 16 kDa | 0 | 0 | 0 | 0 | 0 | 0 | 0 | 0 | 1 | 0 | 0 | 0 | 2 | 2 | 3 | 7 | 1 | 1 | 0 | 0 |
| Glutathione synthetase OS=Rattus norvegicus GN=Gss PE=1 SV=1 | GSHB_RAT | 52 kDa | 1 | 0 | 2 | 3 | 1 | 1 | 1 | 0 | 0 | 6 | 1 | 0 | 0 | 1 | 0 | 0 | 0 | 0 | 1 | 0 |
| Oncoprotein-induced transcript 3 protein OS=Rattus norvegicus GN=Oit3 PE=2 SV=1 | OIT3_RAT | 60 kDa | 0 | 0 | 0 | 1 | 0 | 0 | 0 | 0 | 0 | 0 | 1 | 0 | 5 | 2 | 2 | 4 | 0 | 0 | 1 | 0 |
| Tyrosine-protein kinase receptor TYRO3 OS=Rattus norvegicus GN=Tyro3 PE=1 SV=1 | TYRO3_RAT | 96 kDa | 2 | 2 | 2 | 1 | 2 | 1 | 1 | 2 | 0 | 0 | 1 | 0 | 0 | 0 | 0 | 0 | 0 | 0 | 0 | 0 |
| Integral membrane protein 2B OS=Rattus norvegicus GN=Itm2b PE=2 SV=1 | ITM2B_RAT | 30 kDa | 2 | 1 | 1 | 2 | 3 | 1 | 1 | 1 | 1 | 1 | 0 | 0 | 0 | 0 | 0 | 0 | 0 | 0 | 0 | 1 |
| Solute carrier organic anion transporter family member 1A1 OS=Rattus norvegicus GN=Slco1a1 PE=1 SV=1 | SO1A1_RAT | 74 kDa | 2 | 0 | 2 | 1 | 3 | 0 | 1 | 1 | 2 | 0 | 1 | 0 | 0 | 0 | 0 | 0 | 1 | 0 | 1 | 0 |
| Amyloid-like protein 2 OS=Rattus norvegicus GN=Aplp2 PE=1 SV=2 | APLP2_RAT | 87 kDa | 1 | 1 | 2 | 1 | 3 | 1 | 1 | 2 | 2 | 0 | 1 | 0 | 1 | 0 | 1 | 1 | 0 | 0 | 0 | 0 |
| Ectonucleotide pyrophosphatase/phosphodiesterase family member 6 OS=Rattus norvegicus GN=Enpp6 PE=1 SV=1 | ENPP6_RAT | 51 kDa | 1 | 1 | 4 | 1 | 2 | 1 | 4 | 0 | 1 | 0 | 0 | 0 | 0 | 0 | 0 | 0 | 0 | 0 | 0 | 0 |
| Adenosylhomocysteinase OS=Rattus norvegicus GN=Ahcy PE=1 SV=3 | SAHH_RAT | 48 kDa | 1 | 1 | 3 | 1 | 1 | 1 | 0 | 0 | 0 | 0 | 1 | 0 | 0 | 0 | 1 | 0 | 0 | 0 | 0 | 0 |
| Collagen triple helix repeat-containing protein 1 OS=Rattus norvegicus GN=Cthrc1 PE=1 SV=1 | CTHR1_RAT | 26 kDa | 3 | 3 | 2 | 0 | 2 | 0 | 0 | 1 | 2 | 2 | 1 | 0 | 1 | 0 | 0 | 0 | 0 | 0 | 0 | 0 |
| Endothelial protein C receptor OS=Rattus norvegicus GN=Procr PE=2 SV=1 | EPCR_RAT | 27 kDa | 0 | 0 | 2 | 0 | 0 | 1 | 2 | 1 | 0 | 0 | 1 | 0 | 1 | 4 | 0 | 1 | 1 | 1 | 2 | 1 |
| Neural cell adhesion molecule L1 OS=Rattus norvegicus GN=L1cam PE=1 SV=3 | L1CAM_RAT | 141 kDa | 0 | 0 | 2 | 2 | 1 | 1 | 1 | 0 | 1 | 0 | 1 | 1 | 0 | 0 | 1 | 0 | 0 | 0 | 1 | 0 |
| Metalloproteinase inhibitor 1 OS=Rattus norvegicus GN=Timp1 PE=1 SV=2 | TIMP1_RAT | 24 kDa | 1 | 1 | 1 | 1 | 0 | 0 | 0 | 1 | 0 | 0 | 2 | 0 | 3 | 0 | 1 | 1 | 0 | 2 | 0 | 2 |
| Lactadherin OS=Rattus norvegicus GN=Mfge8 PE=2 SV=1 | MFGM_RAT | 47 kDa | 0 | 0 | 1 | 0 | 0 | 2 | 1 | 0 | 2 | 0 | 3 | 0 | 1 | 0 | 1 | 1 | 1 | 2 | 2 | 0 |
| Thioredoxin-dependent peroxide reductase, mitochondrial OS=Rattus norvegicus GN=Prdx3 PE=1 SV=2 | PRDX3_RAT | 28 kDa | 1 | 0 | 0 | 0 | 0 | 1 | 0 | 1 | 0 | 0 | 0 | 1 | 3 | 1 | 2 | 5 | 0 | 2 | 0 | 0 |
| Ectonucleoside triphosphate diphosphohydrolase 5 OS=Rattus norvegicus GN=Entpd5 PE=2 SV=1 | ENTP5_RAT | 47 kDa | 0 | 0 | 0 | 1 | 3 | 0 | 1 | 2 | 1 | 0 | 0 | 1 | 0 | 0 | 0 | 0 | 0 | 0 | 0 | 0 |
| Fructose-1,6-bisphosphatase 1 OS=Rattus norvegicus GN=Fbp1 PE=1 SV=2 | F16P1_RAT | 40 kDa | 1 | 0 | 2 | 0 | 0 | 0 | 2 | 0 | 1 | 1 | 1 | 0 | 1 | 0 | 0 | 0 | 1 | 1 | 1 | 1 |
| Golgi apparatus protein 1 OS=Rattus norvegicus GN=Glg1 PE=1 SV=1 | GSLG1_RAT | 134 kDa | 0 | 1 | 2 | 2 | 0 | 2 | 2 | 0 | 0 | 0 | 2 | 0 | 0 | 0 | 0 | 0 | 0 | 1 | 0 | 0 |
| N-acyl-aromatic-L-amino acid amidohydrolase (carboxylate-forming) OS=Rattus norvegicus GN=Acy3 PE=1 SV=1 | ACY3_RAT | 35 kDa | 0 | 0 | 2 | 0 | 0 | 1 | 1 | 0 | 0 | 0 | 2 | 1 | 1 | 0 | 1 | 0 | 1 | 0 | 1 | 0 |
| C-type lectin domain family 4 member F OS=Rattus norvegicus GN=Clec4f PE=1 SV=1 | CLC4F_RAT | 61 kDa | 1 | 0 | 1 | 0 | 1 | 2 | 2 | 2 | 1 | 2 | 2 | 1 | 0 | 0 | 0 | 0 | 1 | 0 | 1 | 0 |
| Barrier-to-autointegration factor OS=Rattus norvegicus GN=Banf1 PE=1 SV=1 | BAF_RAT | 10 kDa | 0 | 0 | 0 | 0 | 0 | 0 | 0 | 0 | 0 | 0 | 0 | 0 | 5 | 1 | 6 | 2 | 0 | 1 | 0 | 0 |
| Kin of IRRE-like protein 1 OS=Rattus norvegicus GN=Kirrel PE=1 SV=2 | KIRR1_RAT | 87 kDa | 2 | 1 | 1 | 1 | 1 | 1 | 1 | 1 | 2 | 0 | 1 | 1 | 0 | 0 | 0 | 0 | 0 | 1 | 0 | 0 |
| 60S ribosomal protein L12 OS=Rattus norvegicus GN=Rpl12 PE=2 SV=1 | RL12_RAT | 18 kDa | 0 | 0 | 0 | 0 | 0 | 0 | 0 | 0 | 0 | 0 | 0 | 0 | 4 | 1 | 7 | 4 | 0 | 0 | 0 | 0 |
| Out at first protein homolog OS=Rattus norvegicus GN=Oaf PE=2 SV=1 | OAF_RAT | 32 kDa | 1 | 1 | 2 | 1 | 1 | 2 | 2 | 0 | 1 | 1 | 0 | 0 | 1 | 0 | 1 | 1 | 0 | 0 | 0 | 0 |
| Sodium-dependent neutral amino acid transporter B(0)AT3 OS=Rattus norvegicus GN=Slc6a18 PE=2 SV=1 | S6A18_RAT | 70 kDa | 1 | 0 | 2 | 1 | 2 | 0 | 0 | 0 | 1 | 0 | 2 | 0 | 0 | 0 | 0 | 0 | 1 | 0 | 2 | 1 |
| Low-density lipoprotein receptor OS=Rattus norvegicus GN=Ldlr PE=1 SV=1 | LDLR_RAT | 97 kDa | 2 | 1 | 1 | 2 | 0 | 1 | 1 | 0 | 0 | 0 | 1 | 0 | 0 | 0 | 0 | 0 | 0 | 2 | 0 | 0 |
| Plasminogen activator inhibitor 1 OS=Rattus norvegicus GN=Serpine1 PE=2 SV=1 | PAI1_RAT | 45 kDa | 0 | 1 | 2 | 1 | 1 | 1 | 1 | 1 | 2 | 0 | 2 | 1 | 0 | 0 | 0 | 0 | 0 | 0 | 0 | 0 |
| Frizzled-2 OS=Rattus norvegicus GN=Fzd2 PE=1 SV=1 | FZD2_RAT | 64 kDa | 0 | 3 | 2 | 1 | 0 | 1 | 0 | 0 | 0 | 0 | 2 | 0 | 1 | 0 | 1 | 0 | 0 | 2 | 1 | 0 |
| Group XV phospholipase A2 OS=Rattus norvegicus GN=Pla2g15 PE=1 SV=1 | PAG15_RAT | 47 kDa | 0 | 0 | 0 | 1 | 1 | 0 | 1 | 2 | 0 | 0 | 0 | 0 | 1 | 1 | 0 | 1 | 0 | 0 | 0 | 1 |
| Src substrate cortactin OS=Rattus norvegicus GN=Cttn PE=1 SV=1 | SRC8_RAT | 57 kDa | 0 | 0 | 2 | 1 | 0 | 0 | 0 | 1 | 1 | 0 | 0 | 1 | 0 | 1 | 0 | 0 | 1 | 2 | 0 | 1 |
| Dihydrolipoyl dehydrogenase, mitochondrial OS=Rattus norvegicus GN=Dld PE=1 SV=1 | DLDH_RAT | 54 kDa | 1 | 0 | 1 | 0 | 1 | 0 | 2 | 2 | 0 | 0 | 0 | 0 | 0 | 0 | 0 | 0 | 0 | 0 | 0 | 0 |
| CD9 antigen OS=Rattus norvegicus GN=Cd9 PE=1 SV=2 | CD9_RAT | 25 kDa | 1 | 0 | 1 | 1 | 2 | 1 | 0 | 2 | 1 | 1 | 3 | 1 | 0 | 0 | 0 | 0 | 0 | 0 | 0 | 1 |
| Aldose 1-epimerase OS=Rattus norvegicus GN=Galm PE=1 SV=1 | GALM_RAT | 38 kDa | 0 | 0 | 0 | 0 | 0 | 0 | 0 | 0 | 0 | 0 | 0 | 0 | 3 | 0 | 4 | 6 | 1 | 1 | 0 | 0 |
| Hemoglobin subunit alpha-1/2 OS=Rattus norvegicus GN=Hba1 PE=1 SV=3 | HBA_RAT | 15 kDa | 0 | 0 | 2 | 0 | 0 | 4 | 0 | 0 | 0 | 0 | 1 | 0 | 0 | 1 | 0 | 0 | 0 | 0 | 4 | 0 |
| Leukemia inhibitory factor receptor OS=Rattus norvegicus GN=Lifr PE=2 SV=1 | LIFR_RAT | 122 kDa | 0 | 2 | 1 | 2 | 0 | 0 | 3 | 1 | 0 | 0 | 1 | 0 | 0 | 2 | 0 | 0 | 0 | 0 | 0 | 1 |
| Macrophage-capping protein OS=Rattus norvegicus GN=Capg PE=1 SV=1 | CAPG_RAT | 39 kDa | 0 | 0 | 1 | 1 | 0 | 0 | 1 | 0 | 0 | 0 | 0 | 0 | 3 | 0 | 2 | 0 | 1 | 0 | 1 | 2 |
| Ephrin-B1 OS=Rattus norvegicus GN=Efnb1 PE=1 SV=1 | EFNB1_RAT | 38 kDa | 1 | 0 | 0 | 0 | 1 | 1 | 0 | 0 | 2 | 2 | 0 | 3 | 0 | 1 | 2 | 1 | 0 | 1 | 0 | 0 |
| Stanniocalcin-1 OS=Rattus norvegicus GN=Stc1 PE=2 SV=1 | STC1_RAT | 28 kDa | 1 | 2 | 2 | 1 | 1 | 1 | 0 | 1 | 1 | 1 | 1 | 0 | 0 | 0 | 0 | 0 | 1 | 1 | 0 | 1 |
| Tyrosine-protein kinase Mer OS=Rattus norvegicus GN=Mertk PE=2 SV=1 | MERTK_RAT | 109 kDa | 2 | 1 | 2 | 2 | 0 | 1 | 2 | 0 | 0 | 0 | 1 | 0 | 0 | 0 | 0 | 0 | 1 | 0 | 0 | 0 |
| Beta-defensin 50 OS=Rattus norvegicus GN=Defb50 PE=3 SV=1 | DFB50_RAT | 8 kDa | 0 | 0 | 0 | 0 | 0 | 2 | 3 | 0 | 0 | 0 | 0 | 0 | 0 | 0 | 0 | 1 | 0 | 1 | 4 | 2 |
| Glutathione S-transferase theta-2 OS=Rattus norvegicus GN=Gstt2 PE=1 SV=3 | GSTT2_RAT | 27 kDa | 0 | 0 | 1 | 0 | 0 | 0 | 1 | 0 | 0 | 0 | 0 | 0 | 3 | 0 | 2 | 3 | 0 | 0 | 0 | 0 |
| CD276 antigen OS=Rattus norvegicus GN=Cd276 PE=2 SV=1 | CD276_RAT | 34 kDa | 2 | 1 | 1 | 3 | 0 | 2 | 1 | 1 | 2 | 0 | 0 | 0 | 0 | 0 | 0 | 0 | 0 | 0 | 0 | 1 |
| Syndecan-2 OS=Rattus norvegicus GN=Sdc2 PE=2 SV=2 | SDC2_RAT | 22 kDa | 0 | 1 | 0 | 2 | 2 | 1 | 1 | 1 | 1 | 1 | 0 | 0 | 0 | 1 | 0 | 0 | 4 | 0 | 0 | 0 |
| Chromodomain-helicase-DNA-binding protein 5 OS=Rattus norvegicus GN=Chd5 PE=1 SV=1 | CHD5_RAT | 222 kDa | 2 | 0 | 0 | 0 | 0 | 0 | 0 | 0 | 0 | 0 | 0 | 0 | 0 | 0 | 1 | 0 | 1 | 0 | 0 | 0 |
| ATP-binding cassette sub-family G member 2 OS=Rattus norvegicus GN=Abcg2 PE=1 SV=1 | ABCG2_RAT | 73 kDa | 1 | 0 | 2 | 1 | 0 | 0 | 1 | 0 | 0 | 0 | 1 | 0 | 0 | 0 | 0 | 0 | 0 | 0 | 2 | 0 |
| Glutamine synthetase OS=Rattus norvegicus GN=Glul PE=1 SV=3 | GLNA_RAT | 42 kDa | 1 | 0 | 2 | 2 | 1 | 1 | 2 | 0 | 0 | 0 | 1 | 0 | 0 | 0 | 0 | 0 | 0 | 0 | 0 | 0 |
| N(4)-(Beta-N-acetylglucosaminyl)-L-asparaginase OS=Rattus norvegicus GN=Aga PE=1 SV=2 | ASPG_RAT | 37 kDa | 0 | 1 | 0 | 0 | 3 | 0 | 0 | 3 | 0 | 0 | 0 | 2 | 0 | 0 | 0 | 1 | 0 | 0 | 0 | 0 |
| Mannan-binding lectin serine protease 1 OS=Rattus norvegicus GN=Masp1 PE=1 SV=2 | MASP1_RAT | 80 kDa | 0 | 1 | 1 | 0 | 0 | 1 | 0 | 0 | 1 | 3 | 0 | 1 | 0 | 0 | 4 | 0 | 0 | 0 | 0 | 0 |
| WNT1-inducible-signaling pathway protein 1 OS=Rattus norvegicus GN=Wisp1 PE=2 SV=1 | WISP1_RAT | 41 kDa | 1 | 1 | 0 | 0 | 0 | 0 | 0 | 0 | 2 | 1 | 0 | 0 | 0 | 0 | 0 | 0 | 0 | 0 | 0 | 0 |
| Probable cytosolic iron-sulfur protein assembly protein CIAO1 OS=Rattus norvegicus GN=Ciao1 PE=2 SV=1 | CIAO1_RAT | 38 kDa | 0 | 0 | 0 | 0 | 0 | 0 | 0 | 0 | 0 | 0 | 0 | 0 | 3 | 1 | 5 | 2 | 0 | 0 | 0 | 0 |
| Cytochrome b5 OS=Rattus norvegicus GN=Cyb5a PE=1 SV=2 | CYB5_RAT | 15 kDa | 0 | 0 | 0 | 0 | 0 | 0 | 0 | 0 | 0 | 0 | 0 | 0 | 2 | 0 | 4 | 4 | 0 | 0 | 0 | 0 |
| Metallothionein-2 OS=Rattus norvegicus GN=Mt2 PE=1 SV=1 | MT2_RAT | 6 kDa | 0 | 0 | 0 | 0 | 0 | 0 | 1 | 0 | 0 | 0 | 0 | 0 | 2 | 2 | 3 | 2 | 0 | 2 | 0 | 0 |
| Pyridoxal kinase OS=Rattus norvegicus GN=Pdxk PE=1 SV=1 | PDXK_RAT | 35 kDa | 1 | 1 | 1 | 0 | 1 | 1 | 0 | 0 | 0 | 0 | 1 | 0 | 1 | 1 | 2 | 2 | 0 | 0 | 0 | 0 |
| Neuropilin-1 OS=Rattus norvegicus GN=Nrp1 PE=1 SV=1 | NRP1_RAT | 103 kDa | 0 | 1 | 2 | 1 | 1 | 0 | 2 | 1 | 0 | 0 | 0 | 0 | 0 | 0 | 1 | 0 | 0 | 0 | 0 | 0 |
| Cytoplasmic aconitate hydratase OS=Rattus norvegicus GN=Aco1 PE=1 SV=1 | ACOC_RAT | 98 kDa | 0 | 0 | 2 | 2 | 2 | 1 | 1 | 0 | 0 | 0 | 0 | 0 | 0 | 0 | 0 | 0 | 0 | 0 | 2 | 1 |
| Heat shock protein HSP 90-alpha OS=Rattus norvegicus GN=Hsp90aa1 PE=1 SV=3 | HS90A_RAT | 85 kDa | 0 | 0 | 2 | 1 | 1 | 0 | 0 | 1 | 0 | 0 | 0 | 0 | 0 | 0 | 1 | 1 | 0 | 0 | 0 | 1 |
| Epididymal-specific lipocalin-5 OS=Rattus norvegicus GN=Lcn5 PE=1 SV=2 | LCN5_RAT | 21 kDa | 0 | 0 | 0 | 0 | 0 | 0 | 0 | 0 | 0 | 0 | 0 | 0 | 0 | 0 | 0 | 0 | 0 | 0 | 0 | 10 |
| Latexin OS=Rattus norvegicus GN=Lxn PE=1 SV=1 | LXN_RAT | 26 kDa | 0 | 1 | 0 | 0 | 0 | 1 | 0 | 0 | 0 | 2 | 0 | 0 | 1 | 1 | 2 | 1 | 0 | 2 | 0 | 0 |
| Cytochrome c, somatic OS=Rattus norvegicus GN=Cycs PE=1 SV=2 | CYC_RAT | 12 kDa | 1 | 0 | 1 | 0 | 0 | 0 | 0 | 0 | 0 | 0 | 1 | 0 | 2 | 0 | 2 | 1 | 0 | 0 | 0 | 1 |
| Collectrin OS=Rattus norvegicus GN=Tmem27 PE=1 SV=2 | TMM27_RAT | 25 kDa | 0 | 0 | 2 | 1 | 1 | 0 | 1 | 1 | 1 | 1 | 0 | 0 | 0 | 0 | 0 | 0 | 1 | 0 | 0 | 0 |
| Myc box-dependent-interacting protein 1 OS=Rattus norvegicus GN=Bin1 PE=1 SV=1 | BIN1_RAT | 65 kDa | 0 | 0 | 0 | 0 | 0 | 0 | 0 | 0 | 0 | 0 | 0 | 0 | 1 | 0 | 1 | 0 | 0 | 4 | 0 | 0 |
| Fatty acid-binding protein, heart OS=Rattus norvegicus GN=Fabp3 PE=1 SV=2 | FABPH_RAT | 15 kDa | 0 | 0 | 1 | 0 | 0 | 1 | 1 | 0 | 0 | 0 | 0 | 0 | 0 | 2 | 2 | 3 | 0 | 0 | 0 | 0 |
| Tubulin alpha-1A chain OS=Rattus norvegicus GN=Tuba1a PE=1 SV=1 | TBA1A_RAT (+1) | 50 kDa | 1 | 1 | 2 | 0 | 0 | 0 | 1 | 0 | 0 | 0 | 0 | 0 | 0 | 2 | 0 | 0 | 0 | 0 | 0 | 0 |
| Malate dehydrogenase, mitochondrial OS=Rattus norvegicus GN=Mdh2 PE=1 SV=2 | MDHM_RAT | 36 kDa | 0 | 1 | 1 | 0 | 0 | 0 | 0 | 0 | 0 | 0 | 0 | 0 | 1 | 2 | 0 | 2 | 0 | 1 | 0 | 0 |
| Ectonucleotide pyrophosphatase/phosphodiesterase family member 3 OS=Rattus norvegicus GN=Enpp3 PE=1 SV=2 | ENPP3_RAT | 99 kDa | 0 | 0 | 2 | 1 | 2 | 0 | 1 | 1 | 0 | 0 | 0 | 0 | 1 | 0 | 0 | 0 | 1 | 0 | 0 | 0 |
| Nucleobindin-2 OS=Rattus norvegicus GN=Nucb2 PE=1 SV=1 | NUCB2_RAT | 50 kDa | 0 | 1 | 1 | 0 | 0 | 1 | 1 | 1 | 0 | 0 | 1 | 0 | 0 | 0 | 0 | 0 | 0 | 3 | 0 | 0 |
| Tubulin beta-2A chain OS=Rattus norvegicus GN=Tubb2a PE=1 SV=1 | TBB2A_RAT (+2) | 50 kDa | 1 | 0 | 2 | 0 | 0 | 0 | 2 | 0 | 0 | 0 | 0 | 0 | 0 | 0 | 0 | 0 | 1 | 0 | 0 | 0 |
| Unconventional myosin-Ic OS=Rattus norvegicus GN=Myo1c PE=1 SV=2 | MYO1C_RAT | 120 kDa | 1 | 0 | 5 | 1 | 0 | 0 | 0 | 0 | 0 | 0 | 0 | 0 | 0 | 0 | 0 | 0 | 0 | 0 | 0 | 0 |
| Xaa-Pro dipeptidase OS=Rattus norvegicus GN=Pepd PE=2 SV=1 | PEPD_RAT | 55 kDa | 0 | 0 | 2 | 1 | 0 | 0 | 1 | 1 | 1 | 0 | 1 | 0 | 0 | 0 | 0 | 0 | 0 | 0 | 1 | 0 |
| Chloride intracellular channel protein 4 OS=Rattus norvegicus GN=Clic4 PE=1 SV=3 | CLIC4_RAT | 29 kDa | 0 | 0 | 2 | 1 | 1 | 0 | 0 | 0 | 0 | 1 | 1 | 0 | 0 | 0 | 0 | 0 | 1 | 0 | 0 | 0 |
| Peroxiredoxin-1 OS=Rattus norvegicus GN=Prdx1 PE=1 SV=1 | PRDX1_RAT | 22 kDa | 0 | 0 | 2 | 0 | 0 | 1 | 1 | 0 | 0 | 0 | 0 | 0 | 1 | 1 | 0 | 0 | 0 | 1 | 1 | 0 |
| Guanine nucleotide-binding protein G(I)/G(S)/G(T) subunit beta-2 OS=Rattus norvegicus GN=Gnb2 PE=1 SV=4 | GBB2_RAT | 37 kDa | 1 | 0 | 2 | 1 | 0 | 1 | 0 | 0 | 0 | 0 | 0 | 0 | 0 | 0 | 0 | 0 | 1 | 0 | 1 | 1 |
| Galectin-9 OS=Rattus norvegicus GN=Lgals9 PE=2 SV=2 | LEG9_RAT | 40 kDa | 0 | 0 | 2 | 0 | 0 | 3 | 5 | 3 | 0 | 5 | 3 | 4 | 7 | 7 | 7 | 6 | 9 | 9 | 5 | 5 |
| Growth/differentiation factor 15 OS=Rattus norvegicus GN=Gdf15 PE=3 SV=1 | GDF15_RAT | 33 kDa | 1 | 1 | 0 | 0 | 1 | 1 | 1 | 0 | 0 | 0 | 1 | 1 | 2 | 0 | 0 | 0 | 0 | 0 | 0 | 0 |
| Keratin, type II cytoskeletal 5 OS=Rattus norvegicus GN=Krt5 PE=1 SV=1 | K2C5_RAT | 62 kDa | 0 | 1 | 0 | 0 | 0 | 0 | 0 | 0 | 0 | 2 | 1 | 1 | 0 | 0 | 1 | 1 | 0 | 1 | 2 | 1 |
| Voltage-dependent anion-selective channel protein 1 OS=Rattus norvegicus GN=Vdac1 PE=1 SV=4 | VDAC1_RAT | 31 kDa | 0 | 0 | 5 | 1 | 0 | 0 | 0 | 0 | 0 | 0 | 0 | 0 | 0 | 0 | 0 | 0 | 0 | 0 | 0 | 0 |
| Gastrotropin OS=Rattus norvegicus GN=Fabp6 PE=1 SV=3 | FABP6_RAT | 15 kDa | 0 | 0 | 0 | 0 | 0 | 0 | 0 | 0 | 0 | 0 | 0 | 0 | 0 | 0 | 3 | 4 | 0 | 0 | 0 | 0 |
| Aldehyde oxidase 4 OS=Rattus norvegicus GN=Aox4 PE=2 SV=1 | AOXD_RAT | 148 kDa | 1 | 0 | 0 | 0 | 0 | 0 | 0 | 0 | 0 | 0 | 0 | 0 | 0 | 1 | 0 | 0 | 0 | 2 | 0 | 0 |
| Platelet factor 4 OS=Rattus norvegicus GN=Pf4 PE=1 SV=1 | PLF4_RAT | 11 kDa | 0 | 0 | 0 | 0 | 0 | 0 | 0 | 0 | 0 | 0 | 0 | 0 | 3 | 0 | 3 | 2 | 0 | 0 | 0 | 0 |
| Protein disulfide-isomerase A4 OS=Rattus norvegicus GN=Pdia4 PE=1 SV=2 | PDIA4_RAT | 73 kDa | 0 | 0 | 1 | 0 | 0 | 0 | 1 | 0 | 0 | 0 | 0 | 0 | 2 | 1 | 1 | 1 | 1 | 0 | 0 | 0 |
| Hydroxyacid oxidase 2 OS=Rattus norvegicus GN=Hao2 PE=1 SV=2 | HAOX2_RAT | 39 kDa | 0 | 0 | 0 | 0 | 0 | 0 | 0 | 0 | 0 | 0 | 0 | 0 | 1 | 0 | 1 | 0 | 1 | 0 | 0 | 2 |
| Annexin A2 OS=Rattus norvegicus GN=Anxa2 PE=1 SV=2 | ANXA2_RAT | 39 kDa | 0 | 0 | 2 | 1 | 0 | 0 | 2 | 0 | 0 | 0 | 0 | 0 | 0 | 0 | 0 | 0 | 0 | 0 | 0 | 0 |
| Unconventional myosin-Id OS=Rattus norvegicus GN=Myo1d PE=1 SV=3 | MYO1D_RAT | 116 kDa | 0 | 0 | 2 | 0 | 0 | 0 | 1 | 0 | 0 | 0 | 2 | 0 | 0 | 0 | 0 | 0 | 0 | 0 | 0 | 0 |
| Alpha-actinin-4 OS=Rattus norvegicus GN=Actn4 PE=1 SV=2 | ACTN4_RAT | 105 kDa | 1 | 0 | 1 | 1 | 0 | 0 | 0 | 0 | 0 | 0 | 0 | 0 | 0 | 2 | 0 | 1 | 0 | 0 | 0 | 1 |
| AP-2 complex subunit alpha-2 OS=Rattus norvegicus GN=Ap2a2 PE=1 SV=3 | AP2A2_RAT | 104 kDa | 0 | 0 | 0 | 0 | 0 | 0 | 0 | 0 | 0 | 0 | 0 | 0 | 0 | 0 | 5 | 1 | 0 | 1 | 0 | 0 |
| Phosphoserine phosphatase OS=Rattus norvegicus GN=Psph PE=2 SV=1 | SERB_RAT | 25 kDa | 0 | 0 | 0 | 0 | 0 | 0 | 0 | 0 | 0 | 0 | 0 | 0 | 1 | 0 | 4 | 2 | 0 | 0 | 0 | 0 |
| Carboxypeptidase A1 OS=Rattus norvegicus GN=Cpa1 PE=2 SV=2 | CBPA1_RAT | 47 kDa | 0 | 0 | 0 | 0 | 0 | 0 | 0 | 0 | 0 | 0 | 0 | 0 | 0 | 0 | 0 | 0 | 1 | 0 | 1 | 4 |
| Growth hormone receptor OS=Rattus norvegicus GN=Ghr PE=1 SV=1 | GHR_RAT | 71 kDa | 1 | 0 | 0 | 0 | 0 | 0 | 0 | 0 | 0 | 1 | 1 | 1 | 0 | 0 | 0 | 2 | 0 | 0 | 0 | 0 |
| Ephrin type-B receptor 6 OS=Rattus norvegicus GN=Ephb6 PE=3 SV=3 | EPHB6_RAT | 110 kDa | 0 | 0 | 0 | 0 | 0 | 0 | 0 | 0 | 0 | 0 | 0 | 0 | 1 | 0 | 1 | 2 | 0 | 0 | 0 | 0 |
| Seminal vesicle secretory protein 2 OS=Rattus norvegicus GN=Svs2 PE=1 SV=1 | SVS2_RAT | 46 kDa | 2 | 0 | 0 | 1 | 0 | 0 | 2 | 0 | 1 | 0 | 0 | 0 | 1 | 0 | 0 | 0 | 0 | 0 | 0 | 0 |
| Serine protease inhibitor Kazal-type 7 OS=Rattus norvegicus GN=Spink7 PE=3 SV=1 | ISK7_RAT | 8 kDa | 0 | 1 | 0 | 0 | 0 | 1 | 0 | 0 | 1 | 1 | 0 | 0 | 0 | 0 | 0 | 0 | 0 | 2 | 0 | 0 |
| Rab GDP dissociation inhibitor alpha OS=Rattus norvegicus GN=Gdi1 PE=1 SV=1 | GDIA_RAT | 51 kDa | 0 | 0 | 1 | 1 | 0 | 0 | 1 | 0 | 0 | 0 | 1 | 0 | 2 | 1 | 1 | 1 | 0 | 1 | 0 | 0 |
| PDZ domain-containing protein GIPC2 OS=Rattus norvegicus GN=Gipc2 PE=2 SV=1 | GIPC2_RAT | 34 kDa | 1 | 1 | 2 | 2 | 0 | 1 | 1 | 0 | 0 | 0 | 0 | 0 | 0 | 1 | 0 | 0 | 1 | 1 | 0 | 0 |
| Cluster of Keratin, type II cytoskeletal 8 OS=Rattus norvegicus GN=Krt8 PE=1 SV=3 (K2C8_RAT) | K2C8_RAT | 54 kDa | 0 | 2 | 0 | 0 | 0 | 0 | 0 | 1 | 0 | 0 | 0 | 0 | 0 | 0 | 0 | 2 | 0 | 0 | 0 | 1 |
| 3'(2'),5'-bisphosphate nucleotidase 1 OS=Rattus norvegicus GN=Bpnt1 PE=1 SV=1 | BPNT1_RAT | 33 kDa | 0 | 0 | 0 | 0 | 0 | 0 | 0 | 0 | 0 | 0 | 0 | 0 | 1 | 0 | 2 | 0 | 0 | 3 | 0 | 0 |
| Serine protease inhibitor Kazal-type 1-like OS=Rattus norvegicus GN=Spink1l PE=1 SV=2 | ISK1L_RAT | 9 kDa | 0 | 0 | 0 | 0 | 0 | 0 | 0 | 0 | 0 | 0 | 0 | 1 | 3 | 1 | 0 | 0 | 0 | 0 | 0 | 0 |
| Pyruvate kinase PKM OS=Rattus norvegicus GN=Pkm PE=1 SV=3 | KPYM_RAT | 58 kDa | 0 | 0 | 2 | 1 | 0 | 0 | 0 | 0 | 0 | 0 | 0 | 0 | 0 | 1 | 1 | 0 | 0 | 0 | 0 | 0 |
| Fibulin-5 OS=Rattus norvegicus GN=Fbln5 PE=2 SV=1 | FBLN5_RAT | 50 kDa | 0 | 0 | 0 | 0 | 0 | 0 | 0 | 0 | 0 | 0 | 0 | 0 | 2 | 0 | 2 | 1 | 0 | 1 | 0 | 0 |
| Neuronal membrane glycoprotein M6-a OS=Rattus norvegicus GN=Gpm6a PE=1 SV=1 | GPM6A_RAT | 31 kDa | 0 | 1 | 2 | 0 | 0 | 0 | 1 | 0 | 0 | 0 | 1 | 0 | 0 | 0 | 0 | 0 | 0 | 0 | 0 | 0 |
| Aflatoxin B1 aldehyde reductase member 2 OS=Rattus norvegicus GN=Akr7a2 PE=1 SV=2 | ARK72_RAT | 41 kDa | 0 | 0 | 2 | 0 | 0 | 1 | 1 | 0 | 0 | 0 | 0 | 0 | 0 | 0 | 0 | 0 | 0 | 0 | 0 | 1 |
| T-cell surface glycoprotein CD8 alpha chain OS=Rattus norvegicus GN=Cd8a PE=1 SV=1 | CD8A_RAT | 26 kDa | 0 | 0 | 0 | 0 | 0 | 0 | 0 | 0 | 0 | 0 | 0 | 0 | 2 | 2 | 1 | 1 | 0 | 0 | 0 | 0 |
| Coactosin-like protein OS=Rattus norvegicus GN=Cotl1 PE=1 SV=1 | COTL1_RAT | 16 kDa | 0 | 0 | 0 | 0 | 0 | 0 | 0 | 0 | 0 | 0 | 0 | 0 | 0 | 0 | 2 | 0 | 0 | 0 | 0 | 0 |
| Sodium/potassium-transporting ATPase subunit beta-1 OS=Rattus norvegicus GN=Atp1b1 PE=1 SV=1 | AT1B1_RAT | 35 kDa | 1 | 0 | 2 | 0 | 0 | 0 | 0 | 0 | 0 | 0 | 0 | 0 | 1 | 0 | 0 | 0 | 0 | 0 | 0 | 0 |
| Ephrin-A5 OS=Rattus norvegicus GN=Efna5 PE=1 SV=2 | EFNA5_RAT | 26 kDa | 0 | 0 | 0 | 0 | 2 | 0 | 2 | 0 | 0 | 1 | 0 | 0 | 0 | 0 | 0 | 0 | 0 | 0 | 0 | 0 |
| PDZ domain-containing protein GIPC1 OS=Rattus norvegicus GN=Gipc1 PE=1 SV=2 | GIPC1_RAT | 36 kDa | 0 | 0 | 1 | 1 | 0 | 0 | 1 | 0 | 2 | 1 | 0 | 1 | 1 | 2 | 0 | 0 | 1 | 0 | 0 | 0 |
| Annexin A5 OS=Rattus norvegicus GN=Anxa5 PE=1 SV=3 | ANXA5_RAT | 36 kDa | 0 | 0 | 2 | 0 | 0 | 0 | 1 | 0 | 0 | 0 | 0 | 0 | 0 | 1 | 0 | 0 | 0 | 0 | 0 | 0 |
| Triokinase/FMN cyclase OS=Rattus norvegicus GN=Tkfc PE=1 SV=1 | TKFC_RAT | 59 kDa | 1 | 0 | 2 | 1 | 0 | 0 | 0 | 0 | 0 | 0 | 0 | 0 | 0 | 0 | 0 | 0 | 0 | 0 | 0 | 0 |
| Solute carrier family 22 member 12 OS=Rattus norvegicus GN=Slc22a12 PE=2 SV=1 | S22AC_RAT | 60 kDa | 0 | 0 | 1 | 0 | 0 | 0 | 1 | 0 | 1 | 0 | 2 | 0 | 0 | 0 | 0 | 0 | 0 | 0 | 0 | 0 |
| Tumor necrosis factor receptor superfamily member 6 OS=Rattus norvegicus GN=Fas PE=1 SV=1 | TNR6_RAT | 37 kDa | 0 | 0 | 0 | 0 | 0 | 0 | 0 | 0 | 0 | 0 | 0 | 0 | 2 | 0 | 1 | 2 | 0 | 0 | 0 | 0 |
| Phosphotriesterase-related protein OS=Rattus norvegicus GN=Pter PE=2 SV=2 | PTER_RAT | 39 kDa | 0 | 0 | 2 | 0 | 0 | 0 | 1 | 0 | 0 | 0 | 1 | 0 | 0 | 0 | 0 | 0 | 0 | 0 | 0 | 0 |
| Mannan-binding lectin serine protease 2 OS=Rattus norvegicus GN=Masp2 PE=1 SV=2 | MASP2_RAT | 76 kDa | 0 | 0 | 0 | 0 | 0 | 0 | 0 | 0 | 0 | 0 | 0 | 0 | 0 | 0 | 2 | 1 | 0 | 0 | 0 | 0 |
| Haloacid dehalogenase-like hydrolase domain-containing protein 2 OS=Rattus norvegicus GN=Hdhd2 PE=2 SV=1 | HDHD2_RAT | 29 kDa | 0 | 0 | 0 | 0 | 0 | 0 | 0 | 0 | 0 | 0 | 0 | 0 | 1 | 0 | 2 | 1 | 0 | 0 | 0 | 0 |
| Carboxypeptidase B2 OS=Rattus norvegicus GN=Cpb2 PE=2 SV=1 | CBPB2_RAT | 49 kDa | 0 | 0 | 1 | 0 | 0 | 0 | 1 | 0 | 0 | 0 | 0 | 0 | 0 | 0 | 1 | 0 | 0 | 2 | 0 | 0 |
| Protein S100-A6 OS=Rattus norvegicus GN=S100a6 PE=1 SV=3 | S10A6_RAT | 10 kDa | 0 | 0 | 0 | 0 | 0 | 0 | 0 | 0 | 0 | 0 | 0 | 0 | 0 | 0 | 2 | 1 | 0 | 0 | 0 | 2 |
| Sodium-dependent phosphate transport protein 2A OS=Rattus norvegicus GN=Slc34a1 PE=1 SV=1 | NPT2A_RAT | 69 kDa | 1 | 0 | 2 | 0 | 0 | 0 | 0 | 0 | 0 | 0 | 0 | 0 | 0 | 0 | 0 | 0 | 0 | 0 | 0 | 0 |
| Cysteine-rich secretory protein 1 OS=Rattus norvegicus GN=Crisp1 PE=2 SV=1 | CRIS1_RAT | 28 kDa | 0 | 0 | 1 | 0 | 0 | 0 | 0 | 0 | 0 | 0 | 0 | 0 | 0 | 0 | 0 | 0 | 0 | 0 | 0 | 3 |
| Elongation factor 2 OS=Rattus norvegicus GN=Eef2 PE=1 SV=4 | EF2_RAT | 95 kDa | 0 | 0 | 2 | 1 | 0 | 0 | 0 | 0 | 0 | 0 | 0 | 0 | 0 | 0 | 0 | 0 | 0 | 0 | 0 | 0 |
| Glutathione S-transferase P OS=Rattus norvegicus GN=Gstp1 PE=1 SV=2 | GSTP1_RAT | 23 kDa | 0 | 0 | 1 | 1 | 0 | 0 | 0 | 0 | 0 | 2 | 0 | 0 | 0 | 0 | 0 | 0 | 0 | 0 | 0 | 0 |
| Glutaredoxin-1 OS=Rattus norvegicus GN=Glrx PE=3 SV=3 | GLRX1_RAT | 12 kDa | 0 | 0 | 0 | 0 | 0 | 0 | 0 | 0 | 0 | 0 | 0 | 0 | 2 | 0 | 1 | 0 | 0 | 1 | 0 | 0 |
| Creatine kinase U-type, mitochondrial OS=Rattus norvegicus GN=Ckmt1 PE=1 SV=1 | KCRU_RAT | 47 kDa | 0 | 0 | 0 | 0 | 0 | 0 | 0 | 0 | 0 | 0 | 0 | 0 | 0 | 0 | 2 | 2 | 0 | 0 | 0 | 0 |
| Lipopolysaccharide-binding protein OS=Rattus norvegicus GN=Lbp PE=2 SV=1 | LBP_RAT | 54 kDa | 0 | 0 | 0 | 0 | 0 | 0 | 0 | 0 | 0 | 0 | 0 | 0 | 2 | 0 | 1 | 1 | 0 | 0 | 0 | 0 |
| Mucin-2 (Fragment) OS=Rattus norvegicus GN=Muc2 PE=1 SV=1 | MUC2_RAT | 166 kDa | 0 | 0 | 0 | 0 | 0 | 0 | 0 | 0 | 0 | 0 | 0 | 0 | 0 | 0 | 0 | 3 | 0 | 0 | 0 | 0 |
| Cornifin-A OS=Rattus norvegicus GN=Sprr1a PE=2 SV=1 | SPR1A_RAT | 17 kDa | 0 | 0 | 0 | 0 | 0 | 0 | 0 | 0 | 0 | 0 | 0 | 0 | 0 | 0 | 0 | 1 | 0 | 0 | 0 | 2 |
| Sodium-dependent multivitamin transporter OS=Rattus norvegicus GN=Slc5a6 PE=2 SV=1 | SC5A6_RAT | 69 kDa | 0 | 0 | 2 | 1 | 0 | 0 | 0 | 0 | 0 | 0 | 0 | 0 | 0 | 0 | 0 | 0 | 0 | 0 | 0 | 0 |
| Solute carrier family 22 member 7 OS=Rattus norvegicus GN=Slc22a7 PE=1 SV=1 | S22A7_RAT | 59 kDa | 0 | 0 | 2 | 0 | 0 | 0 | 0 | 0 | 0 | 0 | 0 | 0 | 0 | 0 | 0 | 0 | 1 | 0 | 0 | 0 |
| Intestinal mucin-like protein (Fragment) OS=Rattus norvegicus PE=2 SV=1 | MUC2L_RAT | 91 kDa | 0 | 2 | 0 | 0 | 1 | 0 | 0 | 0 | 0 | 0 | 0 | 0 | 0 | 0 | 0 | 0 | 0 | 0 | 0 | 0 |
| Guanine nucleotide-binding protein G(i) subunit alpha-1 OS=Rattus norvegicus GN=Gnai1 PE=1 SV=3 | GNAI1_RAT | 40 kDa | 0 | 0 | 2 | 0 | 0 | 0 | 0 | 0 | 0 | 0 | 1 | 0 | 0 | 0 | 1 | 0 | 0 | 0 | 0 | 0 |
| Calumenin OS=Rattus norvegicus GN=Calu PE=1 SV=1 | CALU_RAT | 37 kDa | 0 | 0 | 2 | 0 | 0 | 0 | 1 | 0 | 0 | 0 | 0 | 0 | 0 | 0 | 0 | 0 | 0 | 0 | 0 | 0 |
| Dihydropteridine reductase OS=Rattus norvegicus GN=Qdpr PE=1 SV=1 | DHPR_RAT | 26 kDa | 0 | 0 | 0 | 0 | 0 | 0 | 0 | 0 | 0 | 0 | 0 | 0 | 0 | 0 | 0 | 0 | 0 | 0 | 2 | 0 |
| Seminal vesicle secretory protein 5 OS=Rattus norvegicus GN=Svs5 PE=2 SV=1 | SVS5_RAT | 14 kDa | 0 | 0 | 0 | 0 | 0 | 0 | 0 | 0 | 0 | 0 | 0 | 0 | 2 | 0 | 0 | 0 | 0 | 0 | 0 | 0 |
| Vacuolar protein sorting-associated protein 4A OS=Rattus norvegicus GN=Vps4a PE=1 SV=1 | VPS4A_RAT | 49 kDa | 0 | 0 | 2 | 1 | 0 | 0 | 0 | 0 | 0 | 0 | 0 | 0 | 0 | 0 | 0 | 0 | 2 | 0 | 0 | 0 |
