# Supplementary material for: Dynamic changes of urine proteome in a Walker 256 tumor‐bearing rat model
Source: Cancer Med. 2017 Oct 4;6(11):2713–22. doi: 10.1002/cam4.1225 (PMC5673914; doi:10.1002/cam4.1225)
Supplement: Supplementary file 2 — Table S2. Differential proteins at different tumor stages. [file CAM4-6-2713-s003.docx]

**Table S2. Differential proteins at different tumor stages.**

1. **Day0 vs. Day4**

| **Protein Description** | **Accession Number** | **Molecular Weight** | **Rat1-0** | **Rat2-0** | **Rat3-0** | **Rat4-0** | **Rat1-4** | **Rat2-4** | **Rat3-4** | **Rat4-4** | **Fold change** | **p value** |
| --- | --- | --- | --- | --- | --- | --- | --- | --- | --- | --- | --- | --- |
| Galectin-3-binding protein | LG3BP_RAT | 64 kDa | 11 | 6 | 8 | 6 | 49 | 53 | 52 | 56 | 6.77 | 0.000 |
| Complement C4 | CO4_RAT | 192 kDa | 8 | 12 | 7 | 8 | 23 | 41 | 29 | 26 | 3.40 | 0.006 |
| Beta-2-microglobulin | B2MG_RAT | 14 kDa | 7 | 7 | 6 | 7 | 23 | 20 | 24 | 20 | 3.22 | 0.001 |
| Haptoglobin | HPT_RAT | 39 kDa | 5 | 2 | 4 | 5 | 14 | 13 | 9 | 10 | 2.88 | 0.015 |
| Macrophage colony-stimulating factor 1 | CSF1_RAT | 62 kDa | 1 | 5 | 4 | 3 | 7 | 7 | 7 | 6 | 2.08 | 0.027 |
| Regenerating islet-derived protein 3-gamma | REG3G_RAT | 19 kDa | 30 | 23 | 13 | 21 | 42 | 41 | 34 | 47 | 1.89 | 0.007 |
| Alpha-1-acid glycoprotein | A1AG_RAT | 24 kDa | 14 | 14 | 13 | 16 | 24 | 27 | 22 | 30 | 1.81 | 0.002 |
| Protein ABHD14B | ABHEB_RAT | 23 kDa | 9 | 9 | 6 | 6 | 15 | 14 | 11 | 10 | 1.67 | 0.001 |
| Pancreatic alpha-amylase | AMYP_RAT | 57 kDa | 26 | 30 | 19 | 19 | 17 | 15 | 15 | 14 | 0.65 | 0.046 |
| Dipeptidyl peptidase 1 | CATC_RAT | 52 kDa | 11 | 13 | 13 | 12 | 7 | 8 | 7 | 9 | 0.63 | 0.006 |
| Coagulation factor XII | FA12_RAT | 66 kDa | 5 | 4 | 5 | 5 | 2 | 2 | 2 | 2 | 0.42 | 0.002 |
| Apolipoprotein A-IV | APOA4_RAT | 44 kDa | 4 | 7 | 6 | 5 | 0 | 3 | 3 | 2 | 0.36 | 0.001 |

1. **Day0 vs. Day6**

| **Protein Description** | **Accession Number** | **Weight Molecular** | **Rat1-0** | **Rat2-0** | **Rat3-0** | **Rat4-0** | **Rat1-6** | **Rat2-6** | **Rat3-6** | **Rat4-6** | **Fold change** | **p value** |
| --- | --- | --- | --- | --- | --- | --- | --- | --- | --- | --- | --- | --- |
| Lithostathine | LITH_RAT | 19 kDa | 0 | 0 | 0 | 0 | 4 | 7 | 4 | 2 | infinity | 0.026 |
| Haptoglobin | HPT_RAT | 39 kDa | 5 | 2 | 4 | 5 | 20 | 24 | 16 | 21 | 5.06 | 0.004 |
| Complement C4 | CO4_RAT | 192 kDa | 8 | 12 | 7 | 8 | 41 | 52 | 35 | 35 | 4.66 | 0.002 |
| Galectin-3-binding protein | LG3BP_RAT | 64 kDa | 11 | 6 | 8 | 6 | 40 | 31 | 35 | 35 | 4.55 | 0.000 |
| Beta-2-microglobulin | B2MG_RAT | 14 kDa | 7 | 7 | 6 | 7 | 30 | 29 | 28 | 31 | 4.37 | 0.000 |
| Collectin-12 | COL12_RAT | 82 kDa | 2 | 4 | 0 | 2 | 6 | 7 | 6 | 7 | 3.25 | 0.006 |
| Alpha-1-acid glycoprotein | A1AG_RAT | 24 kDa | 14 | 14 | 13 | 16 | 35 | 41 | 35 | 31 | 2.49 | 0.003 |
| Thioredoxin | THIO_RAT | 12 kDa | 4 | 3 | 5 | 6 | 10 | 15 | 10 | 9 | 2.44 | 0.044 |
| Regenerating islet-derived protein 3-gamma | REG3G_RAT | 19 kDa | 30 | 23 | 13 | 21 | 50 | 58 | 39 | 54 | 2.31 | 0.004 |
| Cluster of T-kininogen 1 | KNT1_RAT [3] | 48 kDa | 18 | 20 | 16 | 18 | 36 | 38 | 34 | 34 | 1.97 | 0.000 |
| Galectin-5 | LEG5_RAT | 16 kDa | 8 | 6 | 7 | 6 | 14 | 10 | 10 | 9 | 1.59 | 0.011 |
| Retinoid-inducible serine carboxypeptidase | RISC_RAT | 51 kDa | 12 | 7 | 8 | 11 | 14 | 14 | 15 | 17 | 1.58 | 0.019 |
| Cluster of Ig kappa chain C region, B allele | KACB_RAT | 12 kDa | 136 | 177 | 153 | 167 | 209 | 260 | 249 | 281 | 1.58 | 0.002 |
| Superoxide dismutase [Cu-Zn] | SODC_RAT | 16 kDa | 10 | 9 | 6 | 10 | 14 | 14 | 12 | 15 | 1.57 | 0.001 |
| Urinary protein 1 | UP1_RAT | 11 kDa | 63 | 46 | 30 | 53 | 88 | 67 | 68 | 78 | 1.57 | 0.005 |
| Serum albumin | ALBU_RAT | 69 kDa | 217 | 214 | 219 | 202 | 144 | 135 | 139 | 149 | 0.67 | 0.001 |
| Serine protease inhibitor A3K | SPA3K_RAT | 47 kDa | 125 | 158 | 136 | 159 | 86 | 104 | 88 | 103 | 0.66 | 0.001 |
| Dipeptidyl peptidase 1 | CATC_RAT | 52 kDa | 11 | 13 | 13 | 12 | 6 | 9 | 9 | 7 | 0.63 | 0.001 |
| Fetuin-B | FETUB_RAT | 42 kDa | 17 | 18 | 16 | 18 | 13 | 12 | 9 | 9 | 0.62 | 0.008 |
| Serine protease inhibitor A3L | SPA3L_RAT | 46 kDa | 125 | 152 | 125 | 161 | 85 | 83 | 82 | 93 | 0.61 | 0.006 |
| Attractin | ATRN_RAT | 159 kDa | 11 | 9 | 8 | 10 | 6 | 5 | 5 | 6 | 0.58 | 0.002 |
| Carboxylesterase 1C | EST1C_RAT | 60 kDa | 17 | 27 | 24 | 19 | 7 | 12 | 12 | 9 | 0.46 | 0.002 |
| C-reactive protein | CRP_RAT | 25 kDa | 11 | 11 | 11 | 14 | 6 | 3 | 7 | 4 | 0.43 | 0.016 |
| Vitamin D-binding protein | VTDB_RAT | 54 kDa | 29 | 26 | 35 | 22 | 13 | 11 | 12 | 10 | 0.41 | 0.006 |
| Complement C3 | CO3_RAT | 186 kDa | 23 | 31 | 23 | 22 | 5 | 13 | 12 | 8 | 0.38 | 0.003 |
| Ceruloplasmin | CERU_RAT | 121 kDa | 10 | 17 | 12 | 13 | 3 | 2 | 7 | 6 | 0.35 | 0.031 |
| Cubilin | CUBN_RAT | 399 kDa | 14 | 20 | 19 | 23 | 4 | 3 | 11 | 4 | 0.29 | 0.015 |
| Cluster of Alpha-1-inhibitor 3 | A1I3_RAT [2] | 164 kDa | 48 | 52 | 71 | 55 | 11 | 8 | 16 | 9 | 0.19 | 0.001 |
| Apolipoprotein A-IV | APOA4_RAT | 44 kDa | 4 | 7 | 6 | 5 | 0 | 0 | 0 | 0 | 0.00 | 0.003 |

1. **Day0 vs. Day9**

| **Protein Description** | **Accession Number** | **Weight Molecular** | **Rat1-0** | **Rat2-0** | **Rat3-0** | **Rat4-0** | **Rat1-9** | **Rat2-9** | **Rat3-9** | **Rat4-9** | **Fold change** | **p value** |
| --- | --- | --- | --- | --- | --- | --- | --- | --- | --- | --- | --- | --- |
| Apolipoprotein A-IV | APOA4_RAT | 44 kDa | 4 | 7 | 6 | 5 | 0 | 0 | 0 | 0 | 0.00 | 0.003 |
| Cluster of Alpha-1-inhibitor 3 | A1I3_RAT [2] | 164 kDa | 48 | 52 | 71 | 55 | 1 | 0 | 1 | 3 | 0.02 | 0.002 |
| Programmed cell death 6-interacting protein | PDC6I_RAT | 97 kDa | 5 | 4 | 13 | 5 | 0 | 1 | 0 | 0 | 0.04 | 0.061 |
| Apolipoprotein E | APOE_RAT | 36 kDa | 13 | 12 | 14 | 12 | 0 | 1 | 1 | 0 | 0.04 | 0.000 |
| Activin receptor type-1B | ACV1B_RAT | 57 kDa | 6 | 5 | 5 | 6 | 0 | 0 | 1 | 0 | 0.05 | 0.002 |
| Na(+)/H(+) exchange regulatory cofactor NHE-RF3 | NHRF3_RAT | 57 kDa | 13 | 8 | 27 | 13 | 1 | 1 | 1 | 0 | 0.05 | 0.037 |
| Coagulation factor XII | FA12_RAT | 66 kDa | 5 | 4 | 5 | 5 | 0 | 0 | 1 | 0 | 0.05 | 0.001 |
| Glutamate--cysteine ligase regulatory subunit | GSH0_RAT | 31 kDa | 5 | 3 | 3 | 5 | 0 | 2 | 0 | 0 | 0.13 | 0.035 |
| Growth arrest-specific protein 6 | GAS6_RAT | 75 kDa | 5 | 5 | 9 | 5 | 1 | 0 | 1 | 1 | 0.13 | 0.012 |
| Attractin | ATRN_RAT | 159 kDa | 11 | 9 | 8 | 10 | 1 | 4 | 0 | 0 | 0.13 | 0.006 |
| Aggrecan core protein | PGCA_RAT | 221 kDa | 7 | 9 | 5 | 9 | 2 | 2 | 0 | 0 | 0.13 | 0.007 |
| Corticosteroid-binding globulin | CBG_RAT | 45 kDa | 16 | 17 | 19 | 19 | 2 | 4 | 2 | 2 | 0.14 | 0.001 |
| Anthrax toxin receptor 1 | ANTR1_RAT | 62 kDa | 7 | 8 | 6 | 7 | 1 | 2 | 0 | 1 | 0.14 | infinity |
| Annexin A1 | ANXA1_RAT | 39 kDa | 5 | 8 | 7 | 7 | 1 | 1 | 2 | 0 | 0.15 | 0.005 |
| Collagen alpha-1(I) chain | CO1A1_RAT | 138 kDa | 8 | 11 | 7 | 6 | 1 | 3 | 1 | 0 | 0.16 | 0.001 |
| Neogenin (Fragment) | NEO1_RAT | 151 kDa | 4 | 7 | 11 | 3 | 1 | 2 | 1 | 0 | 0.16 | 0.050 |
| Prostaglandin F2 receptor negative regulator | FPRP_RAT | 99 kDa | 5 | 3 | 5 | 5 | 2 | 0 | 1 | 0 | 0.17 | 0.004 |
| Transthyretin | TTHY_RAT | 16 kDa | 7 | 5 | 6 | 6 | 1 | 1 | 1 | 1 | 0.17 | 0.001 |
| C-reactive protein | CRP_RAT | 25 kDa | 11 | 11 | 11 | 14 | 2 | 2 | 2 | 2 | 0.17 | 0.001 |
| Glutamate--cysteine ligase catalytic subunit | GSH1_RAT | 73 kDa | 7 | 8 | 16 | 10 | 3 | 2 | 2 | 0 | 0.17 | 0.031 |
| Follistatin-related protein 1 | FSTL1_RAT | 35 kDa | 4 | 6 | 7 | 6 | 0 | 3 | 0 | 1 | 0.17 | 0.011 |
| Alkaline phosphatase, tissue-nonspecific isozyme | PPBT_RAT | 58 kDa | 4 | 3 | 8 | 5 | 2 | 2 | 0 | 0 | 0.20 | 0.085 |
| Chondroitin sulfate proteoglycan 4 | CSPG4_RAT | 252 kDa | 14 | 14 | 15 | 16 | 2 | 7 | 2 | 3 | 0.24 | 0.004 |
| Sortilin | SORT_RAT | 91 kDa | 5 | 5 | 5 | 6 | 1 | 2 | 1 | 1 | 0.24 | 0.002 |
| Moesin | MOES_RAT | 68 kDa | 8 | 7 | 14 | 11 | 4 | 5 | 1 | 0 | 0.25 | 0.067 |
| Vitamin D-binding protein | VTDB_RAT | 54 kDa | 29 | 26 | 35 | 22 | 6 | 4 | 10 | 9 | 0.26 | 0.004 |
| Serine protease inhibitor A3L | SPA3L_RAT | 46 kDa | 125 | 152 | 125 | 161 | 32 | 51 | 39 | 34 | 0.28 | 0.001 |
| Neprilysin | NEP_RAT | 86 kDa | 16 | 16 | 23 | 20 | 6 | 10 | 3 | 2 | 0.28 | 0.026 |
| Serine protease inhibitor A3K | SPA3K_RAT | 47 kDa | 125 | 158 | 136 | 159 | 36 | 59 | 42 | 32 | 0.29 | 0.001 |
| Gastricsin | PEPC_RAT | 43 kDa | 6 | 4 | 3 | 4 | 0 | 1 | 2 | 2 | 0.29 | 0.069 |
| Transcobalamin-2 | TCO2_RAT | 47 kDa | 5 | 6 | 6 | 3 | 2 | 1 | 1 | 2 | 0.30 | 0.035 |
| Urinary protein 3 | UP3_RAT | 11 kDa | 65 | 63 | 29 | 45 | 13 | 27 | 11 | 12 | 0.31 | 0.016 |
| Calbindin | CALB1_RAT | 30 kDa | 6 | 11 | 11 | 7 | 2 | 2 | 3 | 4 | 0.31 | 0.027 |
| Carboxylesterase 1C | EST1C_RAT | 60 kDa | 17 | 27 | 24 | 19 | 5 | 8 | 9 | 9 | 0.36 | 0.006 |
| Cubilin | CUBN_RAT | 399 kDa | 14 | 20 | 19 | 23 | 5 | 9 | 8 | 6 | 0.37 | 0.006 |
| Fetuin-B | FETUB_RAT | 42 kDa | 17 | 18 | 16 | 18 | 6 | 7 | 6 | 7 | 0.38 | 0.000 |
| Gamma-glutamyltranspeptidase 1 | GGT1_RAT | 62 kDa | 19 | 17 | 25 | 19 | 13 | 10 | 6 | 5 | 0.43 | 0.033 |
| Amyloid beta A4 protein | A4_RAT | 87 kDa | 5 | 4 | 4 | 3 | 2 | 1 | 2 | 2 | 0.44 | 0.018 |
| Pro-epidermal growth factor | EGF_RAT | 124 kDa | 161 | 187 | 153 | 161 | 67 | 101 | 66 | 67 | 0.45 | 0.000 |
| Lysosomal alpha-glucosidase | LYAG_RAT | 106 kDa | 13 | 10 | 12 | 10 | 6 | 7 | 5 | 3 | 0.47 | 0.009 |
| Dipeptidyl peptidase 2 | DPP2_RAT | 55 kDa | 34 | 28 | 30 | 31 | 9 | 19 | 14 | 16 | 0.47 | 0.016 |
| Low-density lipoprotein receptor-related protein 2 | LRP2_RAT | 519 kDa | 140 | 153 | 141 | 145 | 72 | 89 | 63 | 60 | 0.49 | 0.001 |
| Cystatin-related protein 1 | 22P1_RAT | 21 kDa | 41 | 40 | 32 | 28 | 21 | 26 | 10 | 16 | 0.52 | 0.006 |
| Dipeptidyl peptidase 4 | DPP4_RAT | 88 kDa | 37 | 29 | 32 | 45 | 25 | 22 | 12 | 16 | 0.52 | 0.039 |
| Dipeptidyl peptidase 1 | CATC_RAT | 52 kDa | 11 | 13 | 13 | 12 | 5 | 10 | 5 | 6 | 0.53 | 0.011 |
| Matrix-remodeling-associated protein 8 | MXRA8_RAT | 43 kDa | 45 | 44 | 37 | 37 | 20 | 28 | 22 | 17 | 0.53 | 0.004 |
| Alpha-1-macroglobulin | A1M_RAT | 167 kDa | 141 | 116 | 121 | 129 | 80 | 78 | 65 | 57 | 0.55 | 0.004 |
| Pancreatic alpha-amylase | AMYP_RAT | 57 kDa | 26 | 30 | 19 | 19 | 14 | 16 | 12 | 11 | 0.56 | 0.008 |
| Prostatic steroid-binding protein C1 | PSC1_RAT | 13 kDa | 30 | 30 | 29 | 23 | 21 | 20 | 6 | 17 | 0.57 | 0.050 |
| Nucleobindin-1 | NUCB1_RAT | 54 kDa | 14 | 9 | 11 | 13 | 7 | 7 | 6 | 7 | 0.57 | 0.019 |
| Neutral and basic amino acid transport protein rBAT | SLC31_RAT | 79 kDa | 65 | 52 | 66 | 63 | 43 | 48 | 31 | 21 | 0.58 | 0.054 |
| Kallikrein-1 | KLK1_RAT | 29 kDa | 38 | 45 | 37 | 41 | 22 | 26 | 26 | 21 | 0.59 | 0.004 |
| Meprin A subunit alpha | MEP1A_RAT | 85 kDa | 51 | 49 | 51 | 47 | 27 | 46 | 26 | 24 | 0.62 | 0.038 |
| Fibronectin | FINC_RAT | 273 kDa | 63 | 62 | 58 | 60 | 43 | 41 | 39 | 34 | 0.65 | 0.001 |
| Clusterin | CLUS_RAT | 51 kDa | 9 | 11 | 11 | 12 | 21 | 14 | 14 | 20 | 1.60 | 0.058 |
| Alpha-1-antiproteinase | A1AT_RAT | 46 kDa | 37 | 37 | 28 | 35 | 46 | 46 | 68 | 61 | 1.61 | 0.068 |
| Hemopexin | HEMO_RAT | 51 kDa | 62 | 57 | 50 | 66 | 102 | 103 | 103 | 111 | 1.78 | 0.000 |
| Apolipoprotein M | APOM_RAT | 22 kDa | 4 | 7 | 5 | 5 | 13 | 8 | 8 | 9 | 1.81 | 0.088 |
| Phospholysine phosphohistidine inorganic pyrophosphate phosphatase | LHPP_RAT | 29 kDa | 5 | 4 | 3 | 5 | 9 | 9 | 7 | 6 | 1.82 | 0.027 |
| 6-phosphogluconolactonase | 6PGL_RAT | 27 kDa | 19 | 14 | 13 | 14 | 36 | 21 | 26 | 28 | 1.85 | 0.009 |
| Gelsolin | GELS_RAT | 86 kDa | 24 | 28 | 12 | 30 | 44 | 36 | 47 | 54 | 1.93 | 0.030 |
| Polymeric immunoglobulin receptor | PIGR_RAT | 85 kDa | 30 | 31 | 22 | 29 | 43 | 61 | 58 | 60 | 1.98 | 0.012 |
| Extracellular superoxide dismutase [Cu-Zn] | SODE_RAT | 27 kDa | 9 | 9 | 8 | 8 | 20 | 13 | 22 | 19 | 2.18 | 0.018 |
| Interleukin-1 receptor type 2 | IL1R2_RAT | 46 kDa | 2 | 1 | 3 | 2 | 5 | 4 | 4 | 5 | 2.25 | 0.015 |
| Macrophage colony-stimulating factor 1 | CSF1_RAT | 62 kDa | 1 | 5 | 4 | 3 | 8 | 10 | 6 | 6 | 2.31 | 0.031 |
| Vascular cell adhesion protein 1 | VCAM1_RAT | 81 kDa | 12 | 13 | 9 | 6 | 28 | 28 | 23 | 24 | 2.58 | 0.000 |
| Cathepsin B | CATB_RAT | 37 kDa | 9 | 13 | 9 | 11 | 24 | 32 | 33 | 33 | 2.90 | 0.002 |
| Intercellular adhesion molecule 1 | ICAM1_RAT | 60 kDa | 4 | 4 | 3 | 5 | 12 | 15 | 13 | 12 | 3.25 | 0.002 |
| Haptoglobin | HPT_RAT | 39 kDa | 5 | 2 | 4 | 5 | 20 | 14 | 10 | 9 | 3.31 | 0.036 |
| Protein ABHD14B | ABHEB_RAT | 23 kDa | 9 | 9 | 6 | 6 | 25 | 27 | 22 | 31 | 3.50 | 0.003 |
| Galectin-5 | LEG5_RAT | 16 kDa | 8 | 6 | 7 | 6 | 22 | 25 | 25 | 23 | 3.52 | 0.001 |
| Cluster of T-kininogen 1 | KNT1_RAT [3] | 48 kDa | 18 | 20 | 16 | 18 | 54 | 63 | 68 | 75 | 3.61 | 0.002 |
| Angiotensinogen | ANGT_RAT | 52 kDa | 3 | 4 | 3 | 2 | 11 | 5 | 13 | 15 | 3.67 | 0.052 |
| Cathepsin S | CATS_RAT | 37 kDa | 1 | 1 | 1 | 2 | 5 | 4 | 5 | 6 | 4.00 | 0.001 |
| 1,2-dihydroxy-3-keto-5-methylthiopentene dioxygenase | MTND_RAT | 21 kDa | 4 | 3 | 4 | 4 | 18 | 14 | 13 | 15 | 4.00 | 0.002 |
| Thioredoxin | THIO_RAT | 12 kDa | 4 | 3 | 5 | 6 | 21 | 13 | 25 | 21 | 4.44 | 0.005 |
| Nuclear transport factor 2 | NTF2_RAT | 14 kDa | 2 | 2 | 4 | 2 | 13 | 10 | 15 | 10 | 4.80 | 0.002 |
| Neutrophil collagenase | MMP8_RAT | 53 kDa | 1 | 3 | 1 | 3 | 11 | 11 | 10 | 13 | 5.63 | 0.000 |
| Superoxide dismutase [Cu-Zn] | SODC_RAT | 16 kDa | 10 | 9 | 6 | 10 | 52 | 38 | 52 | 60 | 5.77 | 0.003 |
| Peroxiredoxin-5, mitochondrial | PRDX5_RAT | 22 kDa | 2 | 1 | 5 | 2 | 16 | 7 | 23 | 12 | 5.80 | 0.019 |
| Alpha-1-acid glycoprotein | A1AG_RAT | 24 kDa | 14 | 14 | 13 | 16 | 71 | 71 | 93 | 110 | 6.05 | 0.004 |
| Complement C4 | CO4_RAT | 192 kDa | 8 | 12 | 7 | 8 | 59 | 34 | 62 | 60 | 6.14 | 0.010 |
| Phosphatidylethanolamine-binding protein 1 | PEBP1_RAT | 21 kDa | 2 | 3 | 3 | 1 | 11 | 9 | 22 | 19 | 6.78 | 0.028 |
| Beta-2-microglobulin | B2MG_RAT | 14 kDa | 7 | 7 | 6 | 7 | 62 | 45 | 59 | 59 | 8.33 | 0.001 |
| Galectin-9 | LEG9_RAT | 40 kDa | 0 | 0 | 2 | 0 | 7 | 7 | 7 | 6 | 13.50 | 0.001 |
| Cathepsin Z | CATZ_RAT | 34 kDa | 1 | 0 | 0 | 0 | 4 | 4 | 4 | 4 | 16.00 | 0.001 |
| Ribonuclease 4 | RNAS4_RAT | 17 kDa | 1 | 0 | 0 | 0 | 4 | 3 | 7 | 4 | 18.00 | 0.021 |
| Neutrophil gelatinase-associated lipocalin | NGAL_RAT | 22 kDa | 6 | 6 | 2 | 3 | 84 | 61 | 98 | 81 | 19.06 | 0.003 |
| Complement component C9 | CO9_RAT | 62 kDa | 0 | 1 | 2 | 1 | 20 | 13 | 35 | 24 | 23.00 | 0.015 |
| Glutathione S-transferase omega-1 | GSTO1_RAT | 28 kDa | 0 | 0 | 2 | 0 | 13 | 8 | 14 | 11 | 23.00 | 0.002 |
| Isopentenyl-diphosphate Delta-isomerase 1 | IDI1_RAT | 26 kDa | 0 | 0 | 0 | 2 | 14 | 12 | 17 | 20 | 31.50 | 0.002 |
| Prostaglandin reductase 2 | PTGR2_RAT | 38 kDa | 0 | 0 | 1 | 0 | 10 | 3 | 13 | 8 | 34.00 | 0.024 |
| Protein deglycase DJ-1 | PARK7_RAT | 20 kDa | 0 | 0 | 2 | 1 | 22 | 13 | 40 | 27 | 34.00 | 0.017 |
| Ig gamma-2B chain C region | IGG2B_RAT | 36 kDa | 0 | 1 | 0 | 0 | 11 | 8 | 11 | 16 | 46.00 | 0.009 |
| Fatty acid-binding protein, liver | FABPL_RAT | 14 kDa | 0 | 0 | 0 | 0 | 9 | 1 | 14 | 11 | infinity | 0.051 |
| 60S ribosomal protein L12 | RL12_RAT | 18 kDa | 0 | 0 | 0 | 0 | 4 | 1 | 7 | 4 | infinity | 0.047 |
| Carbonic anhydrase 3 | CAH3_RAT | 29 kDa | 0 | 0 | 0 | 0 | 30 | 4 | 36 | 44 | infinity | 0.046 |
| Galectin-1 | LEG1_RAT | 15 kDa | 0 | 0 | 0 | 0 | 11 | 2 | 10 | 5 | infinity | 0.046 |
| Ubiquitin-fold modifier 1 | UFM1_RAT | 9 kDa | 0 | 0 | 0 | 0 | 4 | 1 | 7 | 5 | infinity | 0.042 |
| Tumor necrosis factor receptor superfamily member 1B | TNR1B_RAT | 50 kDa | 0 | 0 | 0 | 0 | 2 | 9 | 5 | 5 | infinity | 0.035 |
| Complement C1s subcomponent | C1S_RAT | 77 kDa | 0 | 0 | 0 | 0 | 7 | 2 | 8 | 4 | infinity | 0.032 |
| Cell adhesion molecule 3 | CADM3_RAT | 43 kDa | 0 | 0 | 0 | 0 | 5 | 2 | 8 | 5 | infinity | 0.027 |
| Enolase-phosphatase E1 | ENOPH_RAT | 29 kDa | 0 | 0 | 0 | 0 | 7 | 3 | 7 | 4 | infinity | 0.015 |
| Plasma protease C1 inhibitor | IC1_RAT | 56 kDa | 0 | 0 | 0 | 0 | 8 | 3 | 8 | 9 | infinity | 0.014 |
| Retinol-binding protein 1 | RET1_RAT | 16 kDa | 0 | 0 | 0 | 0 | 5 | 4 | 7 | 3 | infinity | 0.011 |
| Lithostathine | LITH_RAT | 19 kDa | 0 | 0 | 0 | 0 | 11 | 9 | 14 | 6 | infinity | 0.010 |
| Carbonic anhydrase 1 | CAH1_RAT | 28 kDa | 0 | 0 | 0 | 0 | 33 | 15 | 35 | 29 | infinity | 0.008 |
| 3-mercaptopyruvate sulfurtransferase | THTM_RAT | 33 kDa | 0 | 0 | 0 | 0 | 11 | 5 | 11 | 11 | infinity | 0.008 |
| D-dopachrome decarboxylase | DOPD_RAT | 13 kDa | 0 | 0 | 0 | 0 | 15 | 7 | 15 | 15 | infinity | 0.007 |
| UPF0587 protein C1orf123 homolog | CA123_RAT | 18 kDa | 0 | 0 | 0 | 0 | 5 | 4 | 6 | 8 | infinity | 0.007 |
| Acyl-protein thioesterase 1 | LYPA1_RAT | 25 kDa | 0 | 0 | 0 | 0 | 9 | 5 | 7 | 6 | infinity | 0.004 |
| Fatty acid-binding protein, brain | FABP7_RAT | 15 kDa | 0 | 0 | 0 | 0 | 10 | 7 | 12 | 10 | infinity | 0.003 |

1. **Day0 vs. Day14**

| **Protein Description** | **Accession Number** | **Weight Molecular** | **Rat1-0** | **Rat2-0** | **Rat3-0** | **Rat4-0** | **Rat1-14** | **Rat2-14** | **Rat3-14** | **Rat4-14** | **Fold change** | **p value** |
| --- | --- | --- | --- | --- | --- | --- | --- | --- | --- | --- | --- | --- |
| Lysozyme C-1 | LYSC1_RAT | 17 kDa | 0 | 0 | 0 | 0 | 6 | 8 | 4 | 4 | infinity | 0.010 |
| Ig gamma-2B chain C region | IGG2B_RAT | 36 kDa | 0 | 1 | 0 | 0 | 9 | 9 | 12 | 32 | 62.00 | 0.074 |
| Galectin-9 | LEG9_RAT | 40 kDa | 0 | 0 | 2 | 0 | 9 | 9 | 5 | 5 | 14.00 | 0.023 |
| Acidic mammalian chitinase | CHIA_RAT | 52 kDa | 1 | 1 | 0 | 0 | 9 | 5 | 7 | 7 | 14.00 | 0.005 |
| Beta-2-microglobulin | B2MG_RAT | 14 kDa | 7 | 7 | 6 | 7 | 32 | 42 | 25 | 18 | 4.33 | 0.021 |
| Complement C4 | CO4_RAT | 192 kDa | 8 | 12 | 7 | 8 | 25 | 42 | 25 | 33 | 3.57 | 0.005 |
| Haptoglobin | HPT_RAT | 39 kDa | 5 | 2 | 4 | 5 | 14 | 18 | 7 | 18 | 3.56 | 0.036 |
| Galectin-5 | LEG5_RAT | 16 kDa | 8 | 6 | 7 | 6 | 22 | 32 | 14 | 15 | 3.07 | 0.046 |
| Cluster of T-kininogen 1 | KNT1_RAT [3] | 48 kDa | 18 | 20 | 16 | 18 | 38 | 87 | 28 | 65 | 3.03 | 0.063 |
| Ig gamma-2A chain C region | IGG2A_RAT | 35 kDa | 14 | 11 | 13 | 19 | 46 | 25 | 39 | 55 | 2.89 | 0.011 |
| Ig lambda-2 chain C region | LAC2_RAT | 11 kDa | 39 | 39 | 33 | 39 | 89 | 62 | 93 | 74 | 2.12 | 0.014 |
| Cluster of Ig kappa chain C region, B allele | KACB_RAT | 12 kDa | 136 | 177 | 153 | 167 | 379 | 302 | 357 | 300 | 2.11 | 0.008 |
| 6-phosphogluconolactonase | 6PGL_RAT | 27 kDa | 19 | 14 | 13 | 14 | 28 | 43 | 20 | 26 | 1.95 | 0.066 |
| Alpha-1-acid glycoprotein | A1AG_RAT | 24 kDa | 14 | 14 | 13 | 16 | 25 | 30 | 21 | 25 | 1.77 | 0.009 |
| Serine protease inhibitor A3K | SPA3K_RAT | 47 kDa | 125 | 158 | 136 | 159 | 91 | 73 | 121 | 82 | 0.63 | 0.052 |
| Low-density lipoprotein receptor-related protein 2 | LRP2_RAT | 519 kDa | 140 | 153 | 141 | 145 | 111 | 68 | 109 | 74 | 0.63 | 0.031 |
| Serine protease inhibitor A3L | SPA3L_RAT | 46 kDa | 125 | 152 | 125 | 161 | 90 | 73 | 103 | 81 | 0.62 | 0.037 |
| Nucleobindin-1 | NUCB1_RAT | 54 kDa | 14 | 9 | 11 | 13 | 6 | 5 | 8 | 7 | 0.55 | 0.018 |
| Attractin | ATRN_RAT | 159 kDa | 11 | 9 | 8 | 10 | 6 | 5 | 5 | 4 | 0.53 | 0.006 |
| Dipeptidyl peptidase 2 | DPP2_RAT | 55 kDa | 34 | 28 | 30 | 31 | 12 | 10 | 21 | 15 | 0.47 | 0.009 |
| C-reactive protein | CRP_RAT | 25 kDa | 11 | 11 | 11 | 14 | 6 | 3 | 5 | 7 | 0.45 | 0.002 |
| Dipeptidyl peptidase 1 | CATC_RAT | 52 kDa | 11 | 13 | 13 | 12 | 6 | 6 | 4 | 4 | 0.41 | 0.003 |
| Anthrax toxin receptor 1 | ANTR1_RAT | 62 kDa | 7 | 8 | 6 | 7 | 4 | 2 | 3 | 2 | 0.39 | 0.011 |
| Tripeptidyl-peptidase 1 | TPP1_RAT | 61 kDa | 3 | 3 | 6 | 4 | 1 | 2 | 1 | 2 | 0.38 | 0.063 |
| Chondroitin sulfate proteoglycan 4 | CSPG4_RAT | 252 kDa | 14 | 14 | 15 | 16 | 4 | 6 | 7 | 4 | 0.36 | 0.002 |
| Lysosomal alpha-glucosidase | LYAG_RAT | 106 kDa | 13 | 10 | 12 | 10 | 4 | 5 | 2 | 5 | 0.36 | 0.012 |
| Cubilin | CUBN_RAT | 399 kDa | 14 | 20 | 19 | 23 | 10 | 1 | 11 | 4 | 0.34 | 0.047 |
| Growth arrest-specific protein 6 | GAS6_RAT | 75 kDa | 5 | 5 | 9 | 5 | 2 | 1 | 3 | 2 | 0.33 | 0.011 |
| Alkaline phosphatase, tissue-nonspecific isozyme | PPBT_RAT | 58 kDa | 4 | 3 | 8 | 5 | 2 | 0 | 2 | 2 | 0.30 | 0.027 |
| Transcobalamin-2 | TCO2_RAT | 47 kDa | 5 | 6 | 6 | 3 | 1 | 2 | 2 | 1 | 0.30 | 0.006 |
| Aggrecan core protein | PGCA_RAT | 221 kDa | 7 | 9 | 5 | 9 | 1 | 3 | 1 | 1 | 0.20 | 0.005 |
| Prostaglandin F2 receptor negative regulator | FPRP_RAT | 99 kDa | 5 | 3 | 5 | 5 | 0 | 1 | 2 | 0 | 0.17 | 0.015 |
| Apolipoprotein A-IV | APOA4_RAT | 44 kDa | 4 | 7 | 6 | 5 | 0 | 0 | 0 | 3 | 0.14 | 0.023 |
| Collagen alpha-1(I) chain | CO1A1_RAT | 138 kDa | 8 | 11 | 7 | 6 | 1 | 1 | 1 | 0 | 0.09 | 0.005 |
| Programmed cell death 6-interacting protein | PDC6I_RAT | 97 kDa | 5 | 4 | 13 | 5 | 1 | 0 | 0 | 1 | 0.07 | 0.069 |
| Beta-glucuronidase | BGLR_RAT | 75 kDa | 33 | 9 | 10 | 16 | 1 | 0 | 0 | 3 | 0.06 | 0.059 |
| Calbindin | CALB1_RAT | 30 kDa | 6 | 11 | 11 | 7 | 0 | 0 | 0 | 0 | 0.00 | 0.007 |
| Coagulation factor XII | FA12_RAT | 66 kDa | 5 | 4 | 5 | 5 | 0 | 0 | 0 | 0 | 0.00 | 0.000 |
