# Supplementary material for: Dynamic changes of urine proteome in a Walker 256 tumor‐bearing rat model
Source: Cancer Med. 2017 Oct 4;6(11):2713–22. doi: 10.1002/cam4.1225 (PMC5673914; doi:10.1002/cam4.1225)
Supplement: Supplementary file 3 — Table S3. Cancer biomarkers annotated in the IPA database. [file CAM4-6-2713-s004.docx]

**Table S3. Cancer biomarkers annotated in the IPA database.**

| **Symbol** | **Entrez Gene Name** | **UniProt Accession** | **Human** | **Rat** | **Blood** | **Plasma/Serum** | **Urine** | **Biomarker Application(s)** | **Diseases** |
| --- | --- | --- | --- | --- | --- | --- | --- | --- | --- |
| ALPL | alkaline phosphatase, liver/bone/kidney | PPBT_RAT | √ | √ | √ | √ | √ | efficacy, safety | Cancer, Cardiovascular Disease, Connective Tissue Disorders, Dental Disease, Dermatological Diseases and Conditions, Developmental Disorder, Endocrine System Disorders, Gastrointestinal Disease, Hematological Disease, Hepatic System Disease, Hereditary Disorder, Immunological Disease, Inflammatory Disease, Inflammatory Response, Metabolic Disease, Neurological Disease, Nutritional Disease, Organismal Injury and Abnormalities, Renal and Urological Disease, Reproductive System Disease, Respiratory Disease, Skeletal and Muscular Disorders |
| ANTXR1 | anthrax toxin receptor 1 | ANTR1_RAT | √ | √ | √ |  | √ | diagnosis | Cancer, Cardiovascular Disease, Connective Tissue Disorders, Dental Disease, Dermatological Diseases and Conditions, Developmental Disorder, Gastrointestinal Disease, Hematological Disease, Hereditary Disorder, Immunological Disease, Inflammatory Disease, Neurological Disease, Ophthalmic Disease, Organismal Injury and Abnormalities, Psychological Disorders, Reproductive System Disease, Respiratory Disease, Skeletal and Muscular Disorders, Tumor Morphology |
| ANXA1 | annexin A1 | AN√A1_RAT | √ | √ | √ | √ | √ | diagnosis, prognosis, unspecified application | Cancer, Cardiovascular Disease, Connective Tissue Disorders, Dermatological Diseases and Conditions, Developmental Disorder, Endocrine System Disorders, Gastrointestinal Disease, Hematological Disease, Hepatic System Disease, Hereditary Disorder, Hypersensitivity Response, Immunological Disease, Infectious Disease, Inflammatory Disease, Inflammatory Response, Metabolic Disease, Neurological Disease, Ophthalmic Disease, Organismal Injury and Abnormalities, Psychological Disorders, Renal and Urological Disease, Reproductive System Disease, Respiratory Disease, Skeletal and Muscular Disorders, Tumor Morphology |
| APOE | apolipoprotein E | APOE_RAT | √ | √ | √ | √ | √ | diagnosis, efficacy, prognosis, unspecified application | Auditory Disease, Cancer, Cardiovascular Disease, Connective Tissue Disorders, Dermatological Diseases and Conditions, Developmental Disorder, Endocrine System Disorders, Gastrointestinal Disease, Hematological Disease, Hepatic System Disease, Hereditary Disorder, Immunological Disease, Infectious Disease, Inflammatory Disease, Inflammatory Response, Metabolic Disease, Neurological Disease, Nutritional Disease, Ophthalmic Disease, Organismal Injury and Abnormalities, Psychological Disorders, Renal and Urological Disease, Reproductive System Disease, Respiratory Disease, Skeletal and Muscular Disorders, Tumor Morphology |
| AZGP1 | alpha-2-glycoprotein 1, zinc-binding | ZA2G_RAT | √ | √ | √ | √ | √ | unspecified application | Cancer, Cardiovascular Disease, Connective Tissue Disorders, Dermatological Diseases and Conditions, Developmental Disorder, Endocrine System Disorders, Gastrointestinal Disease, Hereditary Disorder, Immunological Disease, Inflammatory Disease, Inflammatory Response, Nutritional Disease, Organismal Injury and Abnormalities, Reproductive System Disease, Respiratory Disease, Skeletal and Muscular Disorders, Tumor Morphology |
| B2M | beta-2-microglobulin | B2MG_RAT | √ | √ | √ | √ | √ | disease progression, efficacy, safety, unspecified application | Antimicrobial Response, Cancer, Cardiovascular Disease, Connective Tissue Disorders, Dermatological Diseases and Conditions, Developmental Disorder, Endocrine System Disorders, Gastrointestinal Disease, Hematological Disease, Hepatic System Disease, Hereditary Disorder, Immunological Disease, Infectious Disease, Inflammatory Disease, Inflammatory Response, Metabolic Disease, Neurological Disease, Nutritional Disease, Ophthalmic Disease, Organismal Injury and Abnormalities, Psychological Disorders, Renal and Urological Disease, Reproductive System Disease, Respiratory Disease, Skeletal and Muscular Disorders, Tumor Morphology |
| COL1A1 | collagen type I alpha 1 | CO1A1_RAT | √ | √ | √ | √ | √ | diagnosis | Auditory Disease, Cancer, Cardiovascular Disease, Connective Tissue Disorders, Dental Disease, Dermatological Diseases and Conditions, Developmental Disorder, Endocrine System Disorders, Gastrointestinal Disease, Hematological Disease, Hepatic System Disease, Hereditary Disorder, Immunological Disease, Inflammatory Disease, Inflammatory Response, Metabolic Disease, Neurological Disease, Organismal Injury and Abnormalities, Reproductive System Disease, Skeletal and Muscular Disorders, Tumor Morphology |
| CP | ceruloplasmin (ferroxidase) | CERU_RAT | √ | √ | √ | √ | √ | efficacy | Cancer, Cardiovascular Disease, Dermatological Diseases and Conditions, Developmental Disorder, Endocrine System Disorders, Gastrointestinal Disease, Hematological Disease, Hepatic System Disease, Hereditary Disorder, Immunological Disease, Inflammatory Response, Metabolic Disease, Neurological Disease, Nutritional Disease, Ophthalmic Disease, Organismal Injury and Abnormalities, Psychological Disorders, Renal and Urological Disease, Reproductive System Disease, Skeletal and Muscular Disorders |
| CSF1 | colony stimulating factor 1 | CSF1_RAT | √ | √ | √ | √ | √ | diagnosis, disease progression, efficacy, prognosis, unspecified application | Cancer, Cardiovascular Disease, Connective Tissue Disorders, Dermatological Diseases and Conditions, Developmental Disorder, Gastrointestinal Disease, Hematological Disease, Hepatic System Disease, Hereditary Disorder, Hypersensitivity Response, Immunological Disease, Infectious Disease, Inflammatory Disease, Inflammatory Response, Metabolic Disease, Neurological Disease, Ophthalmic Disease, Organismal Injury and Abnormalities, Renal and Urological Disease, Reproductive System Disease, Respiratory Disease, Skeletal and Muscular Disorders, Tumor Morphology |
| CSTB | cystatin B | CYTB_RAT | √ | √ | √ | √ | √ | diagnosis | Cancer, Dermatological Diseases and Conditions, Developmental Disorder, Gastrointestinal Disease, Hereditary Disorder, Infectious Disease, Inflammatory Disease, Neurological Disease, Ophthalmic Disease, Organismal Injury and Abnormalities, Psychological Disorders |
| CTSC | cathepsin C | CATC_RAT | √ | √ | √ | √ | √ | unspecified application | Cancer, Cardiovascular Disease, Connective Tissue Disorders, Dental Disease, Dermatological Diseases and Conditions, Developmental Disorder, Gastrointestinal Disease, Hepatic System Disease, Hereditary Disorder, Immunological Disease, Infectious Disease, Inflammatory Disease, Inflammatory Response, Metabolic Disease, Nutritional Disease, Organismal Injury and Abnormalities, Renal and Urological Disease, Reproductive System Disease, Skeletal and Muscular Disorders |
| EGF | epidermal growth factor | EGF_RAT | √ | √ | √ | √ | √ | diagnosis, efficacy, response to therapy, unspecified application | Cancer, Connective Tissue Disorders, Dermatological Diseases and Conditions, Developmental Disorder, Endocrine System Disorders, Gastrointestinal Disease, Hematological Disease, Hepatic System Disease, Hereditary Disorder, Immunological Disease, Infectious Disease, Inflammatory Disease, Inflammatory Response, Neurological Disease, Nutritional Disease, Ophthalmic Disease, Organismal Injury and Abnormalities, Renal and Urological Disease, Reproductive System Disease, Respiratory Disease, Skeletal and Muscular Disorders, Tumor Morphology |
| EZR | ezrin | EZRI_RAT | √ | √ | √ | √ | √ | prognosis | Cancer, Dermatological Diseases and Conditions, Developmental Disorder, Gastrointestinal Disease, Hematological Disease, Hereditary Disorder, Immunological Disease, Neurological Disease, Organismal Injury and Abnormalities, Renal and Urological Disease, Reproductive System Disease, Respiratory Disease, Tumor Morphology |
| FABP7 | fatty acid binding protein 7 | FABP7_RAT | √ | √ |  |  |  | diagnosis, unspecified application | Cancer, Dermatological Diseases and Conditions, Developmental Disorder, Endocrine System Disorders, Gastrointestinal Disease, Hepatic System Disease, Hereditary Disorder, Immunological Disease, Metabolic Disease, Neurological Disease, Organismal Injury and Abnormalities, Psychological Disorders, Reproductive System Disease, Skeletal and Muscular Disorders |
| GSTO1 | glutathione S-transferase omega 1 | GSTO1_RAT | √ | √ | √ | √ | √ | unspecified application | Cancer, Cardiovascular Disease, Dermatological Diseases and Conditions, Endocrine System Disorders, Gastrointestinal Disease, Hepatic System Disease, Immunological Disease, Infectious Disease, Inflammatory Disease, Inflammatory Response, Metabolic Disease, Neurological Disease, Organismal Injury and Abnormalities, Psychological Disorders, Renal and Urological Disease, Reproductive System Disease, Respiratory Disease |
| ICAM1 | intercellular adhesion molecule 1 | ICAM1_RAT | √ | √ | √ | √ | √ | diagnosis, efficacy, prognosis, unspecified application | Antimicrobial Response, Auditory Disease, Cancer, Cardiovascular Disease, Connective Tissue Disorders, Dermatological Diseases and Conditions, Developmental Disorder, Endocrine System Disorders, Gastrointestinal Disease, Hematological Disease, Hepatic System Disease, Hereditary Disorder, Hypersensitivity Response, Immunological Disease, Infectious Disease, Inflammatory Disease, Inflammatory Response, Metabolic Disease, Neurological Disease, Nutritional Disease, Ophthalmic Disease, Organismal Injury and Abnormalities, Psychological Disorders, Renal and Urological Disease, Reproductive System Disease, Respiratory Disease, Skeletal and Muscular Disorders, Tumor Morphology |
| LCN2 | lipocalin 2 | NGAL_RAT | √ | √ | √ | √ | √ | diagnosis, unspecified application | Antimicrobial Response, Cancer, Cardiovascular Disease, Connective Tissue Disorders, Dermatological Diseases and Conditions, Developmental Disorder, Endocrine System Disorders, Gastrointestinal Disease, Hematological Disease, Hereditary Disorder, Hypersensitivity Response, Immunological Disease, Infectious Disease, Inflammatory Disease, Inflammatory Response, Metabolic Disease, Neurological Disease, Organismal Injury and Abnormalities, Psychological Disorders, Renal and Urological Disease, Reproductive System Disease, Respiratory Disease, Skeletal and Muscular Disorders, Tumor Morphology |
| LGALS1 | lectin, galactoside binding soluble 1 | LEG1_RAT | √ | √ | √ |  | √ | diagnosis, prognosis | Cancer, Cardiovascular Disease, Connective Tissue Disorders, Dermatological Diseases and Conditions, Developmental Disorder, Endocrine System Disorders, Gastrointestinal Disease, Hematological Disease, Immunological Disease, Infectious Disease, Inflammatory Disease, Inflammatory Response, Metabolic Disease, Neurological Disease, Ophthalmic Disease, Organismal Injury and Abnormalities, Psychological Disorders, Reproductive System Disease, Respiratory Disease, Skeletal and Muscular Disorders, Tumor Morphology |
| LGALS3BP | lectin, galactoside binding soluble 3 binding protein | LG3BP_RAT | √ | √ | √ | √ | √ | prognosis | Cancer, Cardiovascular Disease, Connective Tissue Disorders, Dermatological Diseases and Conditions, Endocrine System Disorders, Gastrointestinal Disease, Hematological Disease, Hereditary Disorder, Immunological Disease, Inflammatory Response, Organismal Injury and Abnormalities, Reproductive System Disease |
| LRP2 | LDL receptor related protein 2 | LRP2_RAT | √ | √ | √ | √ | √ | diagnosis | Auditory Disease, Cancer, Cardiovascular Disease, Connective Tissue Disorders, Dermatological Diseases and Conditions, Developmental Disorder, Endocrine System Disorders, Gastrointestinal Disease, Hematological Disease, Hepatic System Disease, Hereditary Disorder, Immunological Disease, Inflammatory Disease, Inflammatory Response, Metabolic Disease, Neurological Disease, Ophthalmic Disease, Organismal Injury and Abnormalities, Psychological Disorders, Renal and Urological Disease, Reproductive System Disease, Respiratory Disease, Skeletal and Muscular Disorders |
| PRDX5 | peroxiredoxin 5 | PRD√5_RAT | √ | √ | √ |  | √ | unspecified application | Cancer, Connective Tissue Disorders, Endocrine System Disorders, Gastrointestinal Disease, Immunological Disease, Inflammatory Disease, Inflammatory Response, Organismal Injury and Abnormalities, Reproductive System Disease, Skeletal and Muscular Disorders |
| RBP1 | retinol binding protein 1 | RET1_RAT | √ | √ | √ | √ |  | diagnosis | Cancer, Developmental Disorder, Endocrine System Disorders, Gastrointestinal Disease, Hematological Disease, Hereditary Disorder, Inflammatory Response, Metabolic Disease, Nutritional Disease, Ophthalmic Disease, Organismal Injury and Abnormalities, Renal and Urological Disease, Reproductive System Disease |
| TXN | thioredoxin | THIO_RAT | √ | √ | √ | √ | √ | efficacy, unspecified application | Cancer, Cardiovascular Disease, Connective Tissue Disorders, Dermatological Diseases and Conditions, Developmental Disorder, Endocrine System Disorders, Gastrointestinal Disease, Hepatic System Disease, Hereditary Disorder, Hypersensitivity Response, Immunological Disease, Infectious Disease, Inflammatory Disease, Inflammatory Response, Metabolic Disease, Neurological Disease, Nutritional Disease, Ophthalmic Disease, Organismal Injury and Abnormalities, Psychological Disorders, Reproductive System Disease, Respiratory Disease, Skeletal and Muscular Disorders, Tumor Morphology |
| VCAM1 | vascular cell adhesion molecule 1 | VCAM1_RAT | √ | √ | √ | √ | √ | diagnosis, disease progression, efficacy, prognosis, unspecified application | Cancer, Cardiovascular Disease, Connective Tissue Disorders, Dermatological Diseases and Conditions, Developmental Disorder, Endocrine System Disorders, Gastrointestinal Disease, Hematological Disease, Hepatic System Disease, Hereditary Disorder, Hypersensitivity Response, Immunological Disease, Inflammatory Disease, Inflammatory Response, Metabolic Disease, Neurological Disease, Ophthalmic Disease, Organismal Injury and Abnormalities, Renal and Urological Disease, Reproductive System Disease, Respiratory Disease, Skeletal and Muscular Disorders, Tumor Morphology |
