# Supplementary material for: Dynamic changes of urine proteome in a Walker 256 tumor‐bearing rat model
Source: Cancer Med. 2017 Oct 4;6(11):2713–22. doi: 10.1002/cam4.1225 (PMC5673914; doi:10.1002/cam4.1225)
Supplement: Supplementary file 4 — Figure S1. The body weights of tumor‐bearing rats and HE staining of tumor tissues. Figure S2. Dynamic changes in protein patterns in the urine of tumor‐bearing rats. [file CAM4-6-2713-s001.pdf]

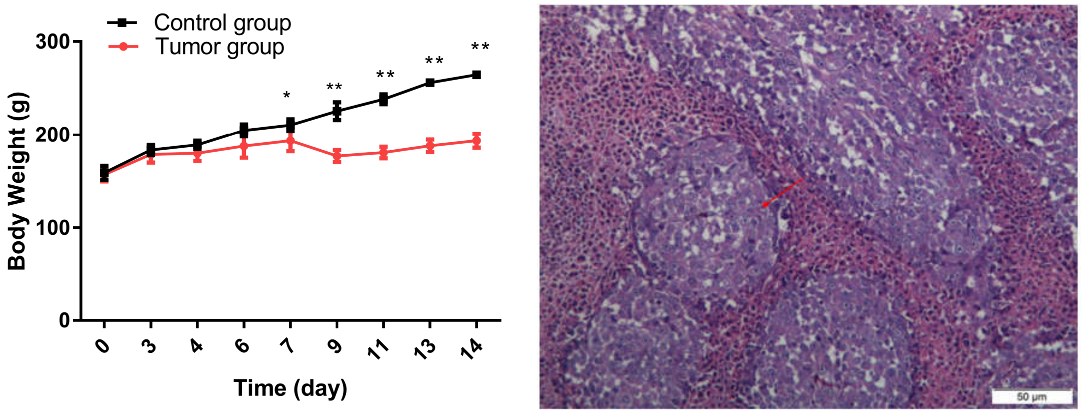

Figure S1. The body weights of tumor-bearing rats and HE staining of tumor tissues. (A) Body weights of Walker 256 tumor-bearing rats. * $p < 0.05$; ** $p < 0.01$ (B) Pathological morphologies of tumor masses on day 15 after tumor cell inoculation (HE staining, 100×); The red arrow indicates the cancer tissues.

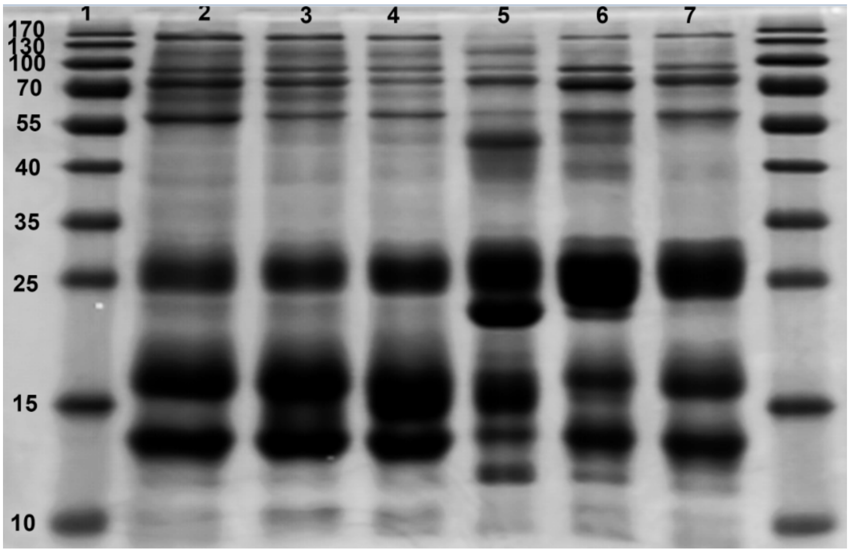

Figure S2. Dynamic changes in protein patterns in the urine of tumor-bearing rats. This figure is a representative diagram of tumor-bearing rats. Lane 1: Marker, 10-170 kDa. Lanes 2–7, urinary proteins on day 0 (lane 2), day 4 (lane 3), day 6 (lane 4), day 9 (lane 5), day 11 (lane 6), and day 14 (lane 7) after tumor cell inoculation, respectively.
